# Synthesis, spectral, nonlinear optical properties, molecular docking and cytotoxicity studies on some metal complexes derived from 2-Cyano-N’-(2-Hydroxybenzylidene)-3-Phenylacrylohydrazide 

**DOI:** 10.1038/s41598-026-54876-4

**Published:** 2026-07-20

**Authors:** Mazen A. El-Razky, Rozan Zakaria, Mohamed Abd El-Moneim, Nasser Mohammed Hosny

**Affiliations:** Chemistry Department, Faculty of Science, Port Said University, Port Said, Egypt

**Keywords:** 2-cyano-N'-(2-hydroxybenzylidene)-3-phenylacrylohydrazide, Metal complexes, NLO, Molecular docking, Cytotoxicity, Biochemistry, Cancer, Chemical biology, Chemistry, Computational biology and bioinformatics, Drug discovery

## Abstract

The synthesis and investigation of 2-cyano-N’-(2-hydroxybenzylidene)-3-phenylacrylohydrazide (H_2_L) and its Ni^2+^, Cu^2+^, Co^2+^ and Zn^2+^ complexes are discussed in this paper. These compounds were characterized with different techniques, including CHN, IR, MS, ^13^C & ^1^HNMR, TGA, ESR, UV-Visible and the magnetic moments. DFT calculations (DFT/B3LYP) level was used for geometry optimization of the suggested structures with 6-311 + + G(d, p) basis set. An octahedral stereochemistry was suggested for Ni^2+^ and Co^2+^ complexes while; a square planar geometry was proposed for Cu^2+^complex. The results proposed four coordinated stereochemistry for Zn^2+^ complex. The IR spectra of the H_2_L were simulated and compared with the experimental result, achieving a correlation coefficient R^2^ = 0.99976. The UV-visible spectra were used in determining the optical band gaps and found to be in the range 3.26–3.28 eV. Non-linear optical (NLO) properties of the DFT optimized compounds were investigated and indicated higher values in comparison with urea. The molecular docking was utilized to study the possible interactions between the synthesized compounds and the targeted proteins of liver and colon cancer. The suggested compounds were examined towards HeP2G and HCT-116 cell lines to determine their anticancer activity that are compared to Doxorubicin and Sorafenib as standards. H_2_L exhibited strong cytotoxicity against HeP2G cell line; on the other hand, Zn^2+^ complex exhibited strong cytotoxicity against HCT-116 cell line.

**Supplementary Information:**

The online version contains supplementary material available at 10.1038/s41598-026-54876-4.

Cancer is characterized by the uncontrolled proliferation of cells that propagates rapidly throughout the body. It is a primary cause of mortality globally, accounting for over 10 million deaths annually. Numerous cancer types can be healed if identified at an early stage and treated appropriately^1^. This highlights the need for more innovative and dynamic medications. Cancer drug resistance is the cause for approximately 90% of mortality related to cancer^2,3^. Cancer medication resistance and tumor angiogenesis are strongly dependent processes that together prevent efficient cancer care. Cancer cells develop strategies to resist the cytotoxic impact of therapy, like chemotherapy and selective treatments, while simultaneously encouraging angiogenesis to get the oxygen and nutrients essential for continuous growth and metastasis^4,5^. Pharmaceutical applications of Schiff bases should be prioritized, and Schiff base chemistry is a highly sought-after field in contemporary medicine. Schiff bases are good ligands containing an azomethine group. Schiff bases, that contain diazole and phenol rings, have several biological uses, including antioxidant, anticancer, antibacterial, antipyretic, anti-inflammatory, and antifungal properties^6^. The consequent C = N (azomethine) bond is the characteristic structural feature of these compounds. A strong Lewis basic (electron-donating) site is presented by the nitrogen atom of azomethine, and Schiff bases often have additional donor atoms in close proximity to them (such as OH or SH groups), which causes the ligands to be multidentate^7^. As a consequence of this, Schiff base ligands are able to chelate transition-metal ions with relative ease, resulting in the formation of stable mono- or polynuclear complexes that have well defined chelation rings. Schiff base chemistry has become widespread in synthetic inorganic chemistry as a result of the combination of facile synthesis and potent metal–ligand interaction. A substantial body of research has shown that Schiff bases have the ability to stabilize a wide range of transition-metal cores and oxidation states^8^. Metal ions possess the ability to improve the efficacy of organic molecules by the formation of metal complexes^1^. Schiff base metal complexes have significant medicinal uses owing to their broad-spectrum antioxidant, anticancer, and antibacterial properties^9,10^. Coordination to a metal center may alter the ligand’s physical and chemical properties, improving stability, bioavailability, and solubility. These changes may enhance molecular recognition and target specificity in biological organisms. Metal ions may enhance cellular uptake and transport of active molecules. The ability to modify both the ligand structure and metal allows for fine control over chemical reactivity and ebase complexes. The Schiff base’s biological efficacy was improved upon complex formation, according to in vitro biological assessments (antioxidant and antibacterial). The metal complexes of hydrazides have stronge antioxidant activity and low IC_50_ value^11,12^. Colon cancer is a leading cause of cancer-related deaths globally^13^. Chemotherapy may induce significant internal and gastrointestinal side effects, and tumors often return owing to medication resistance^14,15^. The extensive uses of Schiff bases and related metal complexes in the spectrum of biological, catalytic and pharmacological activity have encouraged researchers to synthesize new precursors to develop a systematic library of Schiff base ligands and their metal complexes^16^. Hydrazones’ capacity to chelate transition metal ions is due to easy keto-enol tautomerization caused by electron delocalization between the two hydrazinic amino groups and the surrounding carbonyl group. They have shown exceptional biological activity as anticancer, antibiotics, antifungals, and treatments for leprosy and psychiatric disorders^17^. It is important for humans to have transition metals like Co^2+^, Cu^2+^, Zn^2+^, and Ni^2+^ because they are parts of metalloenzymes and cofactors of reactive enzymes. They are also important because they are vitamins that control nucleic acid replication, transcription, and repair. These transition metals were especially intriguing for the development of anticancer medicines with various modes of action, since they may bind to different bioactive ligands with multiple oxidation states and coordination numbers. The current research intends to synthesize and characterize a new Schiff base ligand and its Zn^2+^, Co^2+^, Cu^2+^, and Ni^2+^ complexes. Extensive experimental and theoretical studies were carried out to characterize the compounds’ chemical structures. The electronic characteristics of the compounds were evaluated by DFT calculation. The semiconducting nature was tested by determining the optical band gaps. In addition to that, the cytotoxicity against colon and liver cancer cells, as well as their molecular interactions with relevant proteins was investigated. The ligand and Zn^2+^ complex exhibited promising results.

## Experimental

### Methods and materials

Every used reagent is of the analytical grade. The following substances were acquired from Aldrich: benzaldehyde (99%), ethylcyanoacetate (98%), hydrazine hydrate (100%), salicylaldehyde (99%), and glacial acetic acid (99%). All of these substances were used without any further purification. The cell lines were acquired from the American Type Culture Collection. The cell lines that were evaluated were colorectal carcinoma colon cancer abbreviated to HCT-116 and hepatocellular carcinoma abbreviated to HePG2. The anticancer medications Doxorubicin and Sorafenib were employed as the benchmark drugs for the purpose of comparison. RPMI-1640 medium, MTT and DMSO (Sigma Company, St. Louis, United States), and fetal bovine serum (GIBCO, United Kingdom) are the reagents that are being used. A CHN analyzer, which is a version 2400 of the Perkin-Elmer manufacturer, was used in order to determine the levels of hydrogen and carbon. The metal content was determined by the use of well-established conventional techniques, namely complexmetric titration, which made use of metallochromic markers such as xylenol orange, EBT, and murexide^18^. IR spectra were produced using potassium bromide plates on a Thermo Nicolet IS10 spectrometer. The verification of the frequency measurement was accomplished using polystyrene film. The electronic spectra of the ligands and associated complexes were acquired in DMSO using the Unicam UV-Vis spectrometer UV2 employing 1 cm stoppered silica cells. Magnetic susceptibility tests were done using a Sherwood Scientific magnetic balance. The thermal analyses (TG) of the compound and the various metal complexes were done using a Schimadzu Model 50 equipment. Samples were subjected to a heating acceleration of 10 °C/min from the ambient temperature to 800 °C under a 20 cm3/min nitrogen stream. ESR spectrum of Cu^2+^ complex was recorded using a Bruker E 500 spectrometer operating in the X-band (9.808 GHz) utilizing 100 kHz. The mass spectra of the compounds and the metal complexes were acquired at 70 electron volts using a Varian MAT 311 analyzer. The^1^ H and^13^ C NMR spectra of the compounds and their metal complexes in d6-DMSO were collected on a Bruker Ascend 400 MHz spectrometer.

### Synthesis of 2-cyano-N’-(2-hydroxybenzylidene)-3-phenylacrylohydrazide (H_2_L) and its coordination compounds

After dissolving 2-cyano-3-phenylacrylohydrazide (0.02 mol; 3.74 g) in 20 mL of 100% C_2_H_5_OH and adding salicylaldehyde (0.021 mol; 2.25 mL) in an ice bath, two drops of glacial acetic acid were added. After five hours of refluxing, the reaction was allowed to cool. After being separated as yellow crystals, the product was cleaned using C_2_H_5_OH and diethyl ether before being allowed to dry in the open (Scheme 1).

After dissolving the calculated amount (0.01 mol) of the acetates of Ni^2+^, Zn^2+^, Co^2+^ and Cu^2+^ in 30 mL absolute ethanol. H_2_L (0.01 mol), was added to the metal salt solutions. The mixtures were refluxed for four to six hours. The isolated precipitates were then washed with absolute EtOH and diethyl ether. The precipitates were dried for two hours at 100 °C in an oven. (Scheme 1) shows a hypothesized route of ligand chelation with each metal ion.

**Figure:**
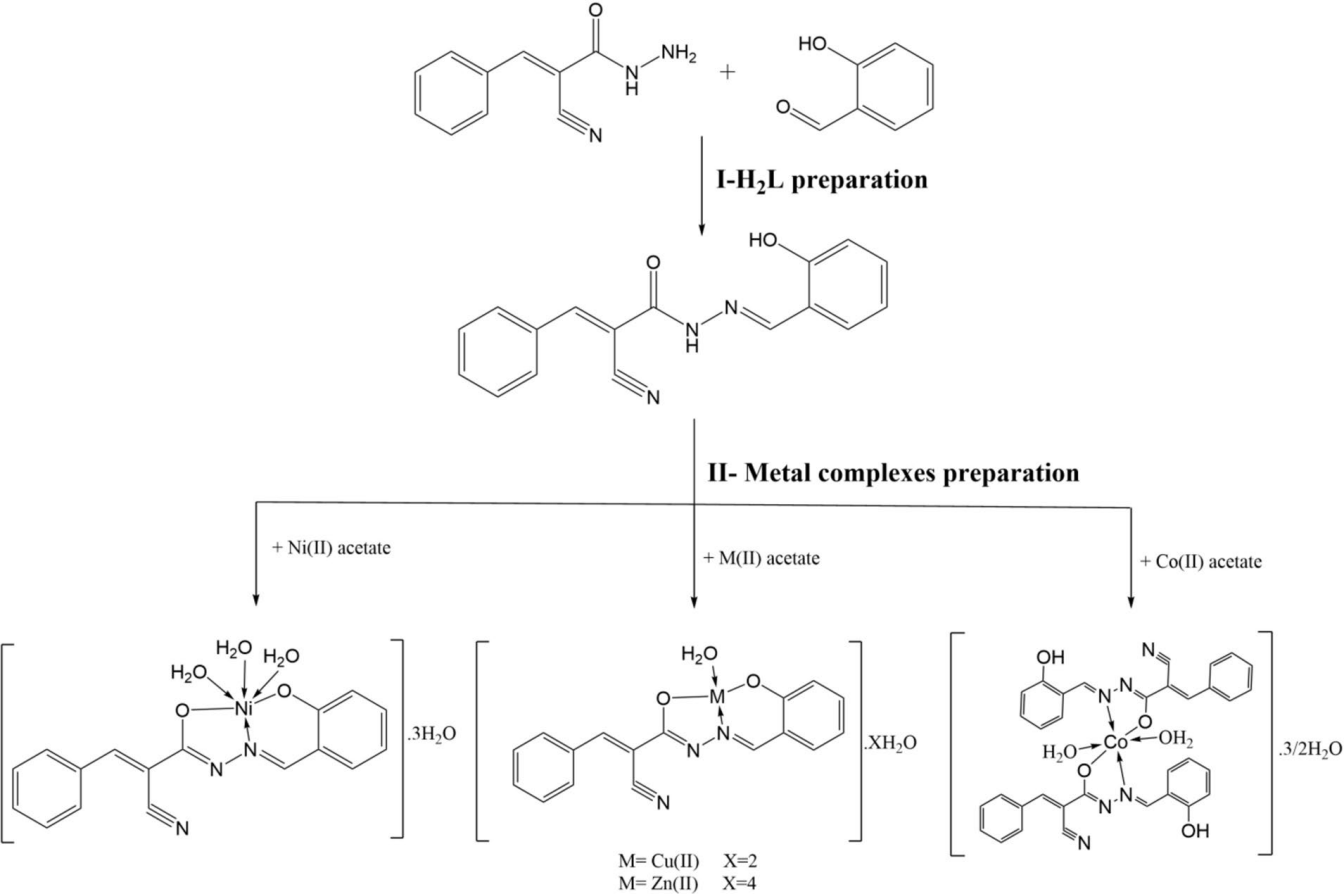
Scheme 1.

### Molecular modeling

Gaussian 03 W. programs and GaussView^19^ were used to optimize the geometry and simulate the spectra using computational calculations for the suggested compounds. Using the 6-311 + + G (d, p) basis set, the B3LYP (Becke3–Lee–Yang–Parr) method^20^ incorporates IEF-PCM (polarizable continuum model). Gauss-Sum 2.2 software^21^ was used to calculate the groups’ participation to the molecules’ orbitals.

### Molecular docking method

Molecular docking was carried out by MOE 2019.102 platform. The expected interactions between H_2_L and its coordination compounds with the corresponding receptors of liver cancer protein; PDB code = 4fm9, and colon cancer protein; PDB code = 3ig7^1,22^, were investigated. The Protein Data Bank (https://www.rcsb.org) supplied the three-dimensional protein structures as PDB data files. The structures and charges had been corrected, and the solvent molecules had been eliminated. The Triangle Matcher was used to get the docking results on a stiff protein. The docking score S was computed using the London dG technique to get 30 postures, from which the top 5 positions were selected.

### Cytotoxicity analysis

The MTT test was used to evaluate the inhibitory effect of chemicals on cellular growth using the previously indicated cell lines. The spectrophotometric method relies on the mitochondrial succinate dehydrating enzyme in live cells converting yellow tetrazolium bromide (MTT) into the formazan derivative with the purple color. RPMI-1640 medium complemented with fetal bovine serum (10%) was utilized to grow the tested cell lines. Penicillin (100 units/mL) and Streptomycin (100 µg/mL) were incubated at 37 °C with CO_2_ (5%). The cell lines were planted (1.0 × 10^4^ cells per well in a 96-well plate). They were incubated for two days at 37 °C at the same previous conditions. After that, the cells were subjected to various chemical concentrations and left for a whole day. MTT solution (20 µL; 5 mg/mL) was added and incubated for four hours after a day of medication administration. To dissolve the resulting purple formazan, 100 µL of DMSO were added to each well. To quantify and record the absorbance at 570 nm, a plate reader (EXL 800, USA) is used. According to Denizot and Lang (1986) and Mitra et al. (2016)^23,24^, the relative vitality of the cells according to the equation:

$${\rm Treated \:sample/Untreated \:sample\: x \:100}$$

### The stability of the metal complexes under biological conditions

To elucidate the stability of the investigated metal complexes under physiological conditions, the have been studied for 24 h at pH 2 and 8 using HCl and NaOH. The other conditions as the presence of foreign metal ions as Ca^2+^, Mg^2+^, Na^+^ and K^+^. All the tests were carried out at 37 °C. The studied complexes did not suffer from any change under the physiological conditions, except Ni^2+^ and Co^2+^ complexes that suffered from change in color at pH 2 after 24 h it may be due to protonation of the amide nitrogen.

### IR spectra

**Fig. 1:**
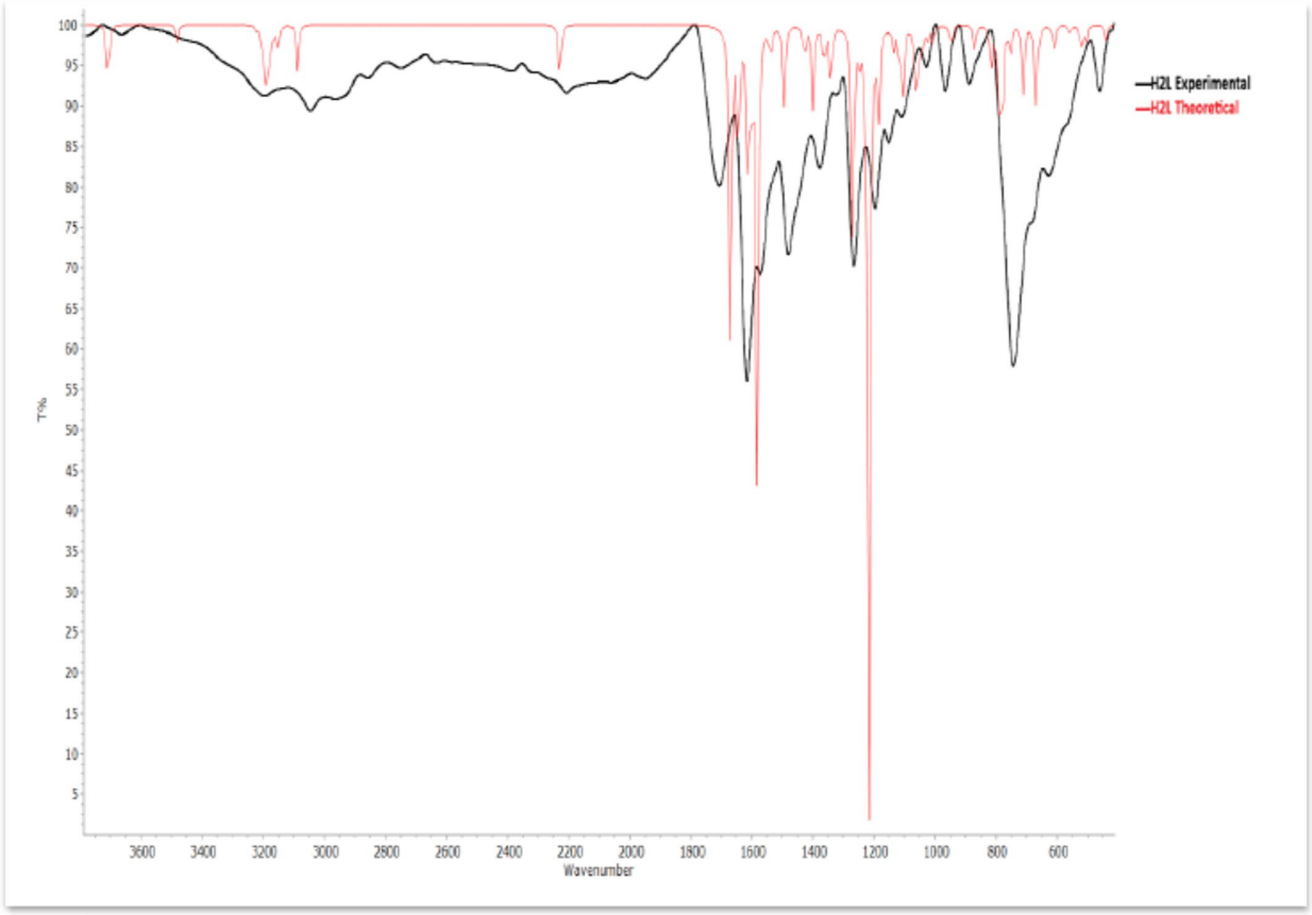
A comparison between experimental and theoretical IR spectra of H_2_L.

The ligand 2-cyano-N’-(2-hydroxybenzylidene)-3-phenylacrylohydrazide (H_2_L) can occur in either keto or enol forms (Scheme S1). A comparison of the observed and theoretically computed IR spectra *via* DFT was performed (Fig. 1). The correlation coefficient between selected bands in the experimental and theoretical infrared spectra is computed. The R^2^ = 0.99976, as shown at (Fig. S1).

The stretching vibrations of the phenolic v(OH) and δ(NH) are represented as bands at 3343 and 3202 cm^− 1^ in the infrared spectra of H_2_L (in KBr)^25^. The theoretically predicted spectra show these bands at 3713 and 3481 cm^− 1^, respectively. About 2000 cm^− 1^, a number of faint bands are observed, suggesting the potential of hydrogen bonding. The band of v(C ≡ N) emerges at 2211 cm^− 1^ (theoretically calculated at 2229 cm^− 1^). Furthermore, the band of v(C = N) occurs at 1616 cm^− 1^ (theoretically calculated at 1613 cm^− 1^). the δ(NH) band appears at 1573 cm^− 1^ (theoretically calculated at 1580 cm^− 1^), the stretching band of ν(C–N) emerges at 1281 cm^− 1^ (theoretically calculated at 1273 cm^− 1^). The bands located at 1707 and 1693 cm^− 1^ are associated with ν(CONH) and v(C = O), respectively^25,26^, the theoretically calculated spectra show these bands at 1671 and 1647 cm^− 1^. The presence of the bands of ν(NH), ν(CONH), ν(C = O) and δ(C–N) indicates the presence of H_2_L in the keto form^1^. Apart from ν(C–C) ring stretches at 1527 and 1481 cm^− 1^, in-plane δ(OH) bends at 1377 cm^− 1^, ν(N–N) stretches at 1195 cm^− 1^, ν(C–O) stretches at 1157 cm^− 1^, in-plane δ(C–H) bends at 1110 and 1030 cm^− 1^, out-of-plane δ(C–H) bends at 967 and 889 cm^− 1^, and broad hydrogen bonded out-of-plane δ(OH) bends at 685 cm^[− 1 27,28^.

The coordination compounds’ spectra show that the bands for v(C = O), δ(NH), and δ(C-N) have vanished, indicating the presence of the H_2_L in metal complexes in the enol state. The absence of enolic (OH)_enol_ in metal complex spectra indicates that this group was deprotonated following metal ion chelation. A new shoulder band of ν(C = N)^*^ stretching vibrations of the Schiff base derivatives was observed in the region 1688–1708 cm⁻¹, these values were reported earlier in the region 1500–1700 cm^[− 1 29^. The highly conjugated system of the H_2_L in the enol in addition to the existence of the newly formed azomethine group in between (C = N) and (C ≡ N) groups may be responsible for the presence of the newly formed ν(C = N)^*^ at relatively high wavenumber. It is noticed the disappearing of the phenolic OH bands in the free ligand from the spectra of Ni^2+^, Cu^2+^ and Zn^2+^ complexes suggesting the participation of this group in the complexation to the M^2+^ with the liberation of hydrogen ion. On the other hand, the phenolic OH remains in its position in Co^2+^ complex suggesting the inertness of this group towards coordination. At the same time, the stretching vibrations of ν(C ≡ N) in metal complexes haven’t been shifted, showing the unchanging electron density in this active site; thus, it is not a coordination site. Several additional weak bands are seen in the ranges 583–596 and 443–497 cm^− 1^ owed to ν(M-O) and ν(M-N), respectively^25,30^. From this discussion it can be suggested that, H_2_L chelates Ni^2+^, Cu^2+^ and Zn^2+^ ions in a binegative tridentate manner coordinating *via* deprotonated enolic oxygen (ONO). In case of Co^2+^ complex, H_2_L coordinates in a mononegative bidentate manner through azomethine nitrogen (C = N) and deprotonated enolic oxygen.

A comparison between H_2_L and its metal complexes is illustrated in (Figs S2 –S3). The existence of δ(OH) as a shoulder at 964 cm^− 1^ in the Co^2+^ complex suggests the existence of a phenolic (OH) group, as it is included in a hydrogen bond. The important bands are presented in (Table S2). The overlapping bands in the 1800–1300 cm^− 1^ region were resolved through deconvolution analysis. H_2_L, Co^2+^, Ni^2+^, Cu^2+^and Zn^2+^complexes are displayed at (Figs S4 –S8) respectively^25^.

### Nuclear magnetic resonance

Five different multiplet signals, corresponding to 9 H of substituted benzene rings δ = 6.96, 7.39, 7.46, 7.69, and 7.78 ppm, are shown in the^1^ H-NMR spectra of H_2_L in DMSO-d_6_ (Fig. S9A). These signals are ascribed to (H_3_ and H_5_ of the phenol ring), (H_4_ of the phenol ring), (H_3_, H_4_ and H_5_ in the benzene ring), (H_6_ of the phenol ring), and (H_2_ and H_6_ in the benzene ring), in that order^31^. The presence of two protons from (CH = C)_vinyl_ and (CH = N) is shown by the two singlet signals δ = 8.69 and 8.91 ppm, respectively. Individual protons exhibit two singlet signals δ = 9.0 and 11.12 ppm, are associated to (NH) and phenolic (OH) functional groups, respectively. D_2_O has been used in place of the last two signals. The protons of DMSO and H_2_O exhibit singlet signals at 3.3 and 2.4 ppm, respectively^32^. The suggested ketonic structure of H_2_L is supported by the existence of the (NH) proton. [Zn(L).H_2_O]2H_2_O^1^ H-NMR spectra in DMSO-d_6_ (Fig. S9B). The deprotonation that occurs after association with the metal ion has suppressed the phenolic (OH) and (NH) signals in the Zn^2+^ complex’s spectra. The absence of the (NH) signal indicates the presence of a Zn^2+^ complex in the enol form. ^13^C-NMR (Fig. S10) offers important information about the organic ligand’s carbon structure. The DMSO-d_6_ chemical shift can be seen δ = 39.52 ppm^1^ in the H_2_L spectra. Additionally, it displays four signals δ = 163.4, 159.1, 148.5, and 117 ppm, which correspond to carbonyl carbon (C = O), (= C-OH) carbon, azomethine carbon (C = N), and cyano carbon (C ≡ N). The range of the remaining phenyl ring carbons is 134.6-116.1 ppm^31^.

### Mass spectra

Mass spectra were performed to figure out the molecular weights and probable fragmentation paths of the compounds. The mass spectrum and the fragmentation pathway of H_2_L are shown in (Fig. S11) and (Scheme S2), respectively. H_2_L has a molecular ion peak at m/z = 290.89 (20.83%), consistent with the suggested formula (M.wt = 291.31).

The mass spectrum of [Cu(L)H_2_O]2H_2_O (Fig. S12) reveals a molecular ion peak at m/z = 407.19 (6.89%), consistent with the proposed formula (M.wt = 406.88), and the anticipated fragmentation of the Cu^2+^ complex is illustrated in the accompanying (Scheme S3).

MS spectrum of [Ni(L).(H_2_O)_3_]3H_2_O (Fig. S13) reveals the molecular ion at m/z = 456.96 (12.22%), consistent with the calculated molecular weight 456.07. The anticipated fragmentation of the Ni^2+^ complex is illustrated in (Scheme S4). The mass spectrum of [Zn(L).H_2_O]2H_2_O (Fig. S14) discloses a molecular ion peak at m/z = 444.70 (35.04%), consistent with the calculated molecular weight 444.74, and the anticipated fragmentation of the Zn^2+^ complex is illustrated in (Scheme S5). MS of [Co(HL)_2_.(H_2_O)_2_]$\:\:\frac{3}{2}$ H_2_O (Fig. S15) discloses the molecular ion at m/z = 704.08 (19.62%), consistent with the proposed formula (Molecular Weight 702.59), suggesting the existence of Co^2+^ complex in form 1:2 (metal: ligand). The anticipated fragmentation of the Co^2+^ complex is illustrated in the accompanying (Scheme S6).

### Electronic spectra and the effective magnetic moments (µ_eff_.)

n→π* transition appears as a wide band at 21,682 cm^− 1^ in H_2_L spectrum in DMSO (Table S3). An octahedral arrangement around Ni^2+^ is indicated by the magnetic moment value of [Ni(L).(H_2_O)_3_]3H_2_O (**µ**_**eff.**_ = 3.31 B.M.). L→MCT band appears at 28,272 cm^− 1^ while; the transitions^3^ A_2g_ → ^3^T_1g_ (P) (υ_3_) and^3^ A_2g_ → ^3^T_1g_ (F) (υ_2_) appear at 24,096 and 15,576 cm^− 1^, respectively (Fig. S16). These observations in addition to the ligand field parameter (B = 550, β = 0.51, 10Dq = 10450 cm^− 1^, which equals (υ_1_)) support the octahedral stereochemistry of this complex^26^.

[Cu(L).H_2_O]2H_2_O has **µ**_**eff.**_ = 1.88 B.M., of an unpaired electron of d^9^ electronic configuration^33^. The electronic spectra in DMSO shows LMCT band at 27,048 cm⁻¹. The square planar d-d transitions, ^2^B_1g_→^2^E_g_ and^2^ B_1g_→^2^A_1g_, appear at 18,908 and14931 cm^− 1^, respectively^34^.

An octahedral structure is implied by the magnetic moment of [Co(HL)_2_.(H_2_O)_2_] 3/2 H_2_O (**µ**_**eff.**_ = 5.2 B.M.), corresponds to a high spin d^7^ electronic configuration. Electronic spectra that show bands at 28,321, 20,602, and 18,512 cm^− 1^ owing to LMCT, ^4^T_1g_(F) →^4^T_1g_(P) (υ_3_), and^4^ T_1g_(F) →^4^A_2g_ (υ_2_) transitions, respectively, further support this stereochemistry (Fig. S17)^34^. The range described for octahedral Co^2+^ complexes includes the ligand field parameter (B = 883, β = 0.91, 10Dq = 9718, and υ_1_ = 8534 cm^− 1^).

### ESR of the [Cu(L).H_2_O]2H_2_O complex

The stereochemistry of Cu^2+^ complex is identified from the ESR spectrum (Fig. S18) and the ground term of [Cu(L).H_2_O]2H_2_O. Cu^2+^ complex has a square-planar stereochemistry when g_||_ (2.20298) > g┴ (2.06918) > 2.0023, since the unpaired electron occupies the orbital, resulting in a ^2^B_1g_ ground state^1^. The exchange interactions are indicated by the axial symmetrical parameter (G), G parameter is determined by Eq.  G = (g_||_-2)/(g┴-2) ^35^ and it’s significant when G is lower than 4 and insignificant when G is higher than 4.0.

Cu^2+^ complex’s calculated G value is 2.93, which shows a strong interactions between the ligand and the copper ion caused electrons delocalization from Cu^2+^ to the molecular orbital^25^.

The covalency degree was determined by measuring K using the formula^36^,

$$\:\mathrm{K}\left|\right|2=\frac{\left(\mathrm{g}\right||-2.0023)}{8\:\times\:\lambda\:o}\times\:\mathrm{d}-\mathrm{d}\:$$

$$\:\mathrm{K}┴2=\frac{(\mathrm{g}┴-2.0023)}{2\:\times\:\lambda\:o}\times\:\mathrm{d}-\mathrm{d}\:$$

$${\rm K^2 = \frac{(K_\parallel ^2 + K_\perp ^2)}{2}}$$

K is the orbital reduction factor, d-d refers to the quantum leaps, λ_0_ (the spin-orbit coupling constant) is taken to be 828 cm^-1^. It was found that Cu^2+^ complex exhibits in-plane π-bonding and a strong covalent nature as${\rm K =0.81, K_\parallel = 0.67 \: and\: K_\perp =0.87}$ .

The effective magnetic moment (µ_eff_) of Cu^2+^ complex was determined from g_||_ and g┴by using formula^37^.

$$\:{\upmu\:}\mathrm{e}\mathrm{f}\mathrm{f}=\frac{1}{2}\sqrt{{\mathrm{g}\left|\right|}^{2}+2{\mathrm{g}┴}^{2}}$$

The experimental value of 1.88 B.M. and the computed µ_eff_ of 1.83 B.M. are in excellent agreement.

### Thermal gravimetrical analyses (TGA)

**Fig. 2:**
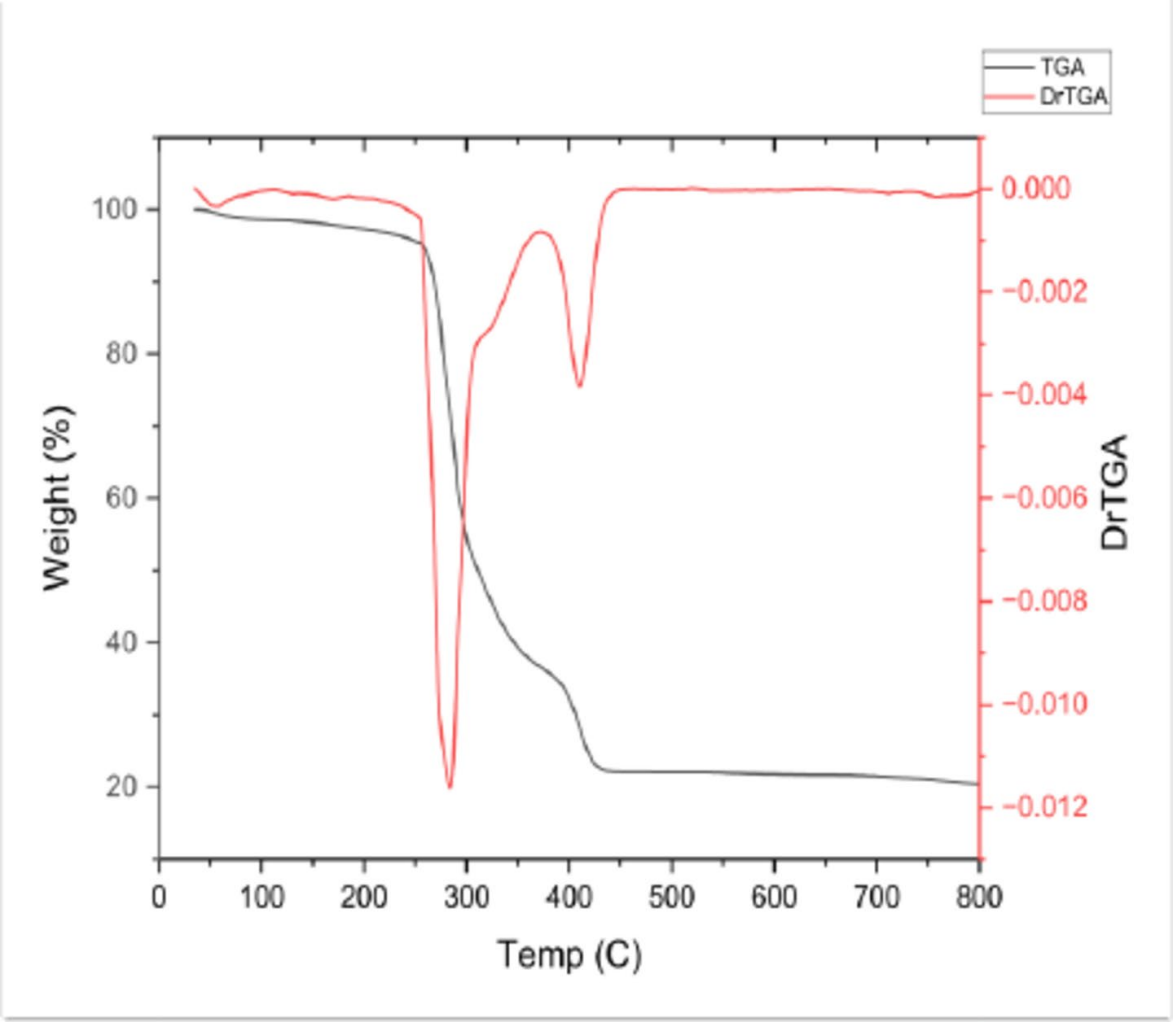
TGA curve of the [Cu(L).H_2_O]2H_2_O complex.

TGA may help in the identification of the metal chelates and give knowledge of the thermal stability of the metal complexes. TGA analyses were performed from 30 to 800 °C. [Cu(L).H_2_O]2H_2_O (Fig. 2) loses 0.5H_2_O of hydrogen bonded water in the temperature range 30 − 14 0° C (Exp.; 1.93; Calcd.; 2.21%), Then the complex loses 2.5H_2_O and the species C_7_H_4_NO in the temperature range 140–295 °C, (Exp.; 40.37; Calcd.; 40.09%). C_5_H_4_N is lost in range (295–373 °C), (Exp.; 21.31; Calcd.; 19.19%). The species C_5_H_3_N is lost in range 373–800 °C (Exp.; 19.99; Calcd.; 18.90%), sending CuO off as a residual (Exp.; 20.49; Calcd.; 19.6%) (Table S4).

[Ni(L).(H_2_O)_3_]3H_2_O decomposes thermally in five stages (Fig. S19). In the initial stage (45–120 °C), it is proposed that water of hydration is lost (1.5H_2_O) (Exp.; 5.95; Calcd.; 5.92%). The second step is an endothermic process that starts at 120 to 307 °C, all coordinated water and water of crystallization molecules (4.5H_2_O) are lost in this step (Exp.; 18.15; Calcd.; 17.78%). The third step starts at 307 to 334 °C, the molecular species C_6_H_4_NO is lost (Exp.; 22.92; Calcd.; 23.24%). The fourth step from 334 to 338 °C, the molecular species C_6_H_4_N is lost (Exp.; 21.36; Calcd.; 21.93%). The final step is the elimination of C_5_H_3_N (Exp.; 16.08; Calcd.; 16.89%), sending off NiO as a residue (Exp.; 15.7; Calcd.; 16.38%).

[Co(HL)_2_.(H_2_O)_2_]$\:\:\frac{3}{2}$ H_2_O complex decomposes in multiple steps (Fig. S20), the first decomposition begins from 36 to 90 °C, in which 1.5 molecule of hydrogen bonded water are (Exp.; 4.84; Calcd.; 3.85%). In the second decomposition, two coordinated water molecules are liberated from 90 to 190 °C; (Exp.; 5.16; Calcd.; 5.13%), followed by losing C_4_H_3_N species from 190 to 266 °C; (Exp.; 10.04; Calcd.; 9.26%), C_7_H_5_NO species from 266 to 342 °C; (Exp.; 17.01; Calcd.; 16.95%) and C_8_H_6_N_2_O (Exp.; 24.04; Calcd.; 24.07%) in the temperature range 342–359 °C. The C_7_H_5_NO species is lost from 359 to 367 °C (Exp.; 17.23; Calcd.; 16.95%), then the C_4_H_3_N species is lost from 367 to 379 °C (Exp.; 9.64; Calcd.; 9.26%), at the end, C_4_H_2_ is eliminated in the temperature range 379–800 °C (Exp.; 4.94; Calcd.; 7.12%), leaving CoO as a residue (Exp.; 11.7; Calcd.; 10.68%).

[Zn(L).H_2_O]4H_2_O decomposes in several steps (Fig. S21), the first decomposition, from 30 to 120 °C, represents the loss of water of hydration (Exp.; 4.05; Calcd.; 4.34%), then all coordinated water and water of crystallization molecules are lost (4H_2_O) (Exp.; 16.22; Calcd.; 16.22%). thirdly C_3_H_2_N is lost from 273 to 355 °C (Exp.; 12.72; Calcd.; 11.7%), followed by loss of the species C_3_H_5_ (Exp.; 11.54; Calcd.; 9.23%), in the temperature range 355–401 °C. In the 5th stage (401–418 °C), C_5_H_2_N is lost (Exp.; 16.51; Calcd.; 17.12%). The final step is the elimination of C_5_H_2_N (Exp.; 17.56; Calcd.; 17.12%), leaving ZnO (Exp.; 18.21; Calcd.; 18.21%).

### Optical band gap

The distance between the molecular orbitals that are (HOMO) and (LUMO) is called the “optical band gap” (E_g_). It is calculated from Tauc’s relation: (αhv)^n^ against hv.

**Fig. 3:**
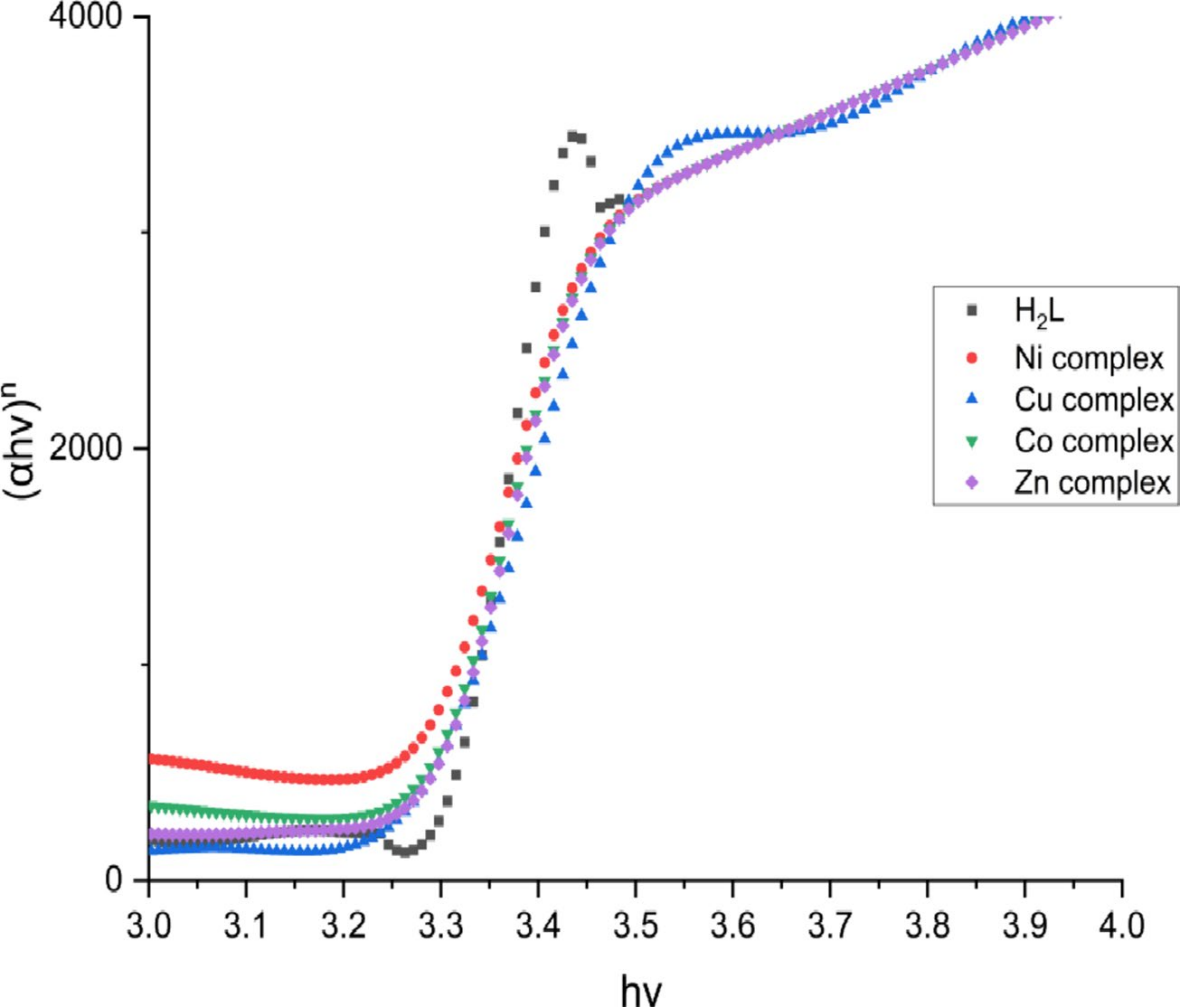
E_g_ of H_2_L and M^2+^ complexes.

Where *n* = 2 when the electronic transition is direct and 1/2 for the indirect transitions. E_g_ is computed experimentally from the UV-visible spectra of H_2_L and its metal chelates ^38^. In this relation, the photon energy is represented by (hν) and the absorption coefficient is represented by (α). The results indicate that, E_g_ of the coordination compounds are 3.26 to 3.28 eV, which is lower than that of the unbound ligand at 3.30 eV. Thus, all transitions are direct electronic transitions, as evidenced by the results (Fig. 3). The development of larger orbitals between the metal ions and the ligand is the cause of the decrease in the band gap value that occurs after the formation of a metal complex. The E_g_ values point to semiconducting materials^39,40^.

### Geometry optimization

**Fig. 4:**
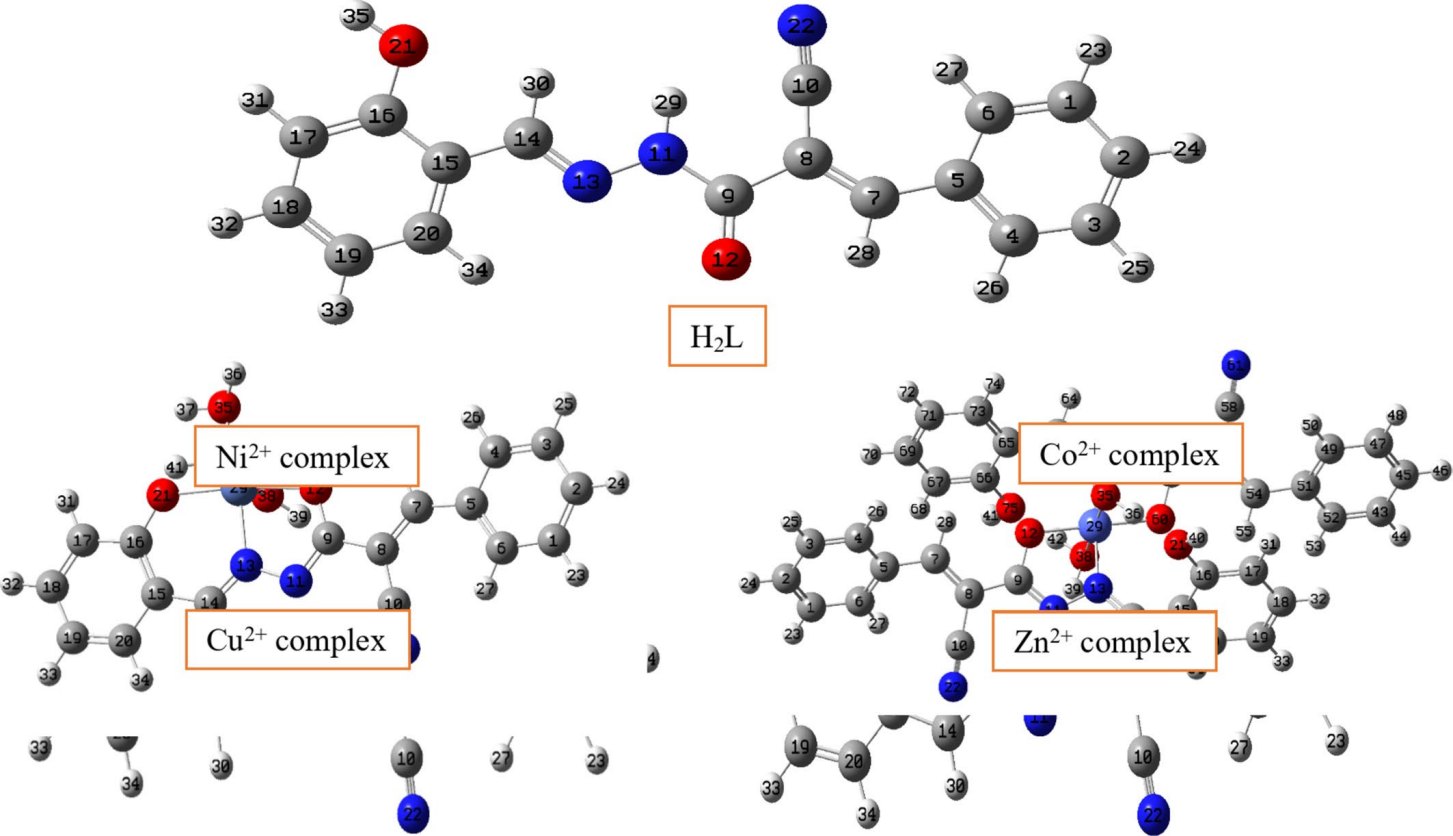
H_2_L and M^2+^ complexes’ optimized structures.

Optimization of the structures of the compounds was carried out by DFT. The optimized structure of H_2_L exhibits a planar arrangement where the bond angle H(28)-C(7)-C(8) = 112.65° derivate from the ideal 120° of the SP^2^ hybridization that permits the hydrogen bonding between H(28)-C(7) and C(9)-O(12), as depicted in Fig. 4. The results (Table S5) reveal that, the (C = O) is coplanar with H(28)-C(7) as indicated from the dihedral angle H(28)-C(7)-C(8)-CO = -0.004° that facilitates the possibility the molecular bonding (the bond length is 2.28 Å). A comparison between the obtained data with X-ray single crystals of similar analogues^26,41,42^ was carried out. The metal complexes’ optimized structures show that Co^2+^ and Ni^2+^ complexes have octahedral arrangement, whereas the Zn^2+^ and Cu^2+^ complexes have square planar arrangement. There are slight distortion in bond lengths and angles of the calculated geometries with the normal values. On the other hand, the geometrical characteristics show distortion in bond lengths approximately 0.067 Å, this slight distortion was observed also in dihedral and bond angles when comparing with the known X-ray single crystals of the analogous compounds^26,41,42^. The DFT-optimized structures for Ni^2+^, Co^2+^, Cu^2+^ and Zn^2+^ complexes are displayed at (Fig. 4). The results (Table S6) reveal that: All metal complexes show bond length distortion; specifically, the bond lengths C(16)-O(21), C(9)-O(12) and C(14) = N(13) have been changed in comparison with their lengths in the H_2_L as a result of coordination of these active sites. In case of Ni^2+^, Cu^2+^ and Zn^2+^ complexes, M-O_(Sal)_, M-N, M-OH_2_ and M-O_(enol)_ range from 1.89 to 1.996, 1.83–2.032, 1.719–2.133, and 1.933–2.065 Å, respectively. These values are in good agreement with the experimentally found analogues^26,41,42^. Additionally, bond angle measurements revealed another kind of distortion. For instance, in Zn^2+^ complex, O(H_2_O)-M-O_(Sal)_ varied from the conventional value of 90.0°in square planar arrangement to be 79.256° (Table S7), on the other hand, this angle is 83.97° in case of Cu^2+^ complex. O_(enol)_-M-O_(Sal)_ angles were 177.96° and 170.41° which are more or less distorted for Cu^2+^ and Zn^2+^, respectively in comparison with the standard 180° value for this arrangement. As well, Co^2+^ and Ni^2+^ complexes have octahedral configurations, the results display distorted bond angles, e.g., N-M-O _(enol)_ and O_(H2O)_-M-O _(enol)_ with values 81.57, 79.69° and 89.283, 79.49° for Co^2+^ and Ni^2+^ complexes, respectively. In addition to that, the dihedral angles of the Zn^2+^and Cu^2+^ complexes exhibit coplanar configuration with slight distortions in the bond lengths and angles. The higher distortion in the dihedral angles in Co^2+^ and Ni^2+^ octahedral complexes resulted from the intramolecular forces, for instance, in Co^2+^ complex, the dihedral angle N-N-Co-O_(enol)_ equals 18.057° where the complex is twisted to facilitate π-π interaction with the phenyl rings and the Van der Waals forces in the complex.

### Frontier molecular orbitals

**Fig. 5:**
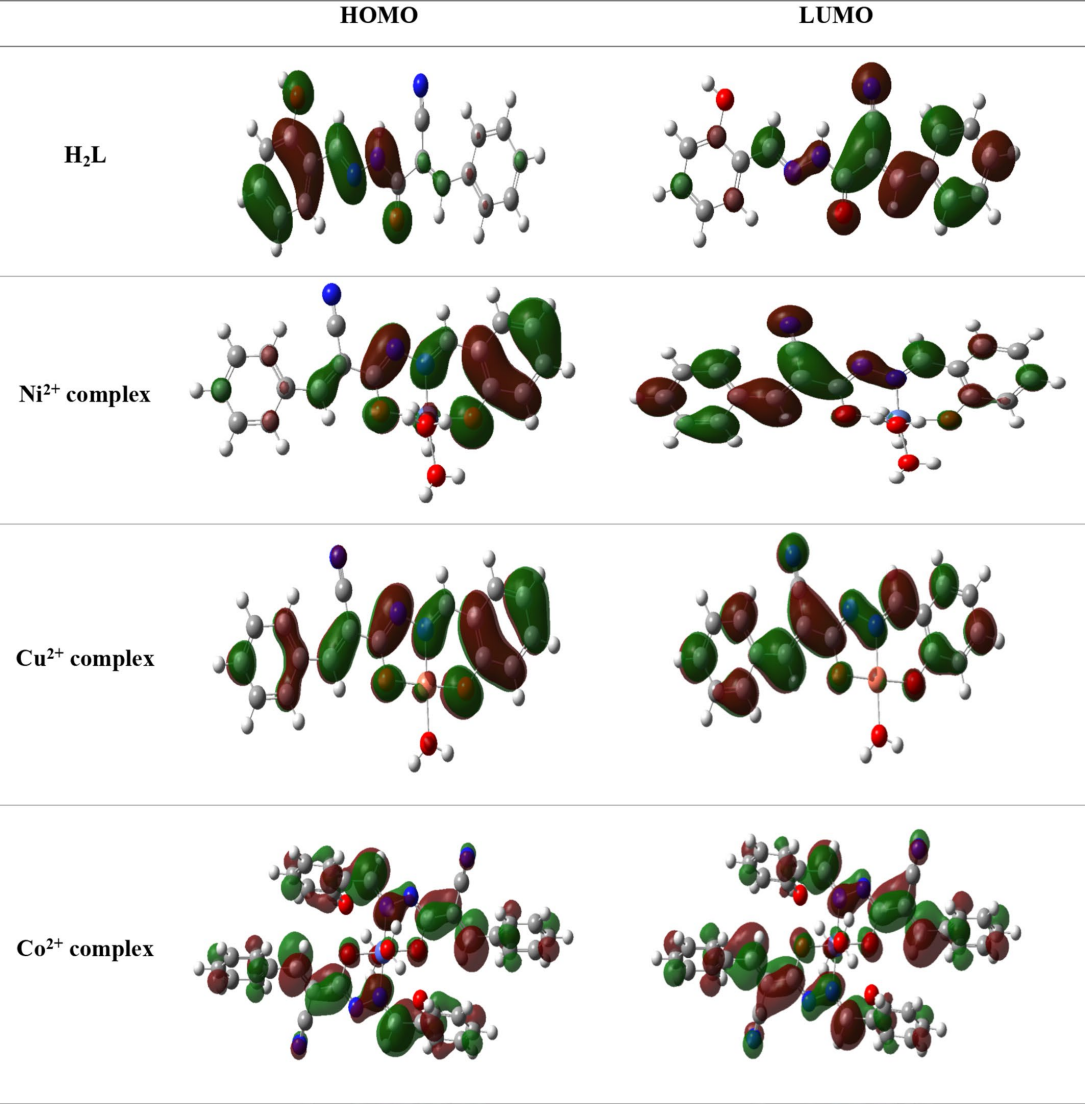

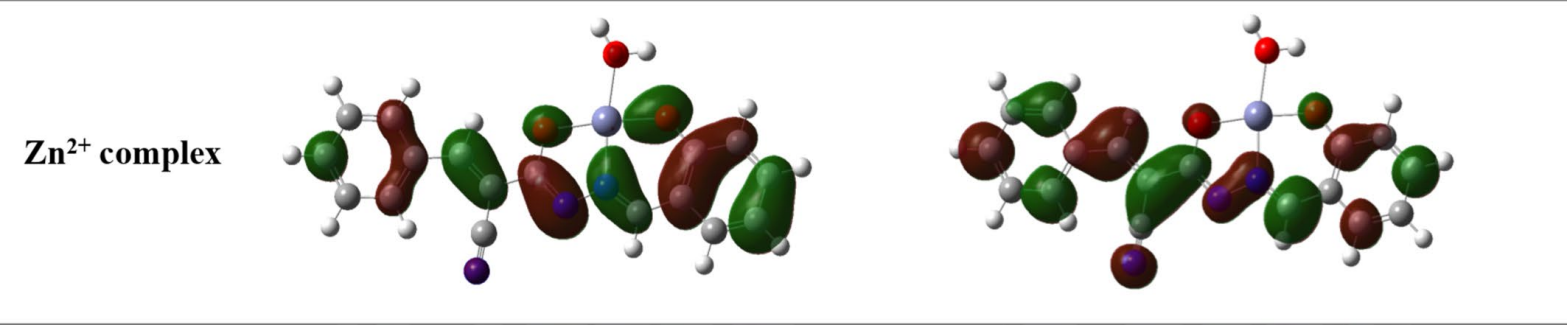
The HOMO-LUMO orbitals of H_2_L and M^2+^ complexes.

The molecule with a tight LUMO-HOMO energy gap is characterized by reduced stability, increased softness, increased reactivity, and a tendency for participating in charge transfer interactions^42,43^. The three-dimensional depiction of the ligand’s HOMO and LUMO orbitals indicates that the HOMO predominantly comprises π-orbitals alongside the nonbonding orbitals (n) of the heteroatoms, whereas the LUMO is characterized by a π*-orbital. Consequently, the charge transfer from HOMO to LUMO is attributed to π→π* and n→π* transitions (Fig. 5). The FMO plots of Co^2+^ complex indicate that its HOMOs predominantly comprise the salisoyl and phenyl π-orbitals in addition to the N and O lone pairs through the complex. In addition to that, a contribution from water oxygen’s lone pair is observed in this complex. The LUMO consists of π*-orbital of H_2_L molecules in addition to a high participation of Co^2+^ ion’s vacant orbitals are observed. In the Ni^2+^ complex, the HOMO is composed of the π-orbital of the salisoyl and azomethine moieties and the lone pairs of electrons of oxygen atoms within the ligand and water molecules, with significant contribution from the Ni^2+^ orbitals, while the LUMO is composed of the π*-orbitals across H_2_L moiety. In the Cu^2+^ and Zn^2+^ complexes, HOMO is derived from the N and O lone pairs orbitals, and the π-orbitals across H_2_L moiety significantly influenced by the M^2+^ orbitals, whereas LUMO is formed from the antibonding orbitals (π^*^) of the double bonds in conjunction with the M^2+^ orbitals (Fig. 5).

HOMO-LUMO energies indicate that the H_2_L possesses the lowest HOMO energy, E_H_ = -6.326 eV, signifying its affinity for donating electrons, and also exhibits the lowest LUMO energy, E_L_ = -2.835 eV, thereby facilitating electron acceptance. The computed ΔE_H−L_, was juxtaposed with the experimental E_g_, revealing that H_2_L displayed the maximum E_g_ of 3.3 eV, whereas the Ni^2+^ and Cu^2+^ complexes exhibited the minimum E_g_ at 3.26 eV (Table S8). Besides that, additional chemical reactivity descriptors (electronegativity (χ), global hardness (η), global softness (δ), and electrophilicity (ω)) were assessed using E_HOMO_ & E_LUMO_. These characteristics show the electron acceptance capacity, charge transfer resistance, molecular Lewis acid-base character, and energy reduction related to HOMO-LUMO electron transfer, respectively, and are computed using the following relations^44^:

χ = $$\:-\frac{1}{2}\:$$(E_HOMO_+E_LUMO_) η =$$\:-\frac{1}{2}\:$$(E_HOMO_-E_LUMO_) δ = $$\:\frac{1}{{\upeta\:}}$$ω = $$\:\frac{{{\upchi\:}}^{2}}{2{\upeta\:}}$$.

Table S8 demonstrates that Zn^2+^ complex possesses the lowest global hardness at 1.676 eV, whilst the H_2_L the highest value at 1.7455 eV. Zn^2+^ complex possesses the highest softness at 0.5967 eV, whilst the H_2_L the lowest value at 0.5729 eV.

### The molecular electrostatic potential map (MEP)

**Fig. 6:**
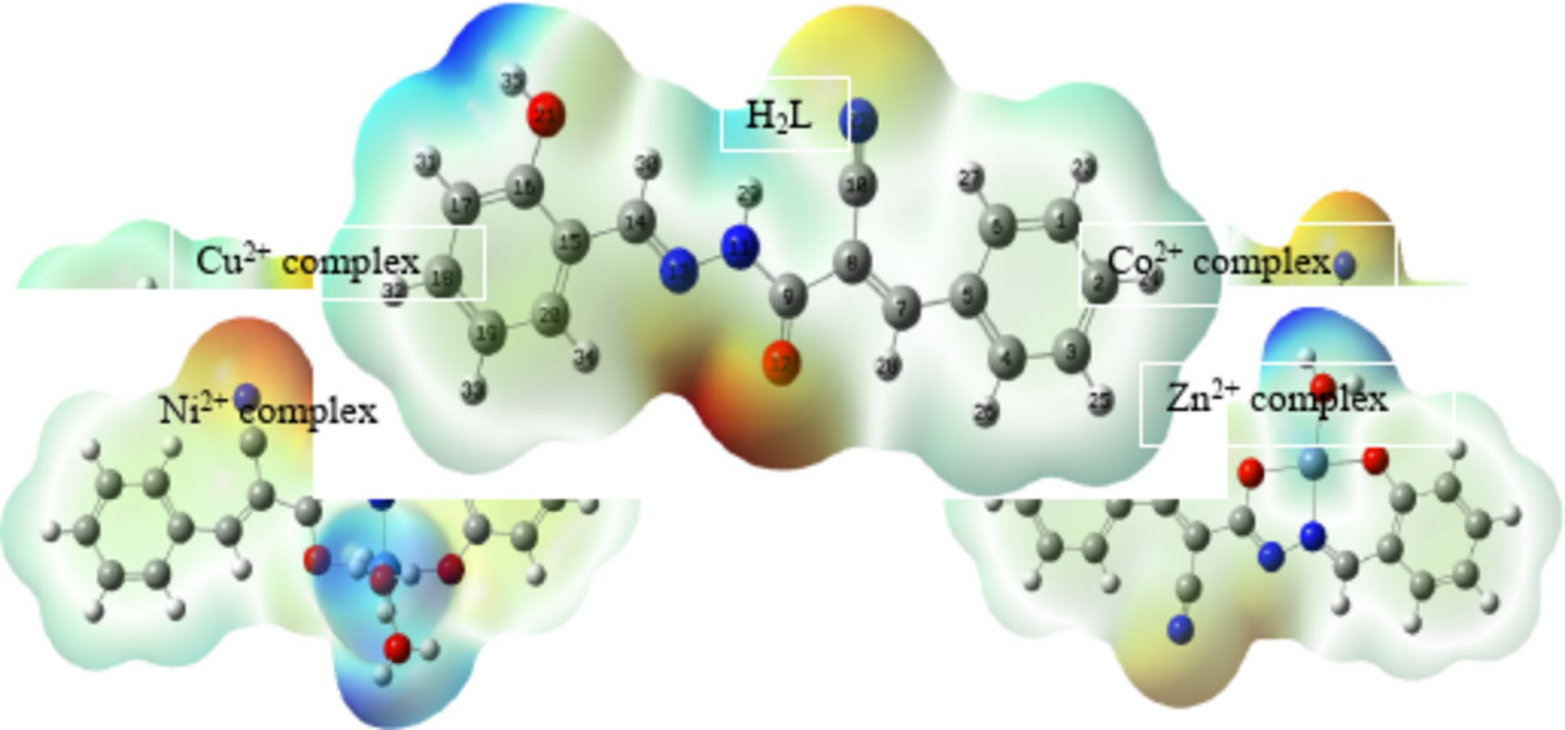
MEP of the H_2_L and its M^2+^ complexes.

The estimated electron density surface is represented by the MEP, which is color-coded. MEP surface representations in three dimensions, commonly known as the MEP map^45^. The molecule charge dispersion is shown on the MEP map. Understanding charge distribution, red designating the negative side and blue designating the positive side, is used to determine load-dependent properties and molecular interactions. (Fig. 6) shows the MEP of the H_2_L and its M^2+^ complexes. Based on the electron density, the MEP diagram shows the nucleophilic and electrophilic sites. A color gradient ranging from red to blue represents the electrostatic potential’s increasing progression^46^. The area where the value is 0 is associated with the color green. MEP maps show that regions containing oxygen or nitrogen atoms appear orange to yellow. These are the places in the ligand where there is a lot of electrophilic reactivity. However, the blue areas correspond to hydrogen atoms bound to nitrogen or oxygen atoms, resulting in a positive electron density that permits nucleophilic reactivity. The metal complexes’ MEP diagrams resemble the ligand’s MEP, in addition to the existence of water molecules where hydrogen atoms act as electron positive sites and appear as blue regions in the MEP maps.

### ELF and LOL analysis

**Fig. 7:**
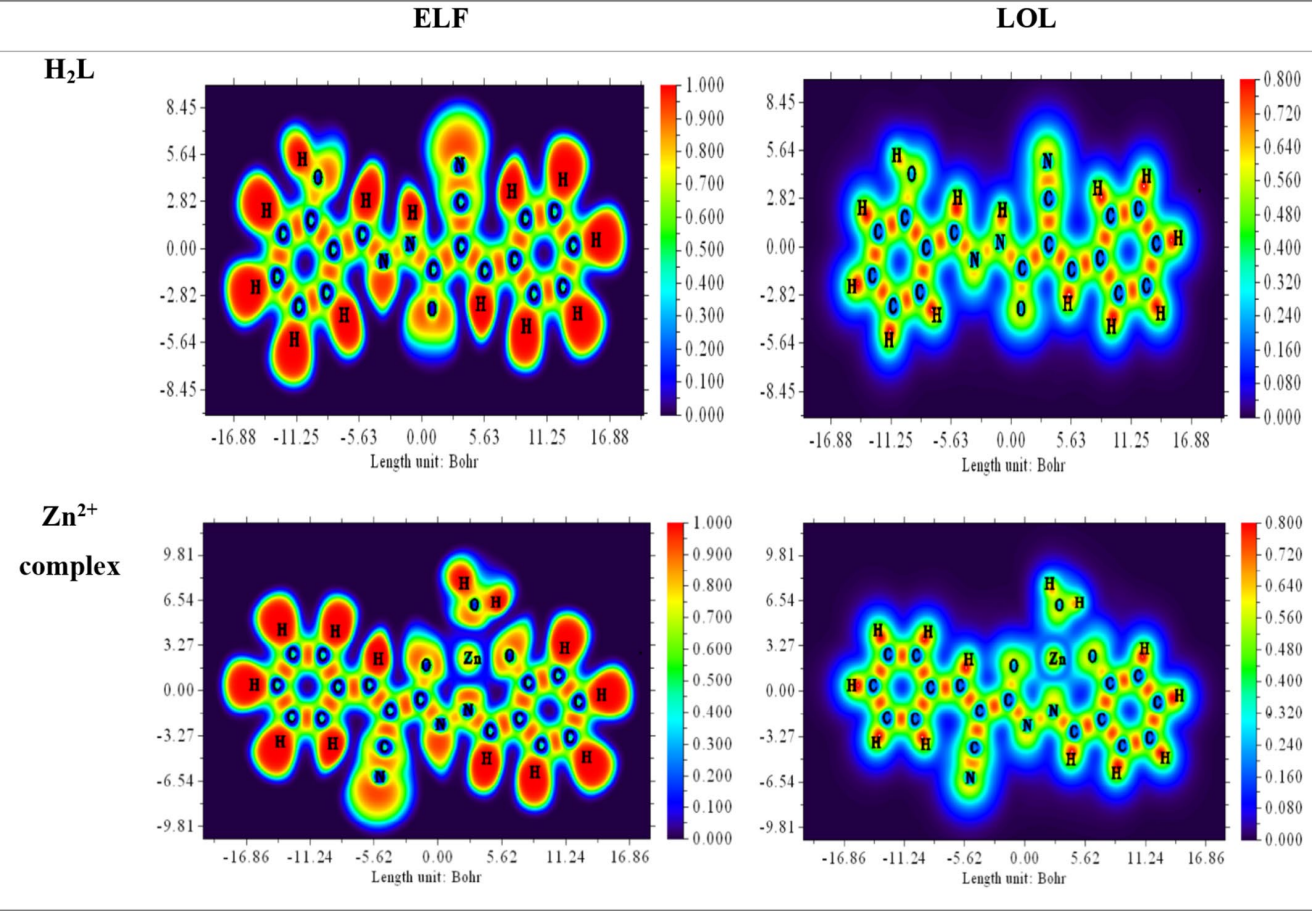
ELF and LOL iso-surfaces of the H_2_L and its Zn^2+^ complex.

To demonstrate the electron localization across the molecule, the Electron Localization Function (ELF) and Localization Orbital Locator (LOL) researches had been conducted. These functions provide information on bonding configurations, lone pairs, and core electron areas by depicting an electron pair and localization diagram^47,48^. Both functions had been calculated using the electron density resulting from the optimized shapes, that had been computed at the DFT/B3LYP level alongside the basis set 6-311G (d, p). Using Multiwfn 3.4.1. ^48^, ELF and LOL had been calculated and associated 2D contour maps were created. To explain the compound’s electronic structure and bonding characteristics, ELF and LOL studies were conducted^49,50^. Lone pairs, covalent bonds, and core electron zones may all be clearly distinguished thanks to these functions, which give a quantitative assessment of where the electrons are paired. Important information on chemical bonds present, the configuration of free electrons, and the general stability and reactivity of the molecule may be obtained by chemical imaging of these localization domains^51^. The ELF and LOL isosurfaces of H_2_L and its Zn^2+^ complex are shown in Fig. 7 as a 2-D representation translated onto the XY plane (Z = 0) using a color scale ranging from blue to red. It illustrates how electrons are localized and delocalized in each compound section. The strong covalent nature of the H_2_L and its M^2+^ complexes is shown by the detection of high ELF/LOL areas (red/yellow regions in Fig. 7) in C–H, C–C, and N–H bonds. The significant negative-potential regions found in the MEP are definitely correlated with lone pairs of electrons on N and O atoms, according to the ELF study. The core electron of the metals inside the complexes is also visible in the ELF/LOL patterns, which aid in the HOMO analysis of complexes. This congruence between the localization and electrostatic studies provides a thorough understanding of the electronic environment: electron-rich regions influence the hydrogen bonding patterns shown in experiments in addition to driving local reactivity. Crucially, delocalization within the H_2_L conjugation system is shown by the ELF/LOL patterns, indicating the stability of the H_2_L and its coordination compounds *via* conjugation. The orbital distribution seen in FMO plots and E_H−L_ is explained by this delocalization as well as the unique localization close to donor atoms. Combining MEP, ELF, and LOL data gives an explanation for how electronic characteristics impact molecular stability and potential reactivity, going beyond a visual representation of computational figures. Fig. S22 shows the ELF/LOL patterns of the Cu^2+^, Co^2+^, and Ni^2+^ complexes.

### Natural bond orbital (NBO) calculations

The main uses of NPA and NBO are to assess the strength and properties of charge delocalization as well as to estimate the molecule natural charge and interactions between the metal atoms and the ligand. Variations in basis sets have little effect on the NPA-NBO technique, which is the best strategy for atomic charge computations^44,52^. The inclusion of oxygen and nitrogen as ligand coordinating atoms offers a chance to improve the study of electron donation affinity. Table S9 displays the metal ions and the natural charges of the atoms in the coordinated ligands. In H_2_L, the net natural charge of the oxygen atoms O12 and O21 equals − 0.58117 and − 0.6882 e, respectively. In addition, Three nitrogen atoms, N11, N13, and N22, each have a net natural charge of -0.4337, -0.21823, and − 0.31097 e, respectively. These oxygen atoms have a higher net natural charge than these nitrogen atoms. This means that oxygen atoms are more likely to pair up with metal cations. In metal complexes, the metal cations have net charges of 1.31787, 0.81279, 1.05528, and 0.6726 e for Ni^2+^, Cu^2+^, Co^2+^, and Zn^2+^, respectively. The metal cations have net charges lower than (+ 2) because of the electrons transferred from ligand donating atoms to the metal ion, showing that the charges of the metal cation are greatly reduced through the negative electron density that is transmitted from the ligand units. Because the transferred charges from ligand to metal are larger than the back donation, the investigated complexes may be categorized as LMCT complexes.

The interaction involving “filled” (the donor) Lewis-type NBOs and “unfilled” (the acceptor) non-Lewis NBOs is documented in (Table S10). In the ligand, the most significant interactions happen between O12 lone pair (1) as a donor orbital and (σ^*^) C20 - H34 as an acceptor orbital, O12 lone pair (1) as a donor orbital and (σ^*^) N13 - C14 as an acceptor orbital and N11 lone pair (1) as a donor orbital and (π^*^) N13 - C14 as an acceptor orbital with stabilization energies of 386.94, 281.09, and 148.31 kcal/mol, respectively. These interactions contribute to stabilizing the ligand molecule. In addition to that, the charge transfer happens at O12^…^. C7-H28 with energy 6.49 kcal/mol, causing the deviation of the bond angle from the standard 120° in the optimized DFT structure of H_2_L. There is no bond (a centered electron pair between two atoms) between O/N atoms that are interacting with M²⁺ cations in the NBO study of the metal complexes system. Noting that the delocalization of electrons between the unoccupied anti-bonding orbitals of the metal cations LP^*^ and the donating atoms’ lone pair-filled orbitals LP(O/N) is the source of the M–O/N interactions. In metal complexes, multiple charge transfers occur for the ligand donating atoms (N13, O12, O21) and the water oxygen atom with vacant anti-bonding orbitals of the metal cations LP^*^ with significant stabilization energies. For instance, the net stabilization energies of charge transfer of LP donor orbitals of N13, O12, O21, O35, O38, and O40 to LP^*^ acceptor orbitals of Ni29 equal 135.4, 100.76, 104.44, 40.08, 64.97, and 128.01 kcal/mol, respectively. These interactions cause the stabilization of Ni^2+^ complexes along with strong hyperconjugative interactions LP(1) O12 → σ^*^ C7–H28, LP(2) O12 → σ^*^ C7–H28, and LP(2) O12 → σ^*^ O40–H43 (hydrogen bonding O12^…^.H43–O40) with net stabilization energy 411.77, 160.28, and 80.24 kcal/mol, respectively, as shown in Table S4. In the Cu^2+^ complex, the NBO analysis shows charge transfer LP donor orbitals of N13, O12, O21, and O35 to LP^*^ acceptor orbitals of Cu29 equal 37.30, 75.58, 51, and 67.3 kcal/mol, respectively, besides the strong hyperconjugative interactions LP(2) O12 → σ^*^ C16–O21 and LP(1) O35 → σ^*^ C19–H33 with net stabilization energy of 187.21 and 166.74 kcal/mol, respectively. The Co^2+^ complex shows significant hyperconjugative interactions with high stabilization energies, where LP(1) O21 → σ^*^ O38–H39, LP(2) O38 → σ^*^ O38–H39, LP(2) O21 → σ^*^ O38–H39, LP(1) O21 → σ^*^ C14–H30, and LP(2) O35 → σ^*^ C14–H30 have very significant stabilization energies of 974.03, 518.14, 295.34, 190.49, and 160.82 kcal/mol, respectively. In addition, the charge transfer from LP donor orbitals of O12, O21, O35, O38, and O60 to LP^*^ acceptor orbitals of Co29 equals 7.45, 75.58, 118.25, 30.85, and 7.86 kcal/mol, respectively. Finally, the NBO analysis of Zn^2+^ complex shows charge transfer of LP donor orbitals of N13, N22, O12, O21, and O35 to LP^*^ acceptor orbitals of Zn29 equals 55.59, 20.29, 63.7, 72.51, and 53.37 kcal/mol, respectively, besides the strong hyperconjugative interactions LP(1) O35 → σ^*^ C19–H30 and LP(2) O35 → σ^*^ C10–N22 with stabilization energies of 166.74 and 101.13 kcal/mol, respectively.

### NLO properties

Due to their exceptional structural and electrical characteristics, Schiff bases have garnered a lot of interest in NLO investigations of materials, making them a good fit for applications in optoelectronics. One important feature that describes a molecule’s or crystalline structure’s first order nonlinear optical activity is hyperpolarizability (β). This property determines how extent a material can transmit an input optical field to a newly frequency. Numerous internal and extrinsic factors, like the structure of the molecule, charge distribution, and external factors such as applied electric fields, can affect the hyperpolarizability of the H_2_L and its M^2+^ complexes. An induced dipole moment, polarizability (α), and hyperpolarizability (β) of the system are typical indicators of a molecule’s polarization in reaction to an electric field^53^. The following is a description of the key parameters^54^:

Dipole moment,

$$\:{\upmu\:}=\sqrt{{{\upmu\:}\mathrm{x}}^{2}+{{\upmu\:}\mathrm{y}}^{2}+{{\upmu\:}\mathrm{z}}^{2}}$$

Polarizability,

$$\:\:=\frac{1}{3\:}(\mathrm{x}\mathrm{x}\hspace{0.17em}+\hspace{0.17em}\mathrm{y}\mathrm{y}\hspace{0.17em}+\hspace{0.17em}\mathrm{z}\mathrm{z})2$$

Anisotropy of the polarizability,

$$\:{\Delta\:}{\upalpha\:}\:=\sqrt{\frac{{({\upalpha\:}\mathrm{x}\mathrm{x}-{\upalpha\:}\mathrm{y}\mathrm{y})}^{2}+{({\upalpha\:}\mathrm{y}\mathrm{y}-{\upalpha\:}\mathrm{z}\mathrm{z})}^{2}+{({\upalpha\:}\mathrm{z}\mathrm{z}-{\upalpha\:}\mathrm{x}\mathrm{x})}^{2}+6{{\upalpha\:}\mathrm{x}\mathrm{y}}^{2}+6{{\upalpha\:}\mathrm{z}\mathrm{y}}^{2}+{6{\upalpha\:}\mathrm{x}\mathrm{z}}^{2}}{2}}$$

First hyperpolarizability,

$$\:{\upbeta\:}=\sqrt{{{\upbeta\:}\mathrm{x}}^{2}+{{\upbeta\:}\mathrm{y}}^{2}+{{\upbeta\:}\mathrm{z}}^{2}}$$

Where,

$$\:{{\upbeta\:}\mathrm{x}}^{2}=\:({\upbeta\:}\mathrm{x}\mathrm{x}\mathrm{x}\hspace{0.17em}+\hspace{0.17em}{\upbeta\:}\mathrm{x}\mathrm{y}\mathrm{y}\hspace{0.17em}+\hspace{0.17em}{\upbeta\:}\mathrm{x}\mathrm{z}\mathrm{z})2$$

$$\:{{\upbeta\:}\mathrm{y}}^{2}=\:({\upbeta\:}\mathrm{y}\mathrm{x}\mathrm{x}\hspace{0.17em}+\hspace{0.17em}{\upbeta\:}\mathrm{y}\mathrm{y}\mathrm{y}\hspace{0.17em}+\hspace{0.17em}{\upbeta\:}\mathrm{y}\mathrm{z}\mathrm{z})2$$

$$\:{{\upbeta\:}\mathrm{z}}^{2\:}=\:({\upbeta\:}\mathrm{z}\mathrm{x}\mathrm{x}\hspace{0.17em}+\hspace{0.17em}{\upbeta\:}\mathrm{z}\mathrm{y}\mathrm{y}\hspace{0.17em}+\hspace{0.17em}{\upbeta\:}\mathrm{z}\mathrm{z}\mathrm{z})2$$

After converting the calculated (α) and (β) from atomic units (a.u.) to electrostatic units (esu), urea was used as a reference nonlinear optical material (dipole moment = 1.3732 Debye and first order hyperpolarizability = 3.7389 × 10^− 31^ esu)^55^. Effective intermolecular interactions will be present in a molecule with a high (µ). The results (Table S11) show that Ni^2+^ complex has the greatest value at 9.06 Debye, followed by Zn^2+^, Cu^2+^, and H_2_L, respectively, while the Co^2+^ complex has a lesser dipole moment than the other compounds, measuring 1.29 Debye. The Cu^2+^ complex had the lowest calculated first-order hyperpolarizability (βtotal) of 7.971 × 10^− 30^ esu, while Ni^2+^ complex has the highest value of 6.305 × 10^− 29^ esu. These values were followed by H_2_L, Co^2+^, and Zn^2+^. Every chemical under investigation had values greater than urea. An increase in the charge transfer (CT) interactions inside the molecule is linked to this rise in hyperpolarizability. NLO characteristics are significantly impacted by the delocalization of π-electrons across the conjugated system and the presence of functional groups with electron-donating and electron-withdrawing actions^55^. The basis for next studies concentrating on chemical changes to optimize NLO responses is established by these results. The molecular symmetry of the compounds is another crucial factor controlling their hyperpolarizability. The compounds’ non-centrosymmetric crystal stacking permits significant SHG activity, but centrosymmetric structures often decrease NLO property due to symmetry constraints. The lattice’s nonlinear optical response is further enhanced by charge delocalization made possible by E_H−L_, hydrogen bonding, and dipole-dipole interactions.

### Molecular docking

**Fig. 8:**
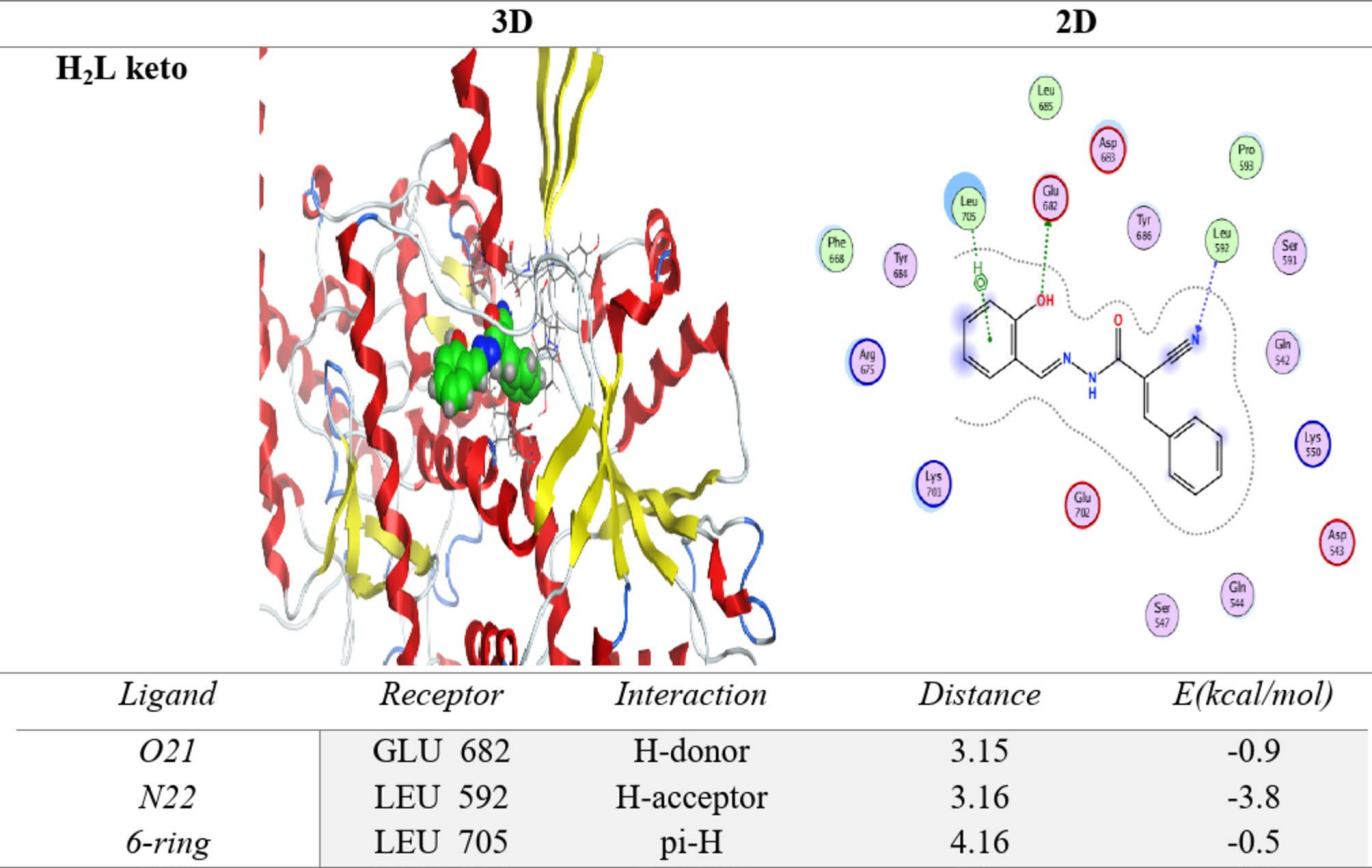
2D and 3D Docking interaction between H_2_L keto with (PDB code = 4fm9).

**Fig. 9:**
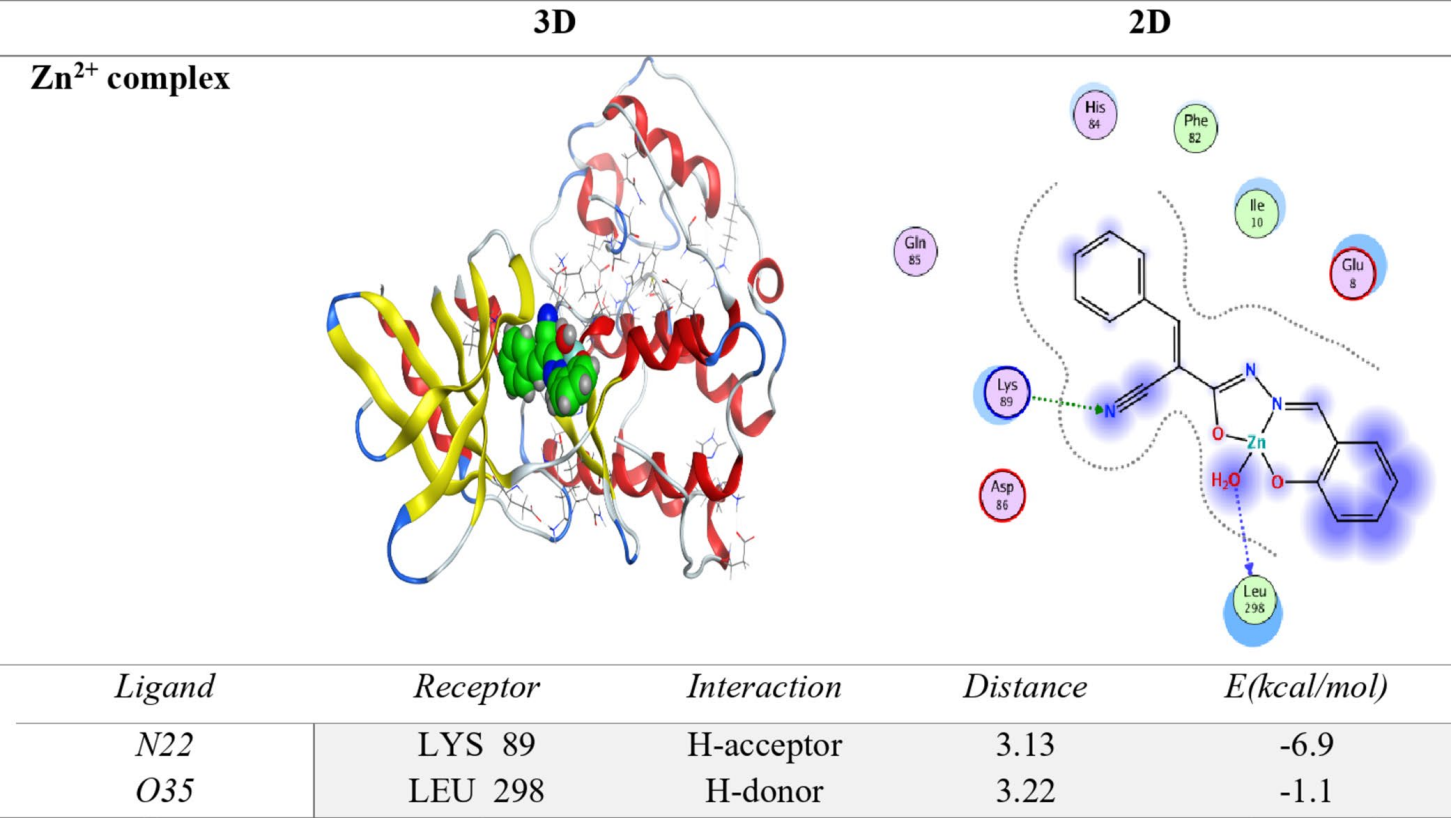
2D and 3D Docking interaction between Zn^2+^ complex with (PDB code = 3ig7).

It is a useful method for predicting the likely orientation of ligands and receptors. The affinity of the H_2_L and its M^2+^ complexes for binding to the receptors determines their effectiveness. A fundamental cyclin-dependent kinase (Cdk5) has been thoroughly described for its function in the neurological system of the body^51^. H_2_L (keto and enol forms) and its complexes’ interactions with the two targeted proteins had been simulated in two and three dimensions. The molecular interactions and binding energy of each chemical were examined after docking. Before doing molecular docking, the docking technique and methodology must be adhered to. The binding energies of the H_2_L and its complexes, the Docking Score (S), and the Root Mean Square Deviation (RMSD) were used to examine the final data (Table 1). According to the docking data, the Zn^2+^ complex is the most successfully docked molecule with the colon cancer protein (3ig7), displaying docking scores of -6.63, whereas H_2_L keto is the most successfully docked molecule with the liver cancer protein (4fm9), displaying docking scores of -6.85. The molecular docking of (2-cyano-N’-2-hydroxybenzylidene)-3-phenylacrylohydrazide in the keto form with the 4fm9 protein is described in (Fig. 8). H-donor, H-acceptor, and pi-H interactions are how the ligand interacts with GLU 682, LEU 592, and LEU 705 in 4fm9 protein (receptor). Due to its interaction as an H-donor at a bond length of 3.15 Å and − 0.9 kcal/mol, the free hydroxyl group indicates the highest S of the ligand with the 4fm9 protein. H_2_L interacts with 3ig7 protein through two hydrogen bonds with GLU 8 and LEU 83 at bond lengths 2.97 and 3.19 Å and binging energies − 1.6 and − 2.6 kcal/mol, respectively. (Fig. 9) shows Zn^2+^ complex’s molecular docking with 3ig7. In comparison to H_2_L and its other M^2+^ complexes, the Zn^2+^ complex got the greatest docking score. The Zn^2+^ complex interacts with LYS 89 and LEU 298 of the 3ig7 cancer protein (receptor) *via* hydrogen bonds at 3.13 and 3.22 Å, with binding energies equal − 6.9 and − 1.1 kcal/mol, respectively. Zn^2+^ complex interacts with 4fm9 through hydrogen bond with LYS 701 at 3.29 Å bond length and − 0.5 kcal/mol binding energy. It also forms pi-H and pi-cation (bond lengths = 4.00, 4.16 Å, binding energies= -0.5, -0.8 kcal/mol, respectively) with LEU 592 and ARG 675, respectively.

The results (Table S12) show the binding modes of H_2_L and its coordination compounds towards 3ig7 and 4fm9 proteins. Ni^2+^ complex binds to 4fm9 protein through hydrogen bond (bond length = 3.17 Å, binding energy = -1.8 kcal/mol) and pi-H (bond length = 4.04 Å, binding energy = -0.5 kcal/mol) interactions with LEU 685 and TYR 684, respectively. Its interaction with 3ig7 is through a hydrogen bond with GLN 131 (bond length = 2.62 Å, binding energy = -1.5 kcal/mol). Cu^2+^ complex binds to 4fm9 protein through hydrogen bonds (bond lengths = 2.78, 3.25 Å, binding energy = -2.6, -3.0 kcal/mol) with ASP 683 and GLU 682, respectively and pi-H (bond lengths = 4.39 Å, binding energy = -0.5 kcal/mol) interaction with LYS 701. Its interaction with 3ig7 is through a hydrogen bond with LYS 89 (bond lengths = 2.87 Å, binding energy = -0.5 kcal/mol). Co^2+^ complex binds to 4fm9 protein through three hydrogen bonds (bond lengths = 3.29, 2.51, 2.81 Å, binding energy = -1.3, -1.4, -2.5 kcal/mol) with ASP 683, ASP 683 and LYS 701, respectively (ASP 683 forms two hydrogen bonds with C7 and O35 of the complex). It also forms pi-H (bond length = 3.64 Å, binding energy = -1.8 kcal/mol) interaction with LEU 685. Its interactions with 3ig7 are four hydrogen bonds (bond lengths = 2.67, 2.57, 3.32, 3.00 Å, binding energies = -1.1, -0.5, -0.9, -2.1 kcal/mol) with GLU 12, GLN 131, ASN 132 and LYS 89, respectively.

**Table 1:** Molecular docking of the compounds with (PDB code = 4fm9) of liver cancer protein and (PDB code = 3ig7) of colon cancer protein.

Compound	4fm9		3ig7	
	S	RMSD	S	RMSD
H_2_L keto	-6.850	2.428	-6.283	1.800
H_2_L enol	-6.800	2.544	-6.270	2.575
Ni^2+^ complex	-6.315	1.558	-6.395	1.149
Cu^2+^ complex	-5.027	1.269	-4.979	1.153
Co^2+^ complex	-5.782	2.423	-5.894	3.239
Zn^2+^ complex	-6.260	2.92	-6.626	6.369

### Cytotoxicity activity

**Fig. 10:**
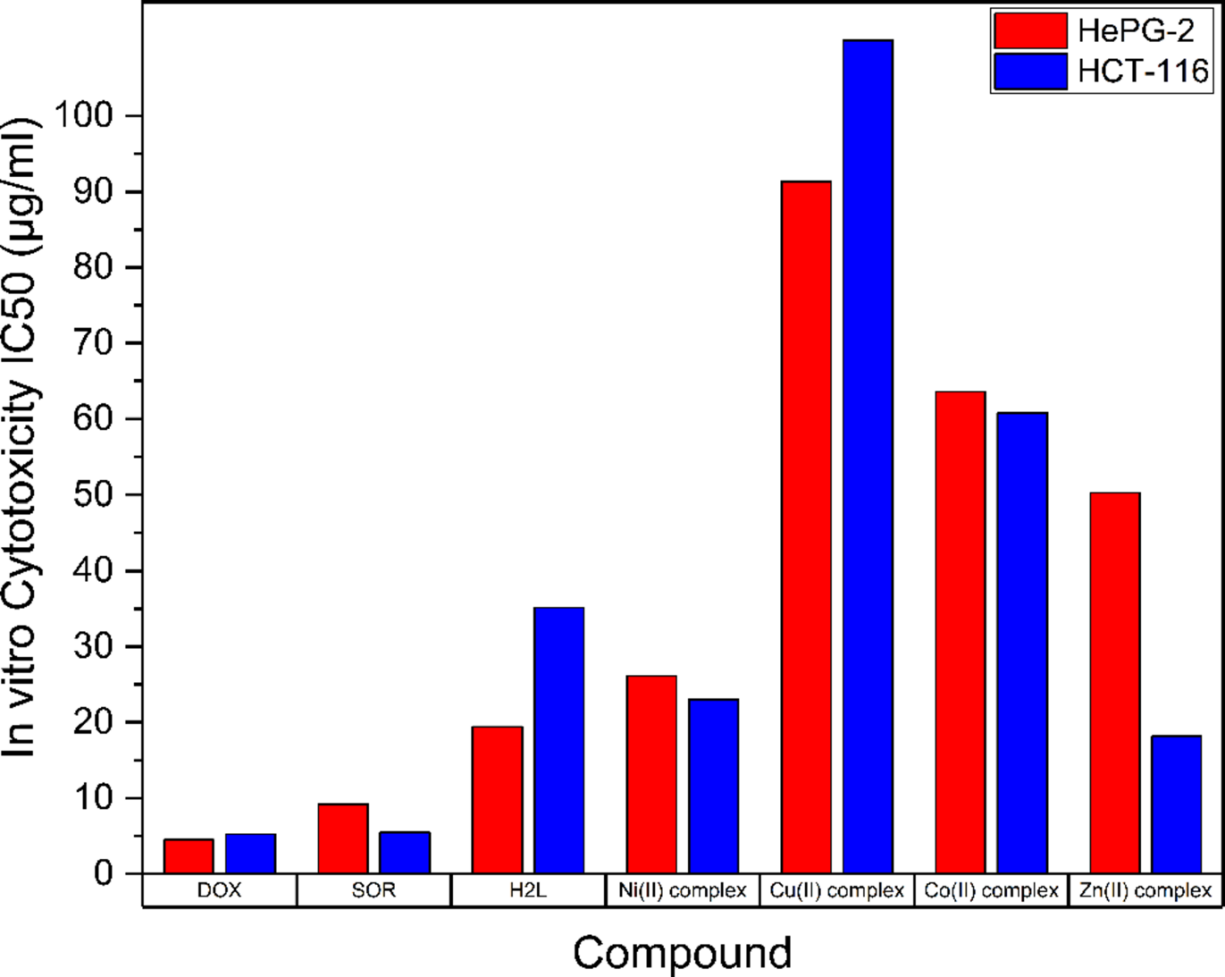
IC_50_ of the H_2_L and M^2+^ complexes against HePG2 and HCT-116 incorporating DOX and SOR as standards.

The in vitro cytotoxic effects of the compounds against HePG2 and HCT-116 cell lines are presented (Fig. 10). H_2_L shows a strong activity with an IC_50_ of 19.42 ± 1.4 µg/ml against the HePG2 cell line and a moderate effect with an IC_50_ of 35.14 ± 2.2 µg/ml against the HCT-116 cell line. The free hydroxyl and nitrile groups allowed H_2_L to interact with the receptor *via* H-bonding, as the docking data showed. This could be the reason why the free ligand is more active. The Zn^2+^ complex has moderate action against the HePG2 cell line (IC_50_ 50.26 ± 2.9 µg/ml), but strong activity against the HCT-116 cell line (IC_50_ 18.16 ± 1.4 µg/ml). Additionally, Ni^2+^ complex showed modest action against both cells. Cu^2+^ complexes had no cytotoxic impact on the HCT-116 cell line and a bit weak action against the HePG2 cell line. H_2_L had the lowest viability against the HePG2 cell line (Fig. S23). Conversely, the Zn^2+^ complex had the lowest vitality against the HCT-116 cell line.

The results are compared with the previously reported synthesized compounds’ anticancer activity against the same cell lines^1^. H_2_L and its coordination compounds showed lower anticancer activity than the previously reported synthesized compounds. The ligand previously reported by Hosny (2023)^1^ showed a higher activity against HePG2 cell line (IC_50_ 8.53 ± 0.6 µg/ml) than H_2_L and a higher activity against HCT-116 cell line (IC_50_ 7.87 ± 0.5 µg/ml) than the Zn^2+^ complex. From the molecular docking, the lower activity of H_2_L and its coordination compounds may be a result of the lower activity of the nitrile group (C ≡ N) as compared to the free thiocarbonyl group (C = S) of the previously reported synthesized compounds.

## Conclusion

In summary, structural characterization verified that a new ligand **2-cyano-N’-(2-**hydroxybenzylidene)-3-phenylacrylohydrazide and some of its metal chelates have been successfully synthesized. The ligand has different modes of chelations according to the metal ion used. It coordinates Ni^2+^, Cu^2+^ and Zn^2+^ in a tridentate manner forming 1:1 chelates. On the other hand it acts in a bidentate mode with Co^2+^ forming a 1:2 metal chelate.

DFT simulation of the spectra of the compound provided good theoretical information about the stereochemistry and mode of chelation.

The optical band gap measurements demonstrate semi-conducting nature of the ligand and its metal chelate. This work demonstrates that the free ligand and some of its metal chelate especially Zn^2+^ complex have strong cytotoxicity against HePG2 and HCT-116, respectively. The variation of the activity of the metal chelates highlights the effect of the metal ion. Although further investigations to study the cytotoxicity in vivo and the safety of studied compound on long term used are still necessary.

## Supplementary Information

Below is the link to the electronic supplementary material.

Supplementary Material 1

## Data Availability

All data supporting the findings of this study are available within the paper and its Supplementary Information.
