# Supplementary material for: Synthesis, spectral, nonlinear optical properties, molecular docking and cytotoxicity studies on some metal complexes derived from 2-Cyano-N’-(2-Hydroxybenzylidene)-3-Phenylacrylohydrazide
Source: Sci Rep. 2026 Jul 20;16:22706. doi: 10.1038/s41598-026-54876-4 (PMC13385901; doi:10.1038/s41598-026-54876-4)
Supplement: Supplementary file 1 — Supplementary Material 1 [file 41598_2026_54876_MOESM1_ESM.docx]

**Table S1.** Elemental analyses, color and melting point of 2-cyano-N'-(2-hydroxybenzylidene)-3-phenylacrylohydrazide (H_2_L) and its coordination compounds

| Compound (Formula; Mol. Wt.) | Color | M.P. (^°^C) | Elemental analyses % Found (Calculated) | | | |
| --- | --- | --- | --- | --- | --- | --- |
|  |  |  | **C** | **H** | **N** | **M** |
| 2-cyano-N'-(2-hydroxybenzylidene)-3-phenylacrylohydrazide (H_2_L)  (291.31) | yellow | 180 | 69.88  (70.09) | 4.09  (4.50) | 10.16  (10.98) | - |
| [Cu(L).H_2_O]2H_2_O  (406.88) | brown | 290 | 50.68  (50.2) | 3.87  (4.2) | 9.59  (10.3) | 15.70  (15.59) |
| [Zn(L).H_2_O]4H_2_O  (444.74) | yellow | >300 | 46.07  (45.9) | 4.02  (4.8) | 9.19  (9.40) | 14.61  (14.61) |
| [Co(HL)_2_.(H_2_O)_2_]$\frac{\boldsymbol{3}}{\boldsymbol{2}}$ H_2_O (702.59) | brown | >300 | 57.58  (58.07) | 4.06  (4.41) | 12.32  (11.96) | 9.20  (8.39) |
| [Ni(L).(H_2_O)_3_]3H_2_O  (456.07) | brown | >300 | 43.98  (44.80) | 4.96  (5.10) | 9.57  (9.20) | 12.31  (12.71) |


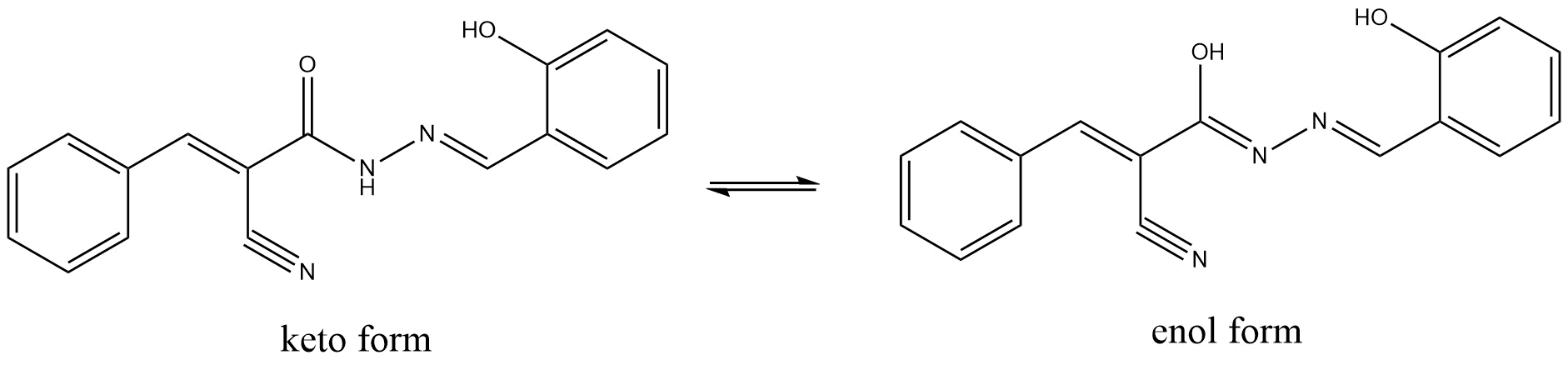


**Scheme S1.** The tautomeric forms of 2-cyano-N'-(2-hydroxybenzylidene)-3-phenylacrylohydrazide


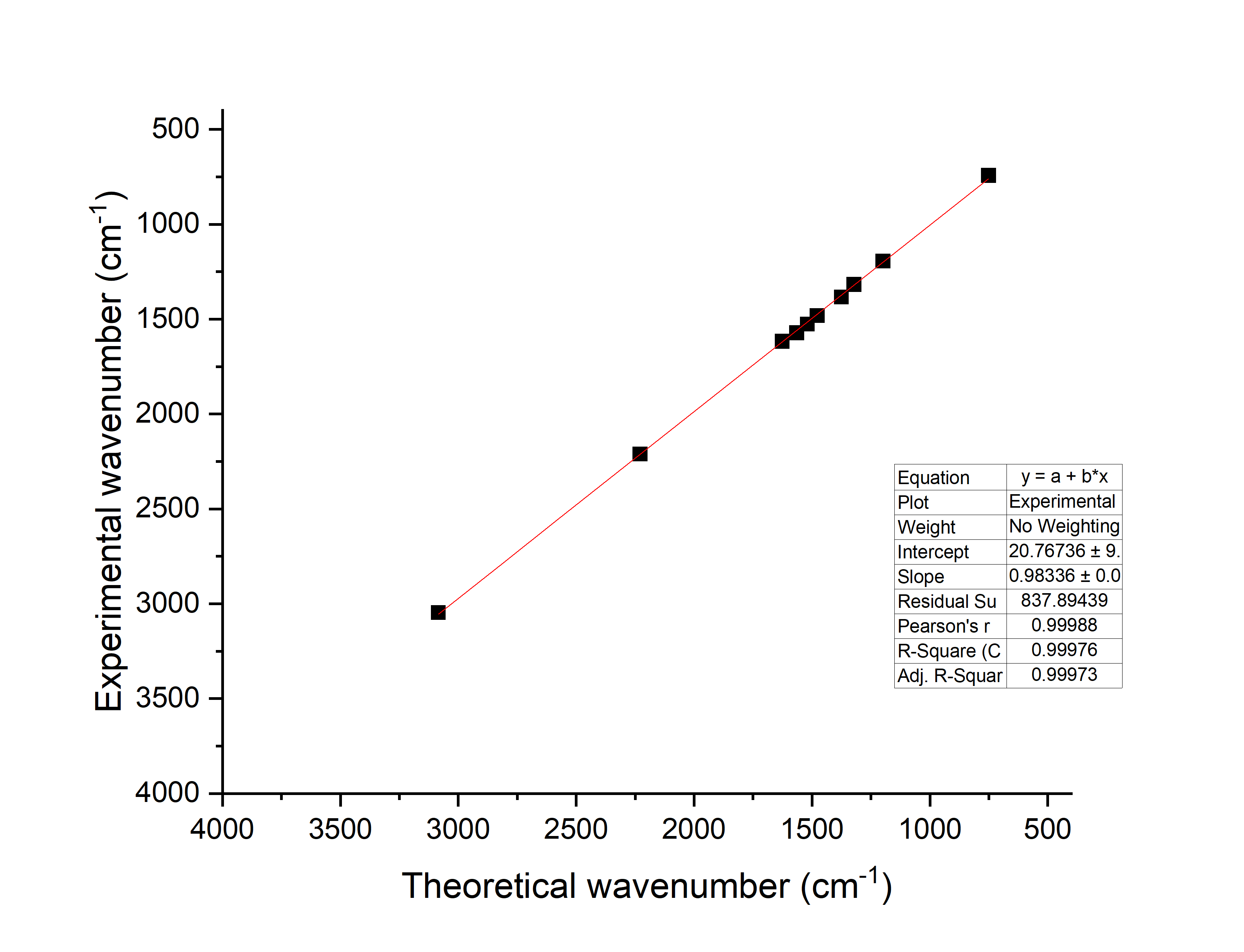


**Fig. S1.** The correlation graph of the experimental and theoretical vibrational IR of H_2_L


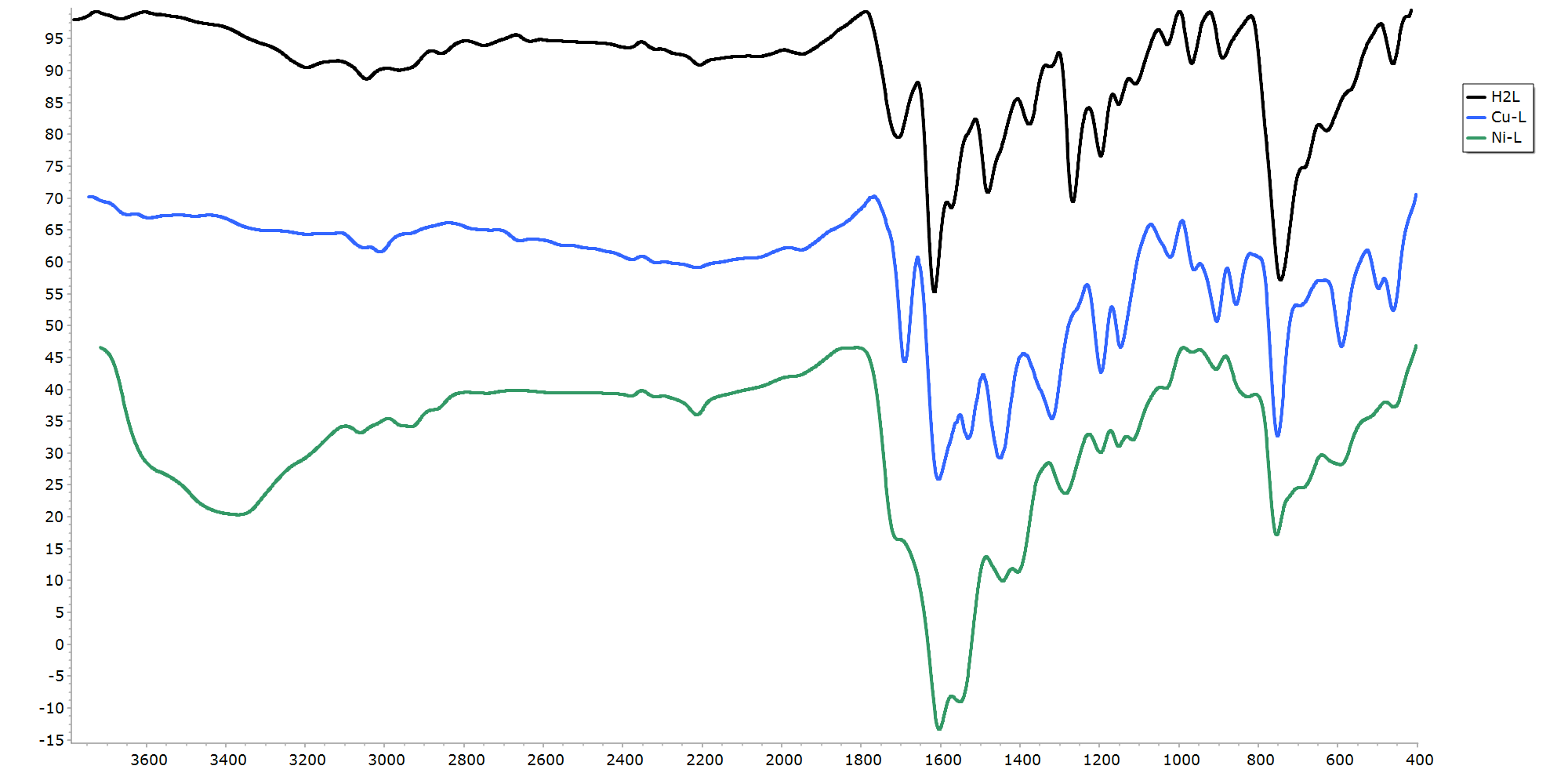


**Fig. S2.** The IR spectra of the ligand and its Ni(II) & Cu(II) complexes.


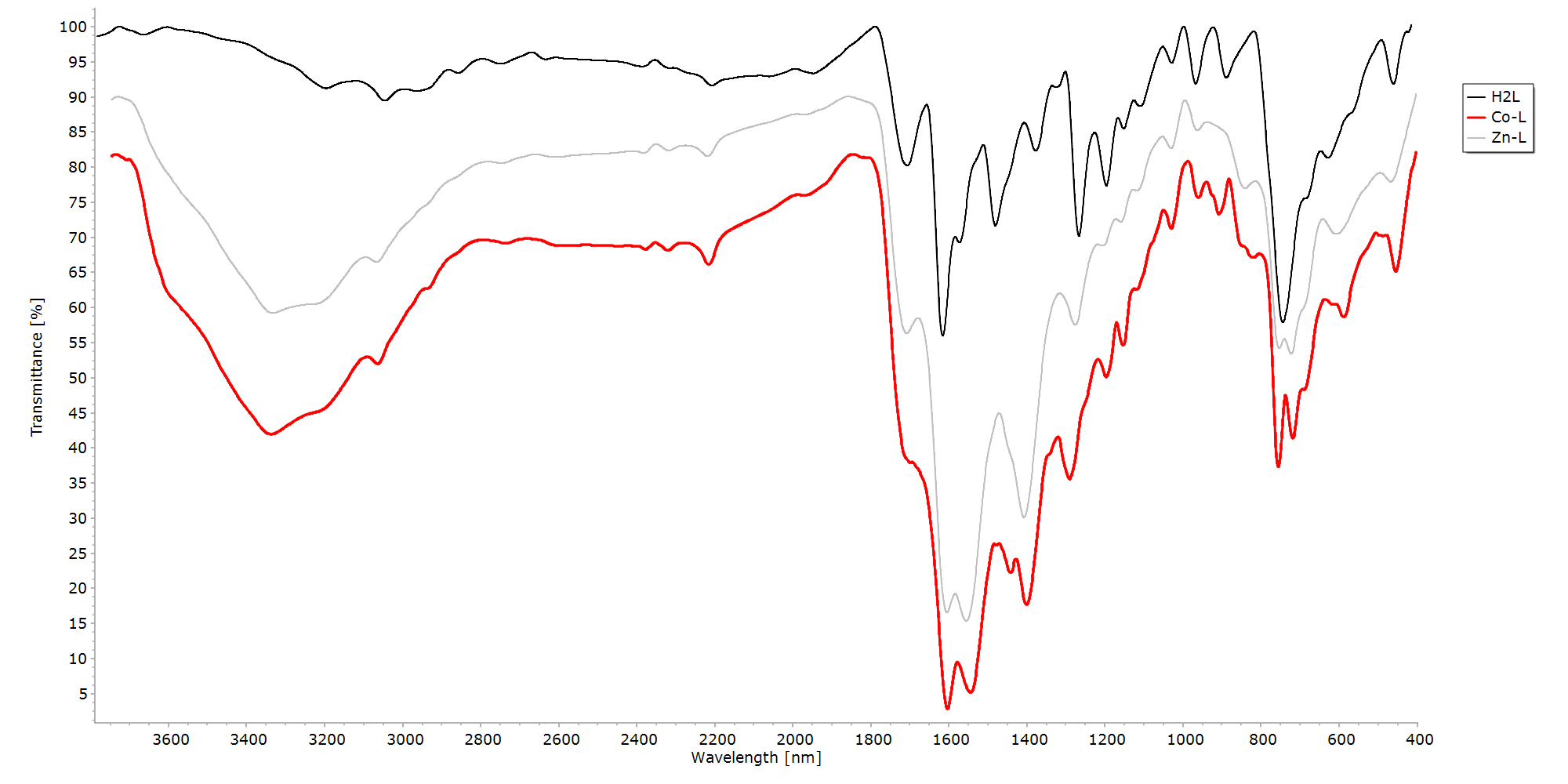


**Fig. S3.** The IR spectra of the ligand and its Co(II) & Zn(II) complexes.


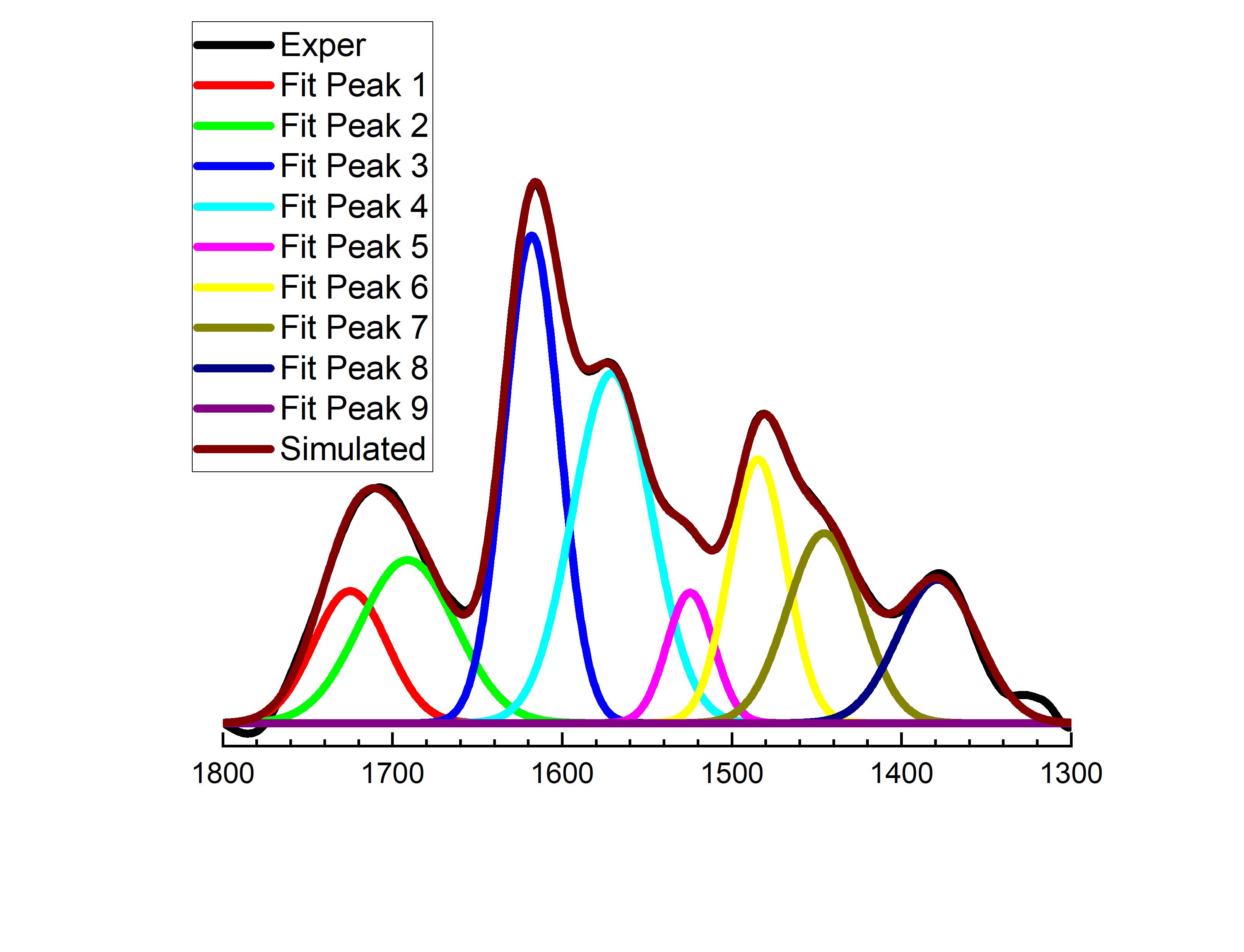

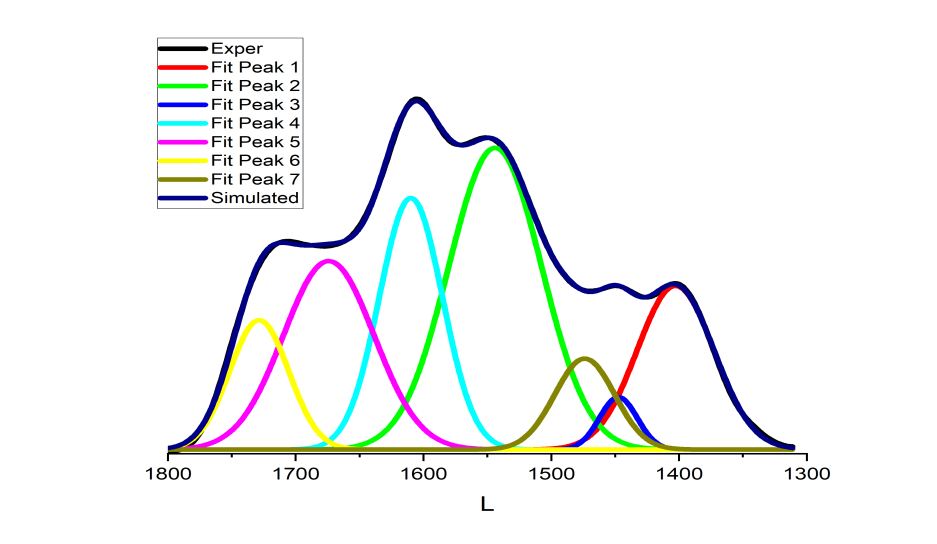


**Fig. S4.** Deconvolution analysis of H_2_L **Fig. S5.** Deconvolution analysis of Co(II) complex

**
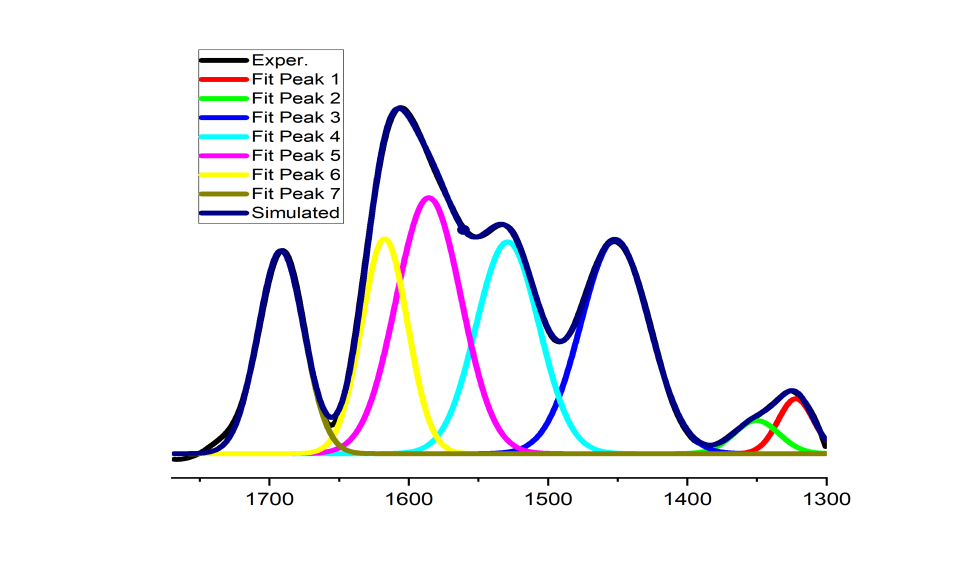
**
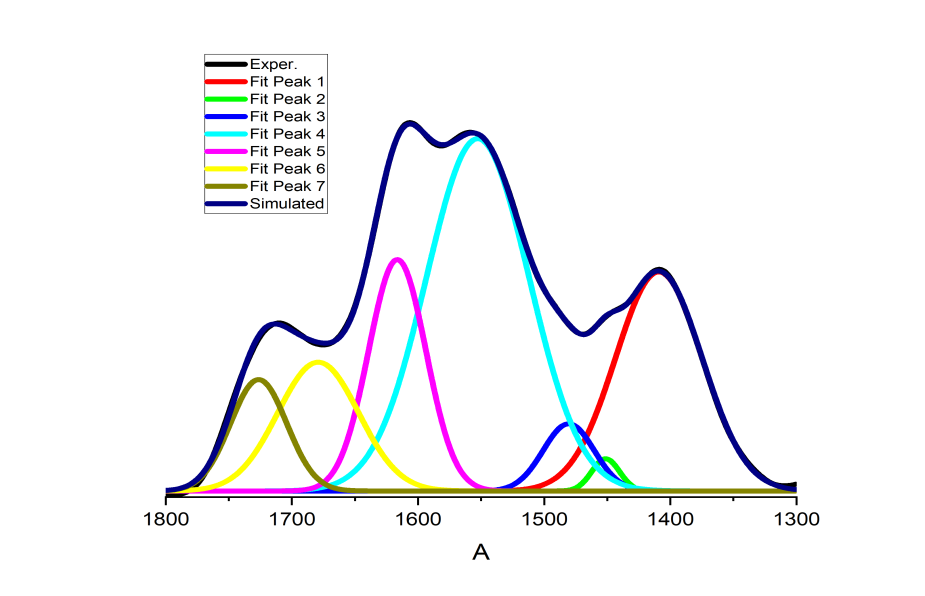


**Fig. S6.** Deconvolution analysis of Cu(II) complex **Fig. S7.** Deconvolution analysis of Ni(II) complex


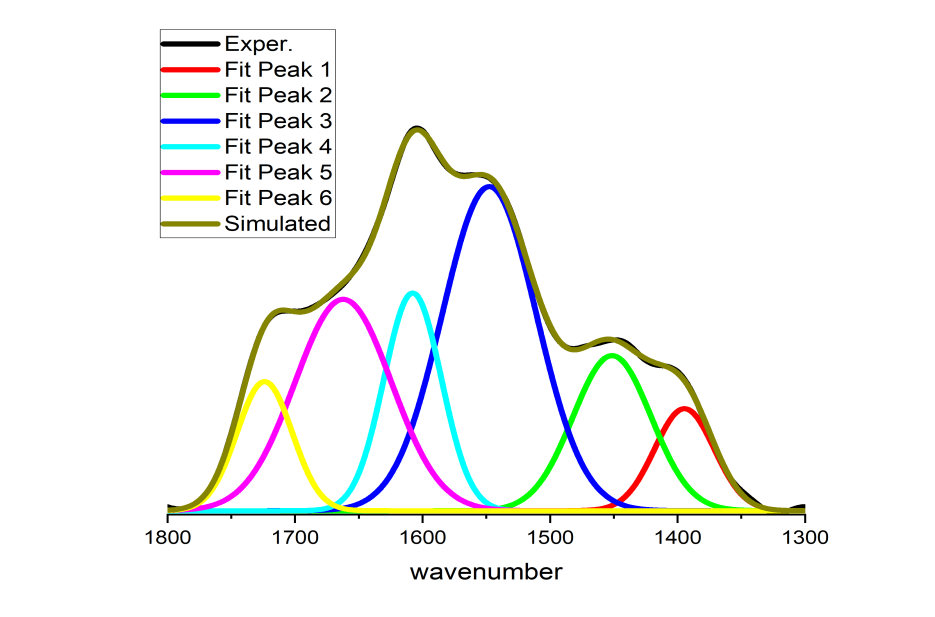


**Fig. S8.** Deconvolution analysis of Zn(II) complex

**Table S2.** Selected IR bands of the compounds

| Vibration | H_2_L | Complexes | | | |
| --- | --- | --- | --- | --- | --- |
|  |  | Ni^2+^ | Cu^2+^ | Co^2+^ | Zn^2+^ |
| ν(OH) | 3343 | - | - | 3347 | - |
| δ(OH) | 1377  686 | - | - | 1397  691 | - |
| ν(NH) | 3202 | - | - | - | - |
| δ(NH) | 1572 | - | - | - | - |
| ν(C-H)_sp2_ | 3049 | 3060 | 3057 | 3060 | 3062 |
| ν(C≡N) | 2211 | 2211 | 2214 | 2214 | 2214 |
| ν(CONH) | 1710^*^ | - | - | - | - |
| ν(C=O) | 1693^*^ | - | - | - | - |
| ν(C=N) | 1616^*^ | 1604^*^ | 1605^*^ | 1603^*^ | 1604^*^ |
| ν(C=N)^*^ | - | 1688^*^ | 1688^*^ | 1689^*^ | 1689^*^ |
| ν(N–N) | 1195 | 1196 | 1195 | 1198 | 1195 |
| ν(M-O) | - | 583 | 588 | 588 | 596 |
| ν(M-N) | - | 443 | 497 | 453 | 463 |

*The values obtained from the deconvolution analysis


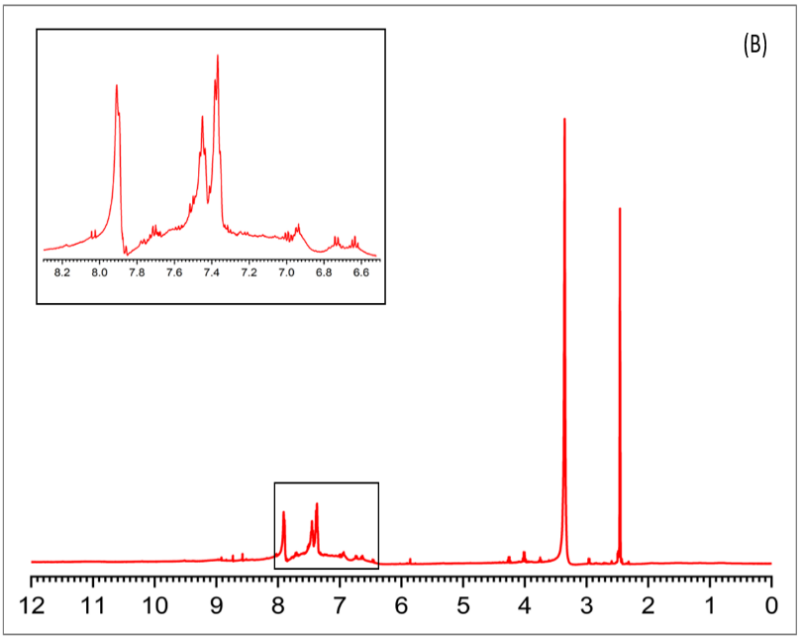

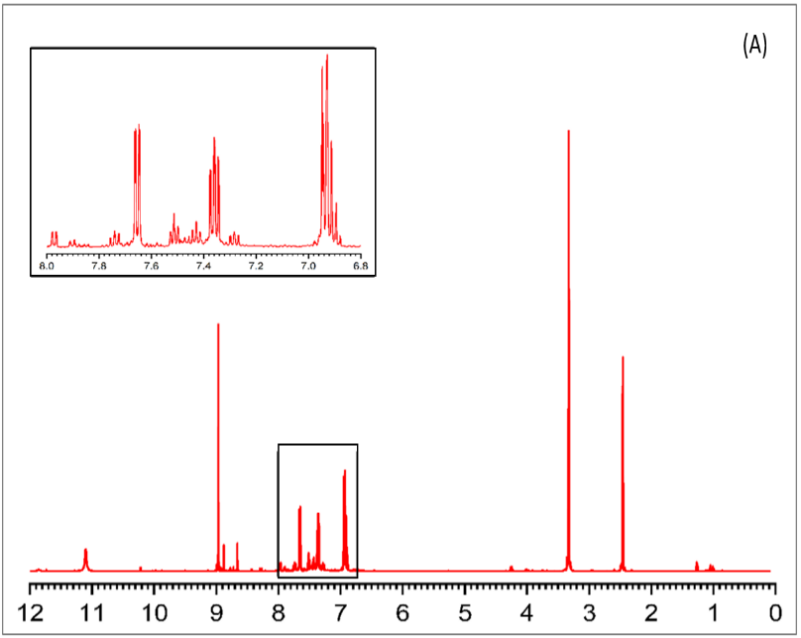


**Fig S9.** The ^1^H-NMR spectra of the H_2_L (A) and its Zn^2+^ complex (B).


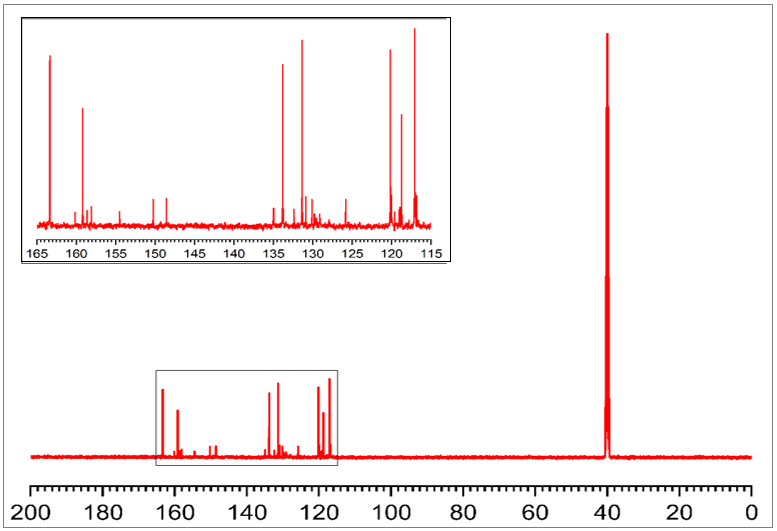


**Fig. S10.** ^13^C-NMR spectrum of the ligand H_2_L


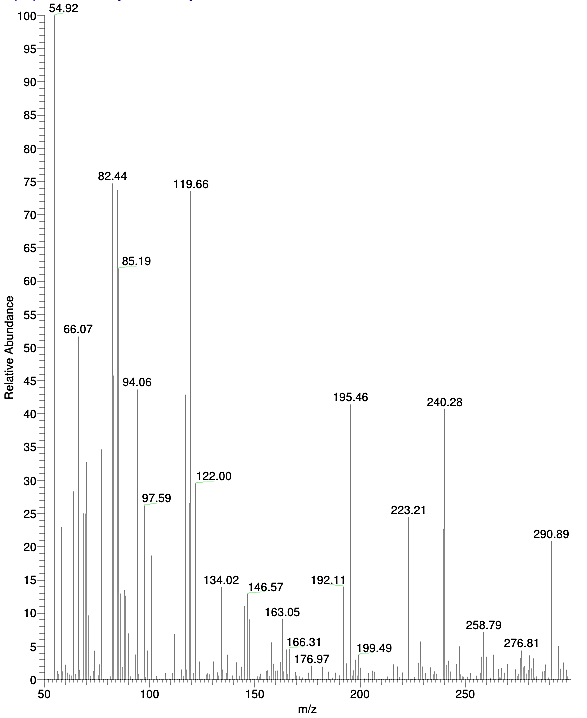


**Fig. S11.** Mass spectrum of H_2_L


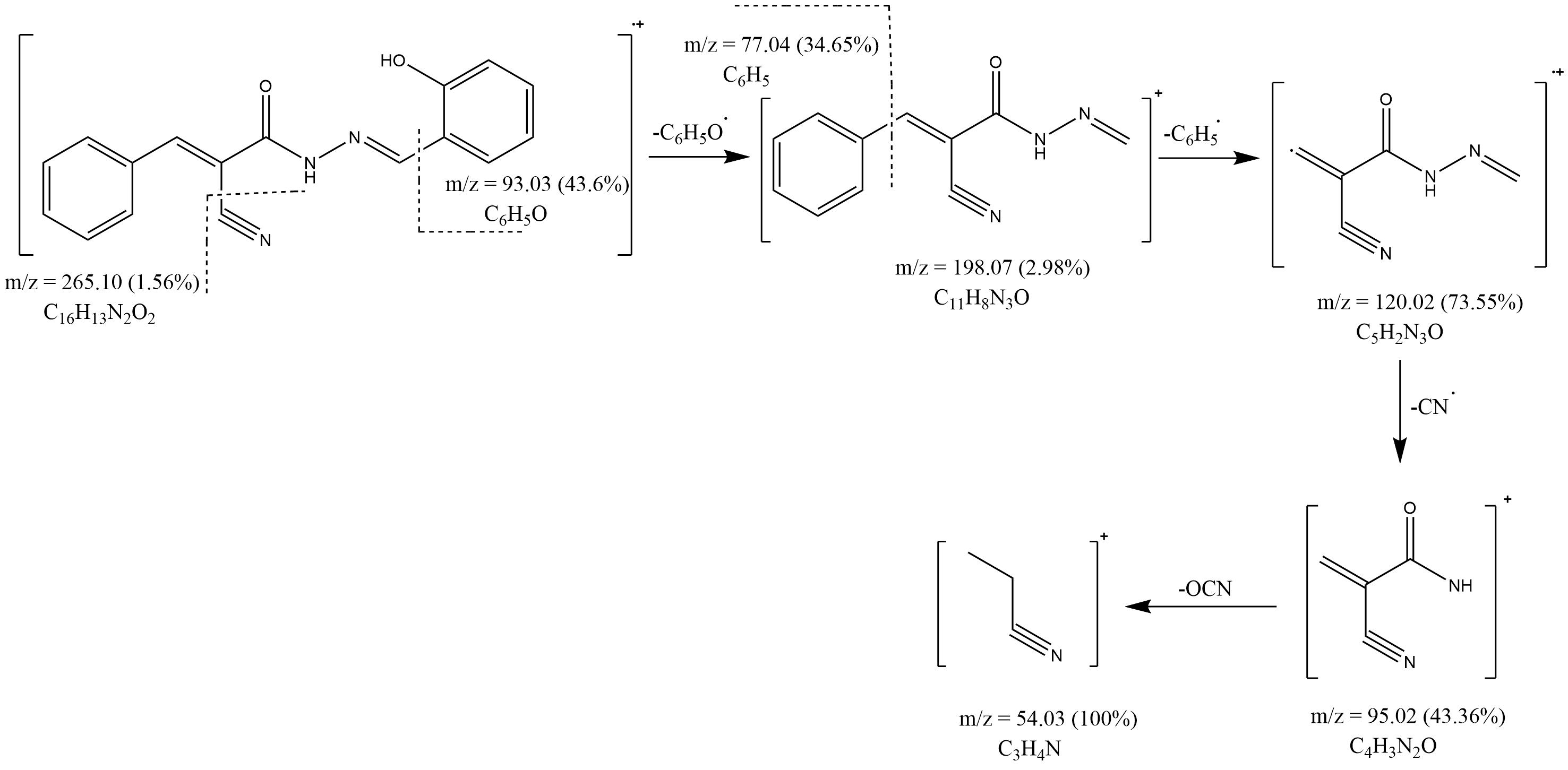


**Scheme S2.**


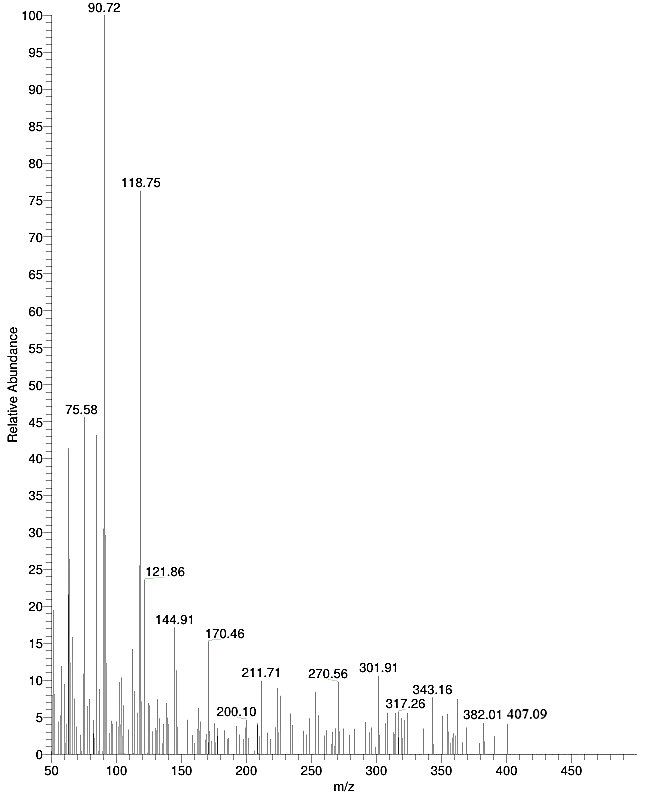


**Fig. S12.** Mass spectrum of [Cu(L).H_2_O]2H_2_O


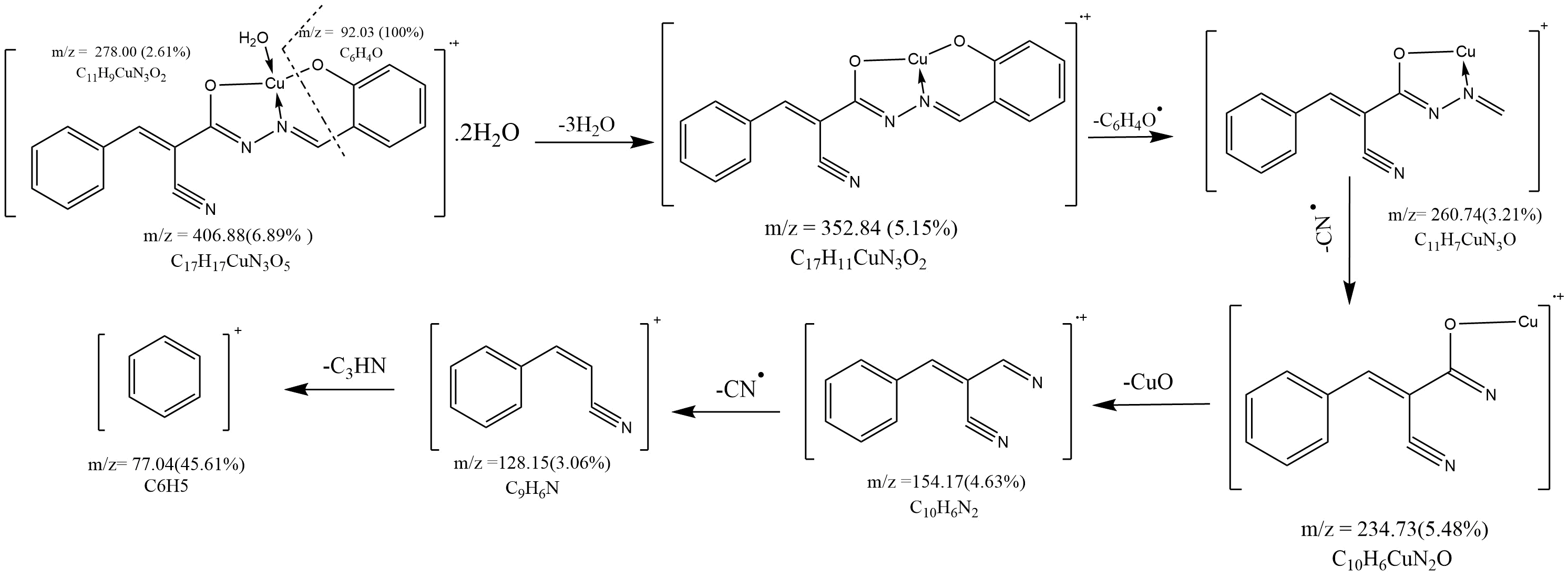


**Scheme S3.**


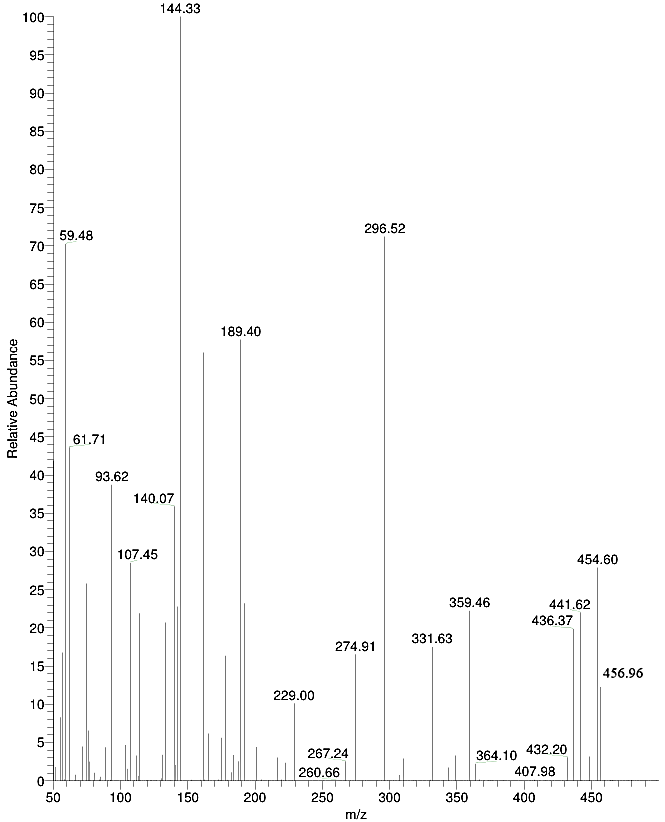


**Fig. S13.** Mass spectrum of [Ni(L).(H_2_O)_3_]3H_2_O


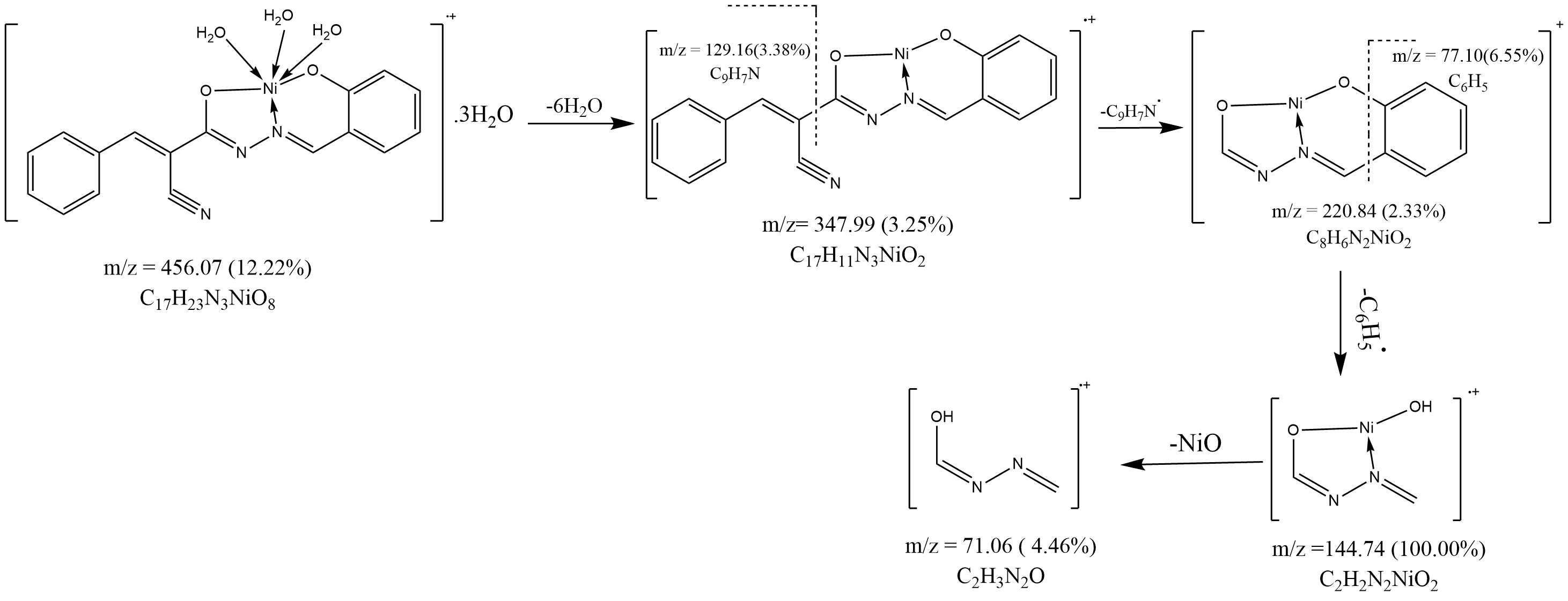


**Scheme S4.** Fragmentation pattern of [Ni(L).(H_2_O)_3_]3H_2_O


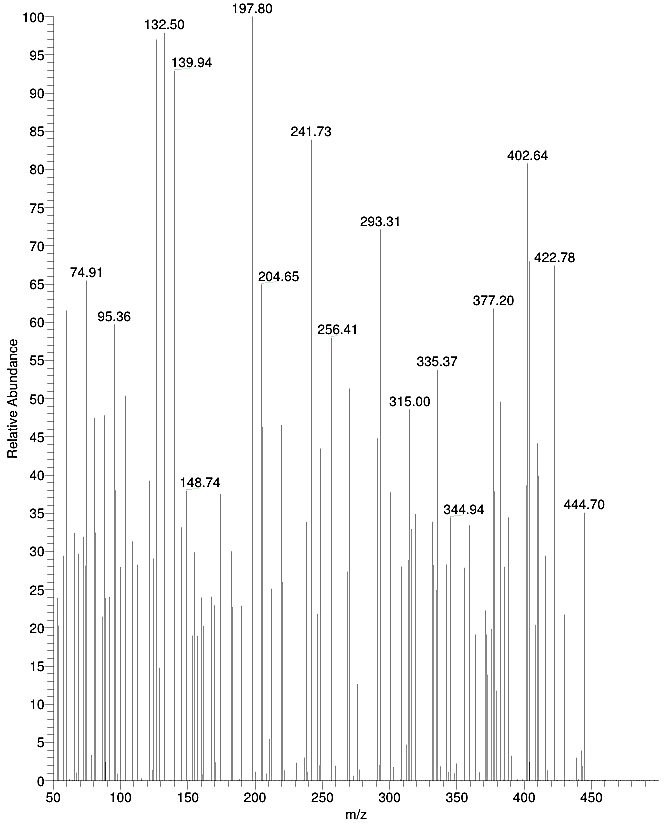


**Fig. S14.** The mass spectrum of [Zn(L).H_2_O]2H_2_O


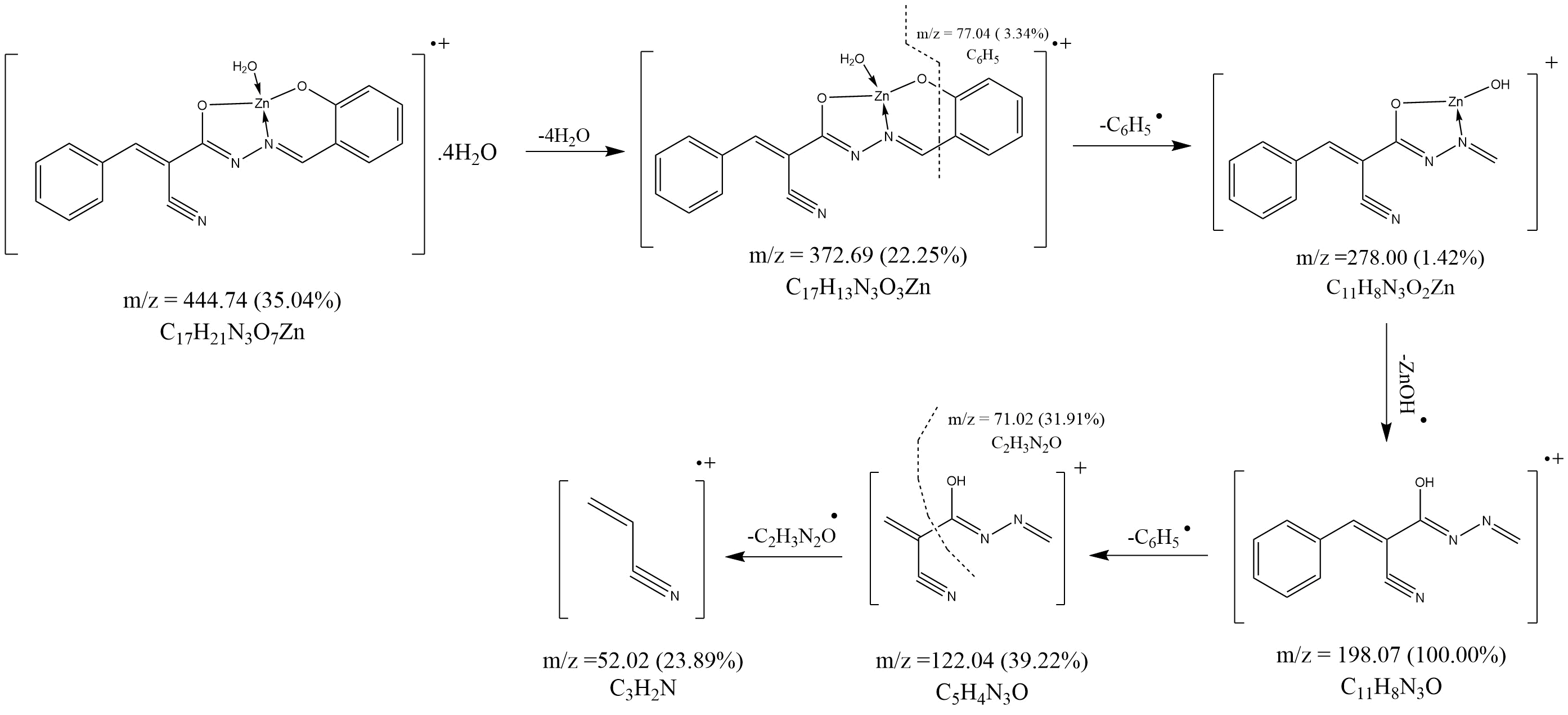


**Scheme S5.** Fragmentation pattern of [Zn(L).H_2_O]2H_2_O


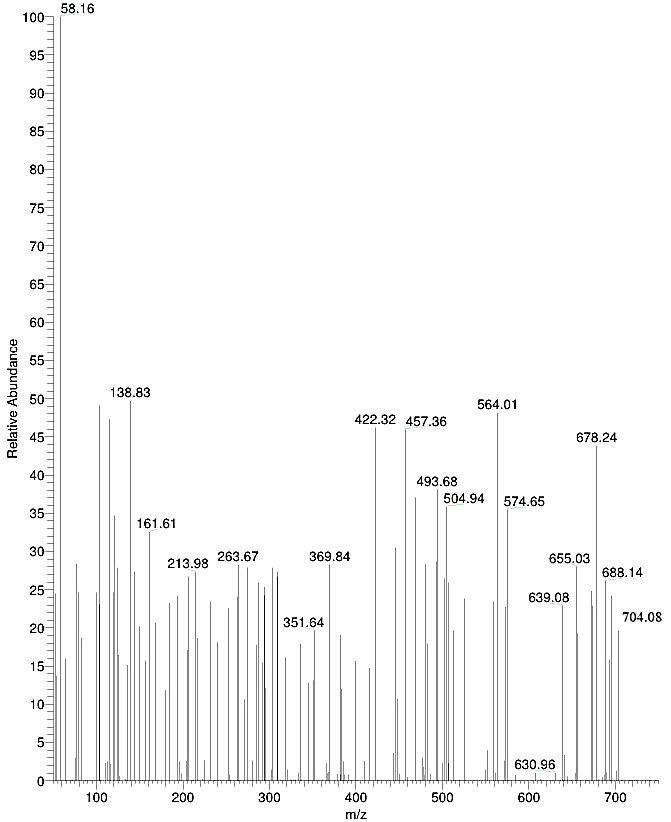


**Fig. S15.** The mass spectrum of [Co(HL)_2_.(H_2_O)_2_]$\frac{\boldsymbol{3}}{\boldsymbol{2}}$ H_2_O


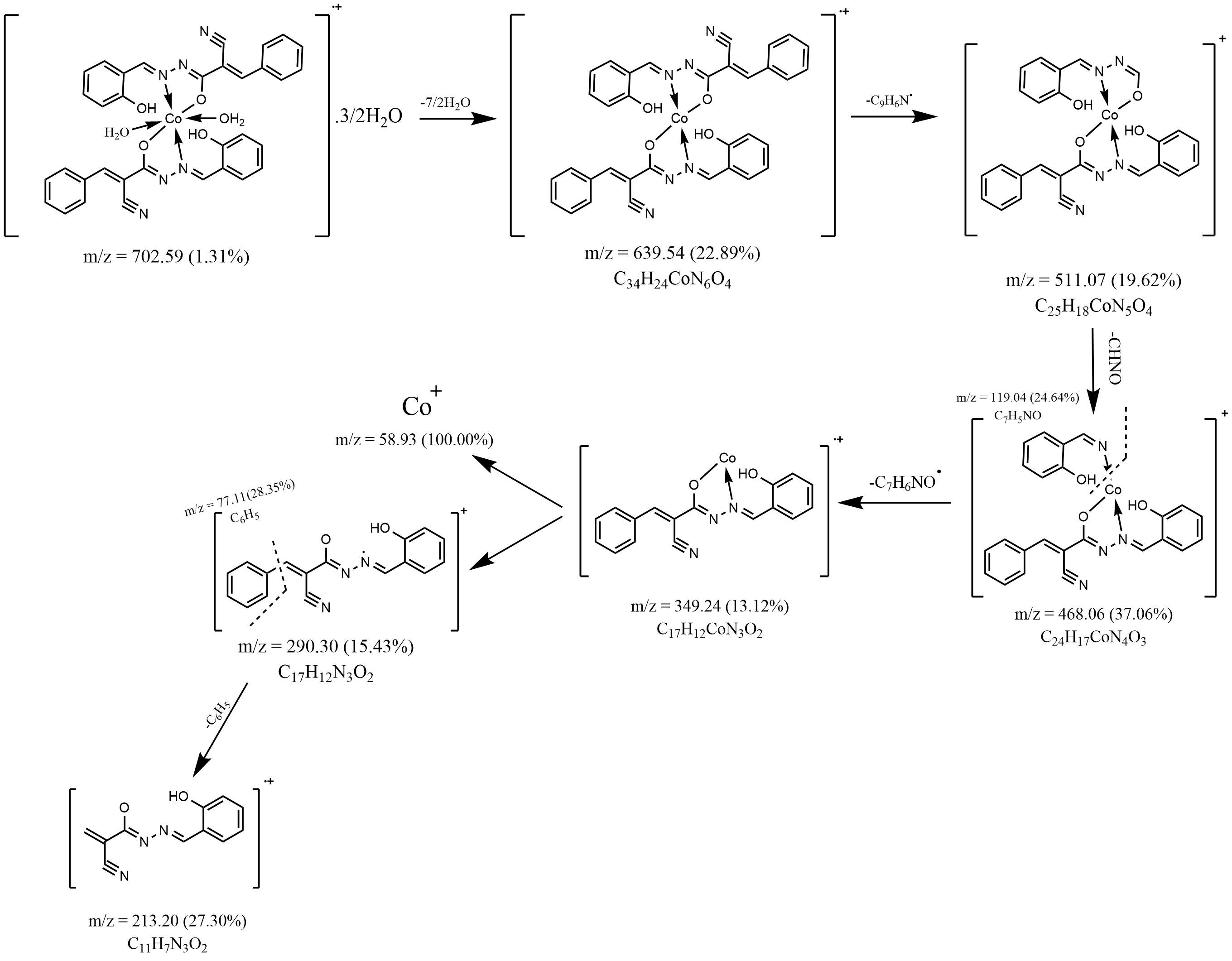


**Scheme S6.** Fragmentation pattern of [Co(HL)_2_.(H_2_O)_2_]$\frac{\boldsymbol{3}}{\boldsymbol{2}}$ H_2_O


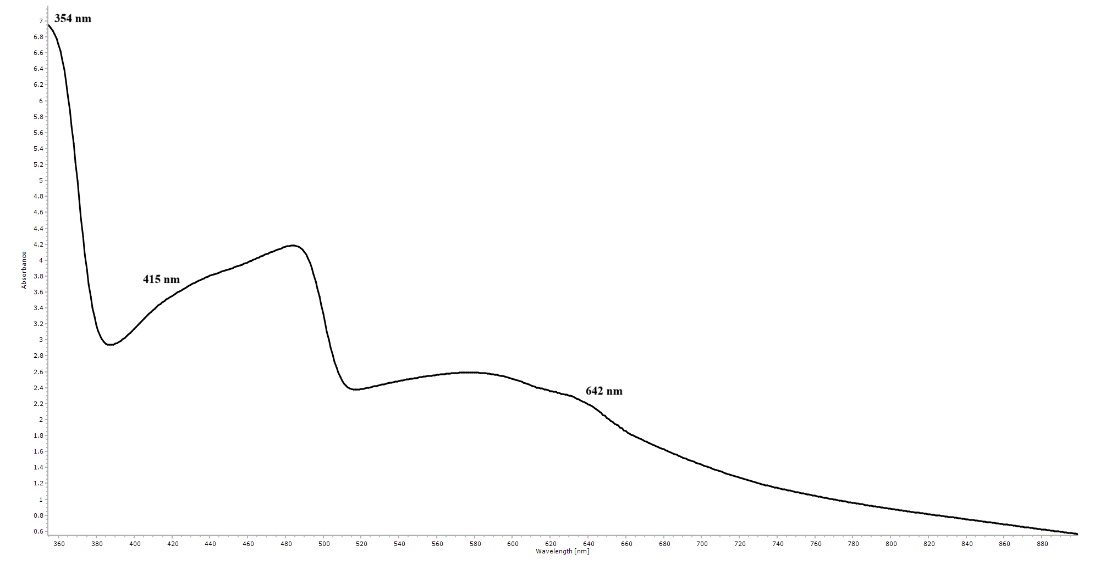


**Fig. S16.** The electronic spectrum of [Ni(L).(H_2_O)_3_]3H_2_O


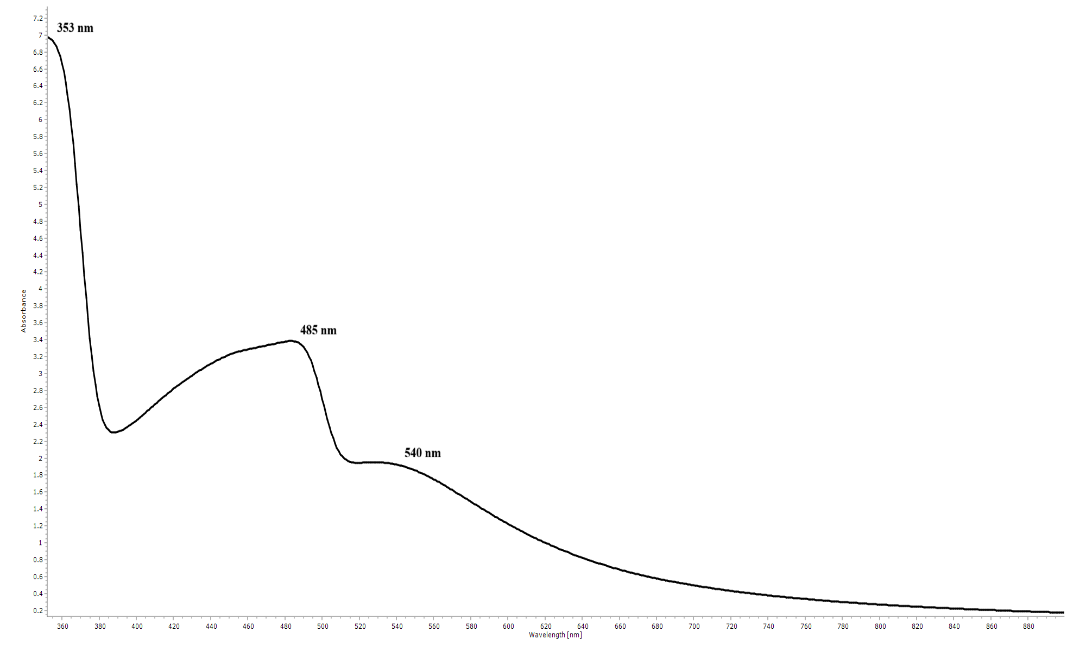


**Fig. S17.** The electronic spectrum of [Co(L)_2_.2H_2_O].$\frac{\boldsymbol{3}}{\boldsymbol{2}}$ H_2_O

**Table S3.** UV-Visible bands and **µ_eff._** of the metal chelates

| Compound | Assignments(cm^-1^) | μ_eff_ (B.M.) |
| --- | --- | --- |
| H_2_L | 22303 (n→π*) | - |
| [Ni(L).(H_2_O)_3_]3H_2_O | 20751 (n→π*),  28272 (LMCT),  24096  ^3^A_2g_ → ^3^T_1g_ (P),  15576 ^3^A_2g_→ ^3^T_1g_ (F) | 3.31 |
| [Cu(L).H_2_O]2H_2_O | 22321 (n→π*),  27048 (LMCT),  18908 (^2^B_1g_→^2^E_g_),  14931 (^2^B_1g_→^2^A_1g_) | 1.88 |
| [Co(HL)_2_.(H_2_O)_2_]$\frac{\boldsymbol{3}}{\boldsymbol{2}}$ H_2_O | 22222 (n→π*),  28321 (LMCT),  20602 ^4^T_1g_(F)→^4^T_1g_(P),  18512 ^4^T_1g_(F)→^4^A_2g_ | 5.20 |
| [Zn(L).H_2_O]4H_2_O | 22123 (n→π*),  28409 (LMCT) | Diamagnetic |


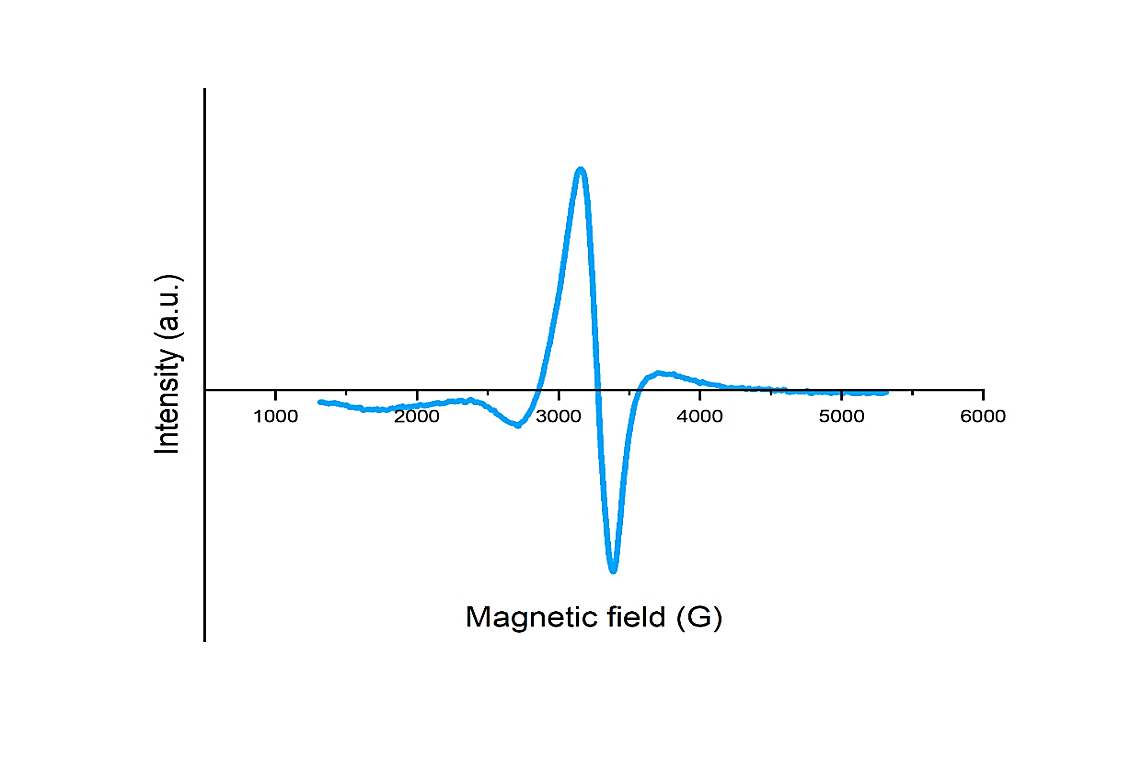


**Fig. S18.** ESR of [Cu(L).H_2_O]2H_2_O


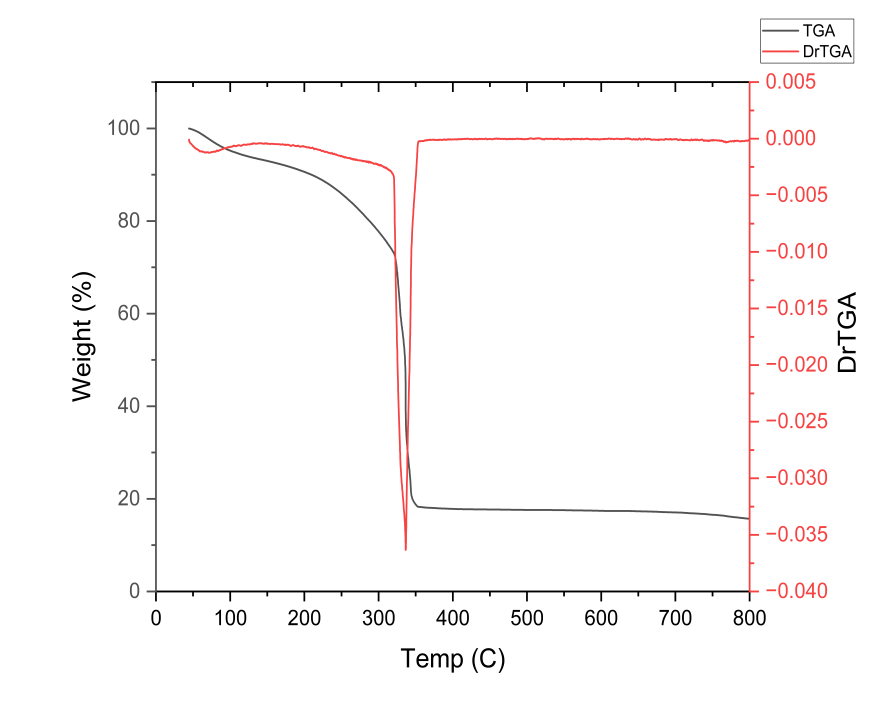


**Fig. S19.** TGA curve of the [Ni(L).(H_2_O)_3_]3H_2_O complex


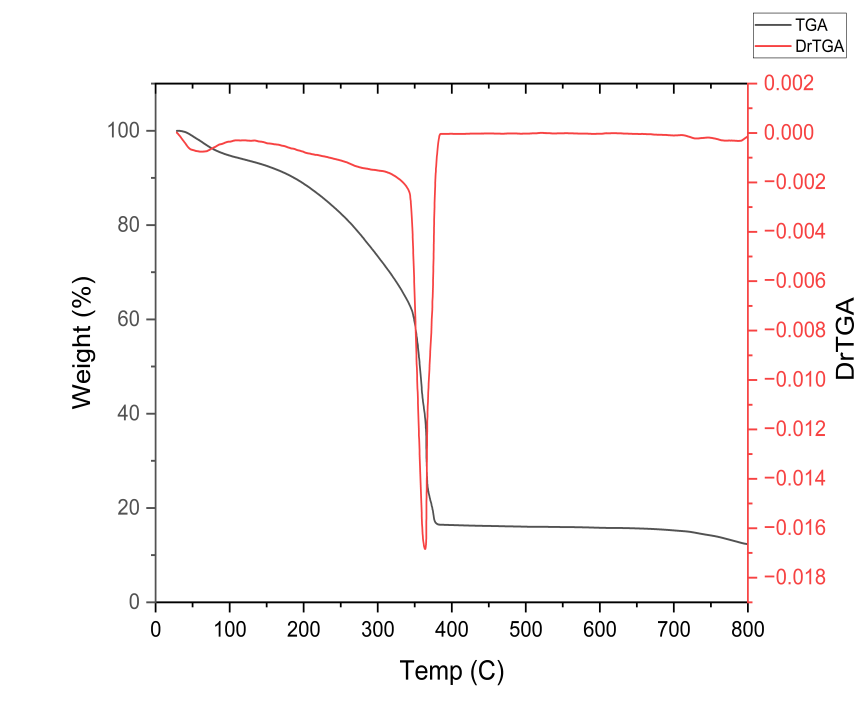


**Fig. S20.** TGA curve of the [Co(HL)_2_.(H_2_O)_2_]$\frac{\boldsymbol{3}}{\boldsymbol{2}}$ H_2_O complex


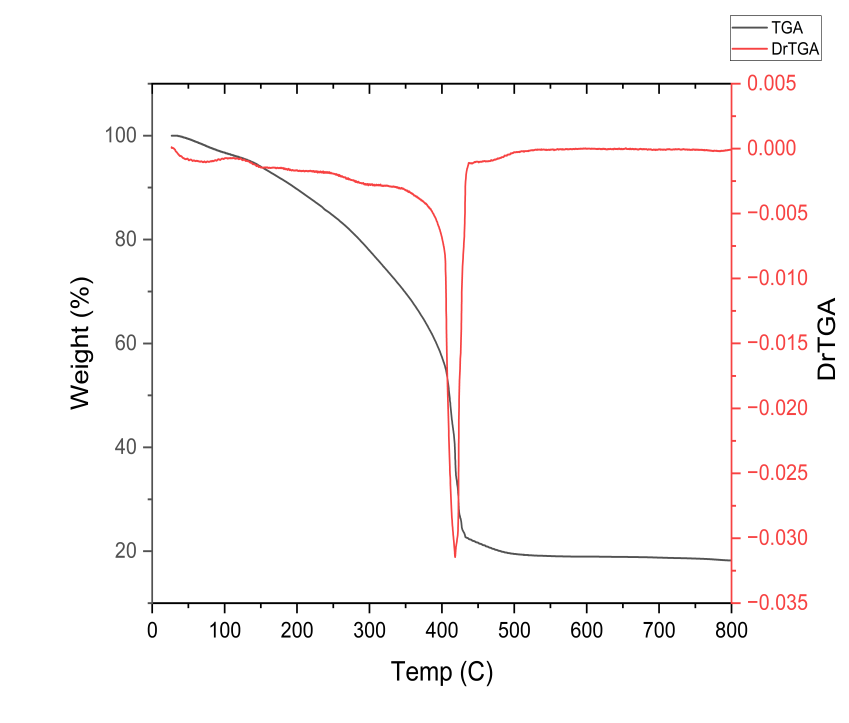


**Fig. S21.** TGA curve of the [Zn(L).H_2_O]4H_2_O complex

**Table S4.** Stages of the decomposition of the metal complexes and their weights lost

| Compound | Temperature range (^o^C) | | Wt. loss (Found %) | | Fragment (Calcd. %) | |  |
| --- | --- | --- | --- | --- | --- | --- | --- |
| [Ni(L).(H_2_O)_3_]3H_2_O | | 45- 120  120- 370  307- 334  334- 338  338- 800  >800 | 5.95  18.15  22.92  21.36  16.08  15.7 | | 1.5H_2_O (5.92)  4.5 H_2_O (17.78)  C_6_H_4_NO(23.24)  C_6_H_4_N(21.93)  C_5_H_3_N(16.89)  NiO(16.38) | |  |
| [Cu(L).H_2_O]2H_2_O | | 30-140  140- 295  295-373  373-800  >800 | 1.93  40.37  21.31  19.99  20.49 | | 0.5H_2_O (2.21)  2.5H_2_O+ C_7_H_4_NO (40.09)  C_5_H_4_N (19.19)  C_5_H_3_N (18.90)  CuO (19.6) | |  |
| [Co(HL)_2_.(H_2_O)_2_]$\frac{\boldsymbol{3}}{\boldsymbol{2}}$ H_2_O | | 36- 90  90- 190  190- 266  266- 342  342-359  359- 367  367- 379  379-800  >800 | 4.84  5.16  10.04  17.01  24.04  17.23  9.64  4.94  11.7 | | 1.5H_2_O (3.85)  2H_2_O (5.13)  C_4_H_3_N (9.26)  C_7_H_5_NO (16.95)  C_8_H_6_N_2_O (24.07)  C_7_H_5_NO (16.95)  C_4_H_3_N (9.26)  C_4_H_2_ (7.12)  CoO (10.68) | |  |
| [Zn(L).H_2_O]4H_2_O | | 30- 120  120-273  273- 355  355-401  401-418  418-800  >800 | | 4.34  16.22  12.72  11.54  16.51  17.56  18.21 | | H_2_O (4.05)  4H_2_O (16.22)  C_3_H_2_N (11.7)  C_3_H_5_ (9.23)  C_5_H_2_N (17.12)  C_5_H_2_N (17.12)  ZnO (18.21) | |

Table **S5. Selected DFT bond lengths of H_2_L^1^ and its metal complexes (Å)**

|  | **Ligand** | **Zn(II) Complex** | **Cu(II) Complex** | **Co(II) Complex** | **Ni(II) Complex** |
| --- | --- | --- | --- | --- | --- |
| O(21)-H | 0.972 | -- | -- | -- | -- |
| O(35)-H(37) | -- | 0.948 | 0.965 | 1.006 | 0.975 |
| O(35)-H(36) | -- | 0.945 | 0.97 | 1.027 | 0.969 |
| M(29)-O(35) | -- | 2.053 | 1.999 | 1.719 | 2.133 |
| O(21)-M(29) | -- | 1.907 | 1.892 | -- | 1.996 |
| C(20)-H(34) | 1.081 | 1.073 | 1.084 | 1.065 | 1.084 |
| C(19)-H(33) | 1.081 | 1.07 | 1.081 | 1.065 | 1.081 |
| C(19)-C(20) | 1.392 | 1.374 | 1.383 | 1.388 | 1.386 |
| C(18)-H(32) | 1.082 | 1.072 | 1.083 | 1.066 | 1.083 |
| C(18)-C(19) | 1.401 | 1.398 | 1.409 | 1.397 | 1.409 |
| C(17)-H(31) | 1.084 | 1.07 | 1.081 | 1.065 | 1.082 |
| C(17)-C(18) | 1.396 | 1.375 | 1.386 | 1.389 | 1.387 |
| C(16)-O(21) | 1.399 | 1.333 | 1.347 | 1.359 | 1.339 |
| C(16)-C(17) | 1.395 | 1.404 | 1.414 | 1.393 | 1.421 |
| C(15)-C(20) | 1.408 | 1.406 | 1.42 | 1.406 | 1.421 |
| C(15)-C(16) | 1.408 | 1.417 | 1.435 | 1.409 | 1.441 |
| C(14)-H(30) | 1.09 | 1.077 | 1.087 | 1.069 | 1.089 |
| C(14)-C(15) | 1.459 | 1.454 | 1.437 | 1.443 | 1.445 |
| N(13)-M(29) | -- | 2.032 | 1.919 | 1.83 | 1.989 |
| N(13)-C(14) | 1.296 | 1.275 | 1.306 | 1.318 | 1.304 |
| N(11)-H | 1.016 | -- | -- | -- | -- |
| O(12)-M(29) | -- | 1.963 | 1.94 | 1.933 | 2.065 |
| N(11)-N(13) | 1.38 | 1.393 | 1.405 | 1.39 | 1.411 |
| C(10)-N(22) | 1.17 | 1.142 | 1.167 | 1.15 | 1.169 |
| C(9)-O(12) | 1.244 | 1.319 | 1.336 | 1.339 | 1.318 |
| C(9)-N(11) | 1.378 | 1.295 | 1.326 | 1.298 | 1.335 |
| C(8)-C(10) | 1.423 | 1.433 | 1.429 | 1.438 | 1.427 |
| C(8)-C(9) | 1.51 | 1.478 | 1.477 | 1.451 | 1.487 |
| C(7)-H(28) | 1.086 | 1.071 | 1.084 | 1.065 | 1.084 |
| C(7)-C(8) | 1.36 | 1.339 | 1.362 | 1.369 | 1.363 |
| C(6)-H(27) | 1.079 | 1.066 | 1.078 | 1.063 | 1.078 |
| C(5)-C(7) | 1.456 | 1.468 | 1.458 | 1.455 | 1.458 |
| C(5)-C(6) | 1.413 | 1.398 | 1.413 | 1.406 | 1.414 |
| C(4)-H(26) | 1.083 | 1.072 | 1.083 | 1.064 | 1.083 |
| C(4)-C(5) | 1.414 | 1.399 | 1.415 | 1.404 | 1.416 |
| C(3)-H(25) | 1.082 | 1.071 | 1.082 | 1.065 | 1.082 |
| C(3)-C(4) | 1.393 | 1.384 | 1.393 | 1.391 | 1.394 |
| C(2)-H(24) | 1.082 | 1.071 | 1.082 | 1.064 | 1.082 |
| C(2)-C(3) | 1.398 | 1.387 | 1.398 | 1.393 | 1.399 |
| C(1)-H(23) | 1.082 | 1.071 | 1.082 | 1.065 | 1.082 |
| C(1)-C(6) | 1.393 | 1.385 | 1.394 | 1.391 | 1.394 |
| C(1)-C(2) | 1.4 | 1.388 | 1.399 | 1.393 | 1.401 |

**Table S6. Selected DFT bond angles of H_2_L^1^ and its metal complexes (°)**

|  | **Ligand** | **Cu(II) complex** | **Zn(II) complex** | **Co(II) complex** | **Ni(II) complex** |
| --- | --- | --- | --- | --- | --- |
| H-O(21)-C(16) | 112.795 | -- | -- | -- | -- |
| H(37)-O(35)-H(36) | -- | 115.413 | 115.876 | 108.77 | 111.23 |
| H(37)-O(35)-M(29) | -- | 132.36 | 103.983 | 99.476 | 100.757 |
| H(36)-O(35)-M(29) | -- | 112.227 | 140.141 | 105.365 | 125.1 |
| O(35)-M(29)-O(21) | -- | 83.973 | 79.256 | -- | 80.363 |
| O(38)-M(29)-O(35) | -- | -- | -- | 179.715 | 79.49 |
| O(38)-M(29)-N(13) | -- | -- | -- | 83.374 | 94.719 |
| O(38)-M(29)-O(12) | -- | -- | -- | 90.842 | 91.23 |
| O(35)-M(29)-N(13) | -- | 179.168 | 179.999 | 96.912 | 170.488 |
| O(35)-M(29)-O(12) | -- | 98.065 | 100.933 | 89.202 | 107.784 |
| O(21)-M(29)-N(13) | -- | 95.195 | 91.342 | -- | 92.647 |
| O(21)-M(29)-O(12) | -- | 177.962 | 170.408 | -- | 170.938 |
| N(13)-M(29)-O(12) | -- | 82.767 | 79.067 | 81.573 | 79.689 |
| M(29)-O(21)-C(16) | -- | 126.331 | 129.322 | -- | 126.261 |
| H(34)-C(20)-C(19) | 121.007 | 119.903 | 119.479 | 119.434 | 119.645 |
| H(34)-C(20)-C(15) | 117.936 | 118.258 | 118.41 | 119.566 | 118.086 |
| C(19)-C(20)-C(15) | 121.057 | 121.839 | 122.111 | 120.998 | 122.269 |
| H(33)-C(19)-C(20) | 119.976 | 120.482 | 120.689 | 120.232 | 120.552 |
| H(33)-C(19)-C(18) | 120.151 | 120.444 | 120.71 | 119.587 | 120.584 |
| C(20)-C(19)-C(18) | 119.873 | 119.074 | 118.601 | 120.18 | 118.864 |
| H(32)-C(18)-C(19) | 120.312 | 119.791 | 119.748 | 119.733 | 119.867 |
| H(32)-C(18)-C(17) | 119.581 | 119.497 | 119.501 | 120.15 | 119.584 |
| C(19)-C(18)-C(17) | 120.107 | 120.711 | 120.75 | 120.117 | 120.549 |
| H(31)-C(17)-C(18) | 120.359 | 121.249 | 121.223 | 119.62 | 120.806 |
| H(31)-C(17)-C(16) | 119.965 | 117.437 | 117.277 | 120.914 | 117.253 |
| C(18)-C(17)-C(16) | 119.676 | 121.314 | 121.499 | 119.466 | 121.941 |
| O(21)-C(16)-C(17) | 121.992 | 117.955 | 118.169 | 117.433 | 118.398 |
| O(21)-C(16)-C(15) | 116.804 | 123.681 | 123.761 | 120.943 | 123.954 |
| C(17)-C(16)-C(15) | 121.204 | 118.364 | 118.07 | 121.609 | 117.645 |
| C(20)-C(15)-C(14) | 122.103 | 117.485 | 116.689 | 117.681 | 116.466 |
| C(16)-C(15)-C(20) | 118.082 | 118.697 | 118.968 | 117.621 | 118.732 |
| C(16)-C(15)-C(14) | 119.815 | 123.818 | 124.343 | 124.538 | 124.801 |
| H(30)-C(14)-N(13) | 122.024 | 116.848 | 118.221 | 112.39 | 117.563 |
| C(15)-C(14)-H(30) | 117.128 | 118.512 | 117.54 | 117.841 | 117.133 |
| C(15)-C(14)-N(13) | 120.849 | 124.64 | 124.238 | 129.738 | 125.304 |
| M(29)-N(13)-C(14) | -- | 126.334 | 126.994 | 135.393 | 126.665 |
| M(29)-N(13)-N(11) | -- | 114.388 | 113.531 | 112.605 | 115.501 |
| C(14)-N(13)-N(11) | 116.897 | 119.278 | 119.475 | 111.91 | 117.825 |
| H-N(11)-N(13) | 119.007 | -- | -- | -- | -- |
| H-N(11)-C(9) | 120.213 | -- | -- | -- | -- |
| M(29)-O(12)-C(9) | -- | 109.397 | 112.251 | 111.642 | 109.234 |
| N(13)-N(11)-C(9) | 120.779 | 109.897 | 111.022 | 114.743 | 110.622 |
| N(22)-C(10)-C(8) | 176.83 | 179.115 | 179.292 | 178.065 | 179.978 |
| O(12)-C(9)-N(11) | 123.825 | 123.552 | 124.129 | 115.432 | 124.934 |
| O(12)-C(9)-C(8) | 121.29 | 118.587 | 117.916 | 119.528 | 118.678 |
| N(11)-C(9)-C(8) | 114.885 | 117.861 | 117.955 | 125.011 | 116.388 |
| C(10)-C(8)-C(9) | 118.295 | 116.66 | 116.366 | 115.372 | 116.535 |
| C(10)-C(8)-C(7) | 124.864 | 124.42 | 124.484 | 123.259 | 124.418 |
| C(9)-C(8)-C(7) | 116.841 | 118.919 | 119.15 | 121.366 | 119.047 |
| H(28)-C(7)-C(5) | 115.149 | 114.66 | 114.045 | 114.189 | 114.526 |
| C(8)-C(7)-H(28) | 112.65 | 113.119 | 113.284 | 118.449 | 112.805 |
| C(8)-C(7)-C(5) | 132.201 | 132.221 | 132.671 | 127.354 | 132.668 |
| H(27)-C(6)-C(5) | 120.57 | 120.512 | 120.853 | 120.454 | 120.595 |
| H(27)-C(6)-C(1) | 118.933 | 118.947 | 118.658 | 118.895 | 118.8 |
| C(5)-C(6)-C(1) | 120.497 | 120.541 | 120.489 | 120.646 | 120.605 |
| C(7)-C(5)-C(6) | 125.061 | 125.327 | 125.451 | 122.953 | 125.385 |
| C(7)-C(5)-C(4) | 116.816 | 116.777 | 116.442 | 118.632 | 116.678 |
| C(6)-C(5)-C(4) | 118.124 | 117.896 | 118.107 | 118.382 | 117.937 |
| H(26)-C(4)-C(5) | 118.991 | 118.902 | 119.203 | 119.625 | 119.008 |
| H(26)-C(4)-C(3) | 119.827 | 119.727 | 119.419 | 119.513 | 119.706 |
| C(5)-C(4)-C(3) | 121.182 | 121.371 | 121.378 | 120.861 | 121.286 |
| H(25)-C(3)-C(2) | 120.215 | 120.204 | 120.302 | 119.956 | 120.211 |
| C(4)-C(3)-H(25) | 119.91 | 119.907 | 119.918 | 119.98 | 119.859 |
| C(4)-C(3)-C(2) | 119.875 | 119.889 | 119.781 | 120.064 | 119.93 |
| H(24)-C(2)-C(3) | 120.136 | 120.207 | 120.213 | 120.097 | 120.213 |
| H(24)-C(2)-C(1) | 120.077 | 120.174 | 120.153 | 120.085 | 120.132 |
| C(3)-C(2)-C(1) | 119.787 | 119.619 | 119.634 | 119.818 | 119.655 |
| H(23)-C(1)-C(2) | 119.991 | 119.976 | 120.057 | 119.888 | 119.938 |
| C(6)-C(1)-H(23) | 119.474 | 119.34 | 119.331 | 119.898 | 119.476 |
| C(6)-C(1)-C(2) | 120.535 | 120.684 | 120.612 | 120.214 | 120.586 |

**Table S7. Selected DFT dihedral angles of H_2_L^1^ and its metal complexes (°).**

|  | Ligand | Cu(II) complex | Zn(II) complex | Co(II) complex | Ni(II) complex |
| --- | --- | --- | --- | --- | --- |
| O(35)-M(29)-O(38)-H(39) | -- | -- | -- | -159.334 | 140.823 |
| O(35)-M(29)-O(38)-H(42) | -- | -- | -- | -46.85 | 8.644 |
| O(40)-M(29)-O(38)-H(39) | -- | -- | -- | -- | 121.25 |
| O(40)-M(29)-O(38)-H(42) | -- | -- | -- | -- | -10.93 |
| O(12)-M(29)-O(35)-H(36) | -- | -179.94 | 0.108 | 127.399 | -43.94 |
| O(12)-M(29)-O(35)-H(37) | -- | 0.026 | 179.984 | -120.008 | -169.676 |
| N(13)-M(29)-O(35)-H(36) | -- | 1.248 | -179.769 | 45.987 | 175.085 |
| N(13)-M(29)-O(35)-H(37) | -- | -178.786 | 0.108 | 158.58 | 49.35 |
| O(21)-M(29)-O(35)-H(36) | -- | 0.033 | -179.891 | -- | 131.969 |
| O(21)-M(29)-O(35)-H(37) | -- | 179.999 | -0.014 | -- | 6.233 |
| C(16)-O(21)-M(29)-O(12) | -- | -0.825 | 0.268 | -- | -38.771 |
| C(16)-O(21)-M(29)-N(13) | -- | -0.059 | -0.143 | -- | -6.732 |
| C(16)-O(21)-M(29)-O(35) | -- | 179.923 | 179.856 | -- | 166.775 |
| C(18)-C(19)-C(20)-C(15) | 0.005 | -0.009 | 0.001 | 0.667 | -0.099 |
| C(18)-C(19)-C(20)-H(34) | 180 | -179.997 | -179.989 | -178.779 | 179.942 |
| H(33)-C(19)-C(20)-C(15) | -180 | -179.999 | 179.998 | -179.582 | 179.9 |
| H(33)-C(19)-C(20)-H(34) | -0.005 | 0.012 | 0.007 | 0.972 | -0.059 |
| C(17)-C(18)-C(19)-C(20) | -0.005 | 0.004 | -0.01 | 0.125 | -0.039 |
| C(17)-C(18)-C(19)-H(33) | 180 | 179.995 | 179.994 | -179.628 | 179.963 |
| H(32)-C(18)-C(19)-C(20) | -179.999 | -179.994 | -179.994 | -179.583 | 179.962 |
| H(32)-C(18)-C(19)-H(33) | 0.006 | -0.004 | 0.01 | 0.664 | -0.037 |
| C(16)-C(17)-C(18)-C(19) | 0 | 0.001 | 0.009 | -0.363 | 0.087 |
| C(16)-C(17)-C(18)-H(32) | 179.994 | 180 | 179.993 | 179.343 | -179.913 |
| H(31)-C(17)-C(18)-C(19) | -179.994 | 179.996 | -179.992 | 179.797 | -179.943 |
| H(31)-C(17)-C(18)-H(32) | 0 | -0.006 | -0.008 | -0.496 | 0.057 |
| C(15)-C(16)-O(21)-H | 179.995 | -- | -- | -- | -- |
| C(17)-C(16)-O(21)-H | 0 | -- | -- | -- | -- |
| C(15)-C(16)-O(21)-M(29) | -- | 0.046 | 0.136 | -- | 5.675 |
| C(17)-C(16)-O(21)-M(29) | -- | -179.955 | -179.87 | -- | -174.972 |
| C(15)-C(16)-C(17)-C(18) | 0.005 | -0.002 | 0 | -0.182 | 0.001 |
| C(15)-C(16)-C(17)-H(31) | 179.999 | -179.997 | -179.999 | 179.655 | -179.971 |
| O(21)-C(16)-C(17)-C(18) | 180 | 180 | -179.995 | 178.419 | -179.394 |
| O(21)-C(16)-C(17)-H(31) | -0.006 | 0.004 | 0.006 | -1.743 | 0.635 |
| C(14)-C(15)-C(20)-C(19) | 179.995 | -179.997 | -179.979 | -176.793 | 179.778 |
| C(14)-C(15)-C(20)-H(34) | 0 | -0.008 | 0.012 | 2.652 | -0.262 |
| C(16)-C(15)-C(20)-C(19) | 0 | 0.008 | 0.007 | -1.174 | 0.185 |
| C(16)-C(15)-C(20)-H(34) | -179.995 | 179.997 | 179.998 | 178.271 | -179.855 |
| C(14)-C(15)-C(16)-C(17) | 180 | -179.997 | 179.977 | 176.226 | -179.689 |
| C(14)-C(15)-C(16)-O(21) | 0.004 | 0.002 | -0.028 | -2.327 | -0.331 |
| C(20)-C(15)-C(16)-C(17) | -0.005 | -0.003 | -0.008 | 0.936 | -0.132 |
| C(20)-C(15)-C(16)-O(21) | 180 | 179.996 | 179.987 | -177.617 | 179.226 |
| N(13)-C(14)-C(15)-C(16) | -180 | -0.018 | -0.021 | 62.146 | -1.946 |
| N(13)-C(14)-C(15)-C(20) | 0.005 | 179.988 | 179.965 | -122.567 | 178.488 |
| H(30)-C(14)-C(15)-C(16) | -0.005 | 179.984 | 179.999 | -120.051 | 178.07 |
| H(30)-C(14)-C(15)-C(20) | 180 | -0.01 | -0.015 | 55.236 | -1.496 |
| N(11)-N(13)-C(14)-C(15) | 180 | 179.995 | -179.974 | -178.967 | 179.618 |
| N(11)-N(13)-M(29)-O(12) | -- | 0.008 | 0.106 | 18.057 | -1.258 |
| N(11)-N(13)-M(29)-O(21) | -- | -179.965 | -179.964 | -- | -176.386 |
| N(11)-N(13)-M(29)-O(35) | -- | 178.822 | 179.877 | 106.215 | 141.194 |
| N(11)-N(13)-M(29)-O(38) | -- | -- | -- | -73.786 | 89.136 |
| N(11)-N(13)-M(29)-O(40) | -- | -- | -- | -- | -88.4 |
| C(14)-N(13)-M(29)-O(12) | -- | -179.983 | -179.837 | -165.846 | 179.83 |
| C(14)-N(13)-M(29)-O(21) | -- | 0.044 | 0.093 | -- | 4.702 |
| C(14)-N(13)-M(29)-O(35) | -- | -1.169 | -88.205 | -77.688 | -37.718 |
| C(14)-N(13)-M(29)-O(38) | -- | -- | -- | 102.311 | -89.776 |
| N(11)-N(13)-C(14)-H(30) | 0.005 | -0.007 | 0.005 | 3.135 | -0.398 |
| M(29)-N(13)-C(14)-H(30) | -- | 179.983 | 179.946 | -172.982 | 178.491 |
| M(29)-N(13)-C(14)-C(15) | -- | -0.015 | -0.034 | 4.917 | -1.493 |
| C(9)-O(12)-M(29)-N(13) | -- | -0.008 | -0.11 | -14.787 | 1.144 |
| C(9)-O(12)-M(29)-O(21) | -- | 0.761 | -0.528 | -- | 33.734 |
| C(9)-O(12)-M(29)-O(35) | -- | -179.991 | 179.891 | -111.894 | -172.785 |
| C(9)-O(12)-M(29)-O(38) | -- | -- | -- | 68.388 | -93.429 |
| C(9)-N(11)-N(13)-C(14) | 180 | 179.986 | 179.867 | 163.761 | -179.875 |
| H(29)-N(11)-N(13)-C(14) | -0.005 | -- | -- | -- | -- |
| C(9)-N(11)-N(13)-M(29) | -- | -0.006 | -0.08 | -19.192 | 1.113 |
| C(8)-C(9)-O(12)-M(29) | -- | -179.988 | -179.907 | -169.993 | 179.116 |
| N(11)-C(9)-O(12)-M(29) | -- | 0.008 | 0.109 | 8.172 | -0.968 |
| C(8)-C(9)-N(11)-N(13) | -179.996 | 179.994 | 179.999 | -175.328 | 179.878 |
| C(8)-C(9)-N(11)-H(29) | 0.009 | -- | -- | -- | -- |
| O(12)-C(9)-N(11)-N(13) | -0.011 | -0.001 | -0.018 | 6.622 | -0.04 |
| O(12)-C(9)-N(11)-H(29) | 179.994 | -- | -- | -- | -- |
| C(7)-C(8)-C(10)-N(22) | 180 | 0.116 | -0.998 | 141.342 | 121.199 |
| C(9)-C(8)-C(10)-N(22) | 0.004 | -179.884 | 178.964 | -39.297 | -58.774 |
| C(7)-C(8)-C(9)-N(11) | 179.996 | -179.995 | 179.946 | 167.992 | -179.974 |
| C(7)-C(8)-C(9)-O(12) | 0.01 | 0.001 | -0.038 | -14.031 | -0.05 |
| C(10)-C(8)-C(9)-N(11) | -0.008 | 0.005 | -0.017 | -11.381 | 0 |
| C(10)-C(8)-C(9)-O(12) | -179.994 | -179.999 | 179.998 | 166.595 | 179.924 |
| C(5)-C(7)-C(8)-C(9) | -179.999 | 179.992 | -179.968 | 175.877 | -179.964 |
| C(5)-C(7)-C(8)-C(10) | 0.005 | -0.008 | -0.008 | -4.8 | 0.064 |
| H(28)-C(7)-C(8)-C(9) | -0.004 | 0.003 | -0.028 | -5.259 | 0.054 |
| H(28)-C(7)-C(8)-C(10) | 180 | -179.997 | 179.932 | 174.064 | -179.918 |
| C(4)-C(5)-C(7)-C(8) | 179.998 | -179.982 | 179.535 | 143.969 | -179.645 |
| C(4)-C(5)-C(7)-H(28) | 0.004 | 0.007 | -0.405 | -34.935 | 0.337 |
| C(6)-C(5)-C(7)-C(8) | -0.001 | 0.028 | -0.505 | -38.163 | 0.426 |
| C(6)-C(5)-C(7)-H(28) | -179.996 | -179.982 | 179.555 | 142.932 | -179.592 |
| C(4)-C(5)-C(6)-C(1) | 0.005 | 0.009 | -0.017 | -1.396 | -0.011 |
| C(4)-C(5)-C(6)-H(27) | 179.994 | -179.997 | 179.96 | 177.751 | -179.932 |
| C(7)-C(5)-C(6)-C(1) | -179.996 | 179.999 | -179.976 | -179.269 | 179.917 |
| C(7)-C(5)-C(6)-H(27) | -0.007 | -0.008 | 0 | -0.121 | -0.004 |
| C(3)-C(4)-C(5)-C(6) | 0 | -0.005 | 0.037 | 1.304 | -0.012 |
| C(3)-C(4)-C(5)-C(7) | -180 | -179.995 | 180 | 179.27 | -179.947 |
| H(26)-C(4)-C(5)-C(6) | 179.995 | 179.997 | -179.977 | -178.919 | 179.98 |
| H(26)-C(4)-C(5)-C(7) | -0.004 | 0.006 | -0.013 | -0.953 | 0.046 |
| C(2)-C(3)-C(4)-C(5) | 0 | 0 | -0.027 | -0.438 | 0.018 |
| C(2)-C(3)-C(4)-H(26) | -179.995 | 179.999 | 179.986 | 179.785 | -179.974 |
| H(25)-C(3)-C(4)-C(5) | 180 | -179.997 | 179.981 | 179.582 | -179.99 |
| H(25)-C(3)-C(4)-H(26) | 0.005 | 0.001 | -0.006 | -0.196 | 0.018 |
| C(1)-C(2)-C(3)-C(4) | -0.005 | 0 | -0.003 | -0.366 | -0.001 |
| C(1)-C(2)-C(3)-H(25) | 179.995 | 179.997 | 179.989 | 179.615 | -179.993 |
| H(24)-C(2)-C(3)-C(4) | -179.995 | -179.992 | -179.988 | 179.831 | 179.985 |
| H(24)-C(2)-C(3)-H(25) | 0.005 | 0.005 | 0.004 | -0.188 | -0.007 |
| C(2)-C(1)-C(6)-C(5) | -0.009 | -0.01 | -0.013 | 0.625 | 0.029 |
| C(2)-C(1)-C(6)-H(27) | -179.999 | 179.997 | -179.99 | -178.535 | 179.951 |
| H(23)-C(1)-C(6)-C(5) | -179.999 | 179.996 | 179.987 | -179.571 | -179.959 |
| H(23)-C(1)-C(6)-H(27) | 0.012 | 0.003 | 0.01 | 1.269 | -0.037 |
| C(6)-C(1)-C(2)-C(3) | 0.009 | 0.005 | 0.023 | 0.272 | -0.023 |
| C(6)-C(1)-C(2)-H(24) | -180 | 179.997 | -179.992 | -179.925 | 179.992 |
| H(23)-C(1)-C(2)-C(3) | 179.999 | 179.999 | -179.976 | -179.532 | 179.965 |
| H(23)-C(1)-C(2)-H(24) | -0.011 | -0.009 | 0.008 | 0.271 | -0.021 |

**Table S8.** E_HOMO_, E_LUMO_ and chemical reactivity descriptors of H_2_L and its coordination compounds

| Compound | E_HOMO_ | E_LUMO_ | ΔE_H-L_ | χ | η | δ | ω | E_g_ |
| --- | --- | --- | --- | --- | --- | --- | --- | --- |
| Ligand | -6.326 | -2.835 | 3.491 | 4.5805 | 1.7455 | 0.5729 | 6.01 | 3.3 |
| Cu^2+^ complex | -5.941 | -2.571 | 3.37 | 4.256 | 1.685 | 0.5935 | 5.3749 | 3.26 |
| Co^2+^ complex | -6.265 | -2.804 | 3.461 | 4.5345 | 1.7305 | 0.5779 | 5.941 | 3.27 |
| Ni^2+^ complex | -5.585 | -2.577 | 3.008 | 4.5805 | 1.7455 | 0.5729 | 6.01 | 3.26 |
| Zn^2+^ complex | -5.876 | -2.524 | 3.352 | 4.2 | 1.676 | 0.5967 | 5.2625 | 3.28 |

| Cu(II) complex | 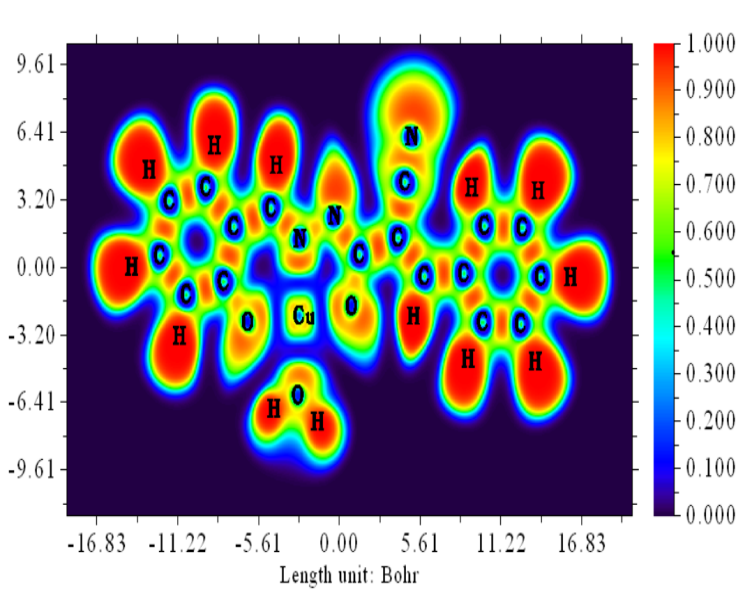 | 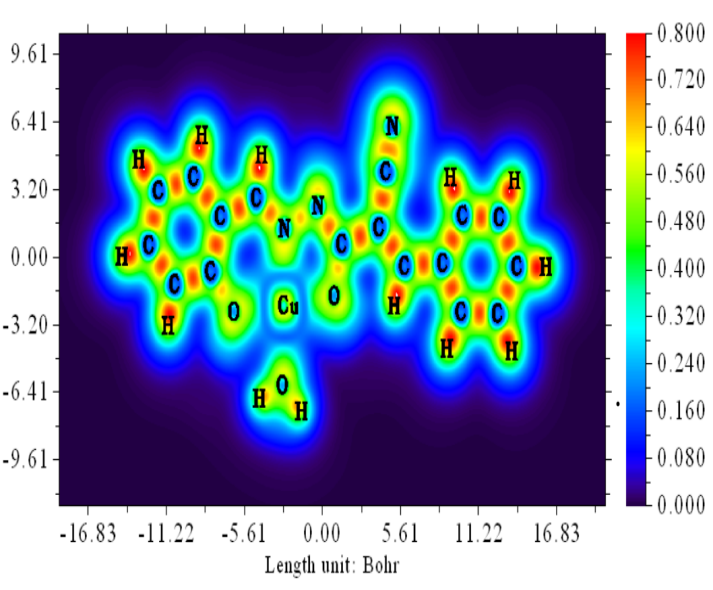 |
| --- | --- | --- |
| Co(II) complex | 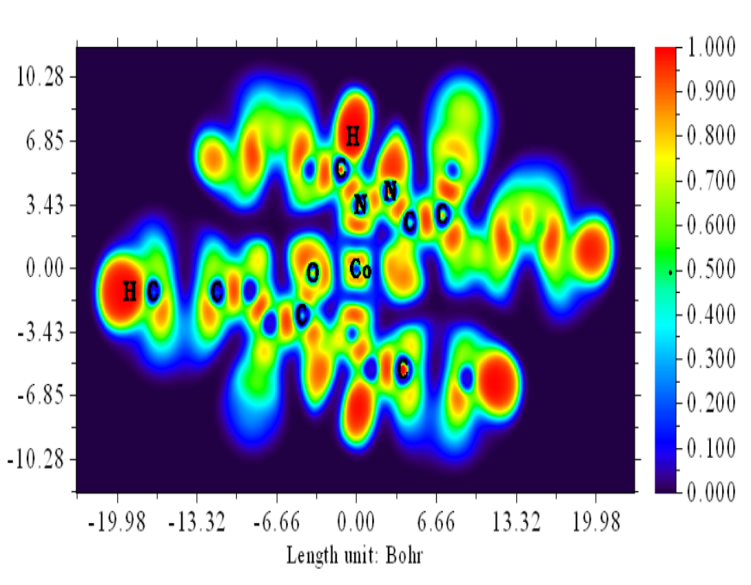 | 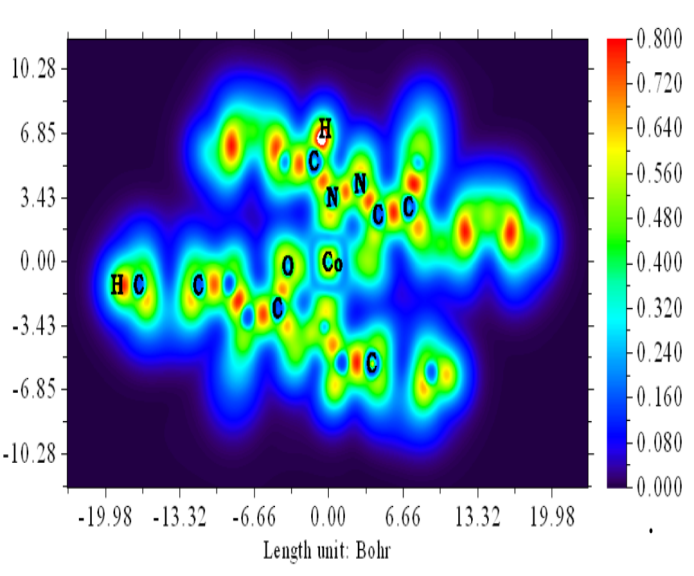 |
| Ni(II) complex | 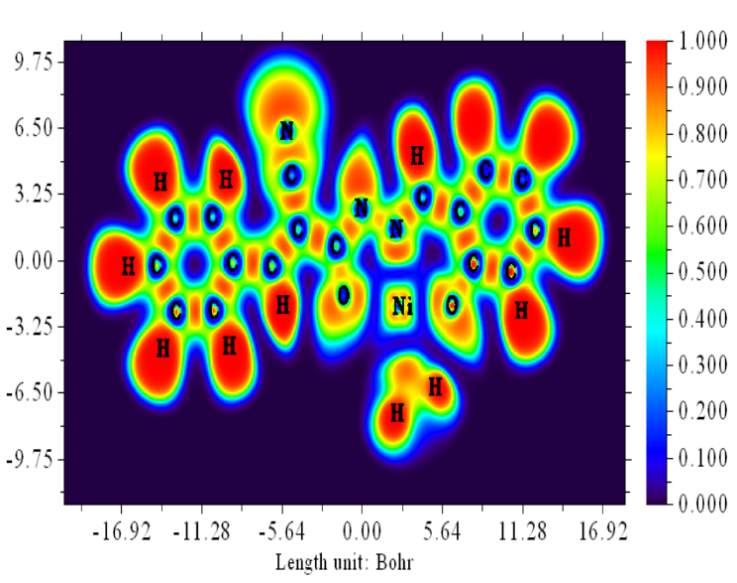 | 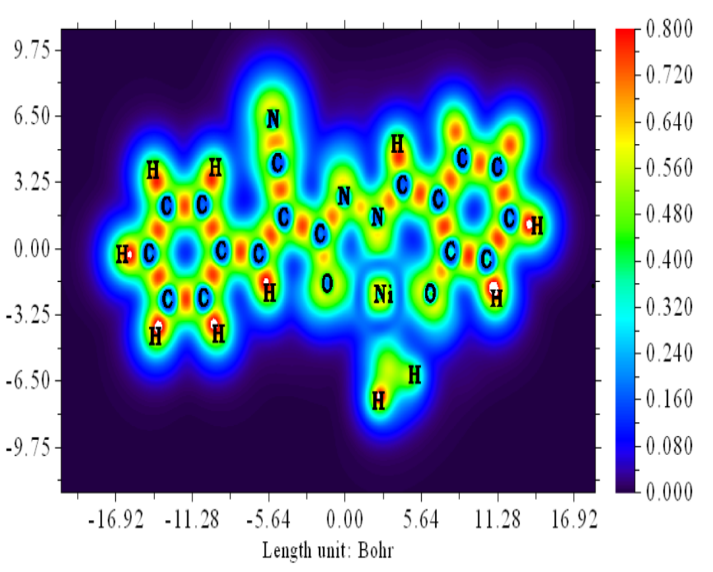 |

**Fig. S22.**

**Table S9.** Selected atom net charges

| Atom | Ligand | Ni^2+^ complex | Cu^2+^ complex | Co^2+^ complex | Zn^2+^ complex |
| --- | --- | --- | --- | --- | --- |
| N11 | -0.4337 | -0.1941 | -0.1752 | -0.2229 | -0.1899 |
| O12 | -0.5812 | -0.3459 | -0.3333 | -0.2469 | -0.4105 |
| N13 | -0.2182 | -0.1204 | -0.1057 | -0.1262 | -0.2246 |
| O21 | -0.6882 | -0.3555 | -0.3218 | -0.3548 | -0.4316 |
| N22 | -0.3110 | -0.1531 | -0.1497 | -0.1634 | -0.1590 |
| M29 | - | 1.3179 | 0.8128 | 1.0553 | 0.6726 |
| O35 | - | -0.4344 | -0.4379 | -0.3300 | -0.4749 |
| O38 | - | -0.4170 | - | -0.3298 | - |
| O50 | - | -0.4229 | - | -0.2476 | - |
| N51 | - | - | - | -0.1634 | - |
| N59 | - | - | - | -0.2220 | - |
| N62 | - | - | - | -0.1266 | - |
| O75 | - | - | - | -0.3547 | - |

**Table S10. The significant stabilization energies E^(2)^ (in kcal/mol) corresponding to donor-acceptor interactions in the ligand and its metal complexes**

|  | Donor | | Acceptor | | E^(2)^ kcal/mol | Donor | | Acceptor | | E^(2)^ kcal/mol |
| --- | --- | --- | --- | --- | --- | --- | --- | --- | --- | --- |
| Ligand | LP (1) | N 11 | π* | C 9 - O 12 | 61.98 | LP (1) | O 12 | σ* | C 7 - H 28 | 6.49 |
|  | LP (1) | N 11 | π* | N 13 - C 14 | 148.31 | LP (2) | O 12 | σ* | C 9 - N 11 | 24.9 |
|  | LP (1) | N 13 | σ* | C 14 - H 30 | 9.12 | LP (1) | O 21 | σ* | N 13 - C 14 | 40.53 |
|  | LP (1) | N 13 | σ* | N 11 - H 29 | 7.33 | LP (1) | O 21 | π* | C 10 - N 22 | 39.95 |
|  | LP (1) | N 13 | σ* | N 13 - C 14 | 9.79 | LP (1) | O 21 | π* | N 13 - C 14 | 83.4 |
|  | LP (1) | N 13 | π* | N 13 - C 14 | 16.13 | LP (2) | O 21 | π* | N 13 - C 14 | 77.06 |
|  | LP (1) | O 12 | σ* | C 17 - H 31 | 10.16 | LP (2) | O 21 | σ* | C 7 - H 28 | 5.51 |
|  | LP (1) | O 12 | σ* | C 20 - H 34 | 386.94 | LP (2) | O 21 | σ* | C 17 - H 31 | 9.49 |
|  | LP (1) | O 12 | σ* | N 13 - C 14 | 281.09 | LP (2) | O 21 | σ* | C 20 - H 34 | 123.32 |
|  | LP (1) | O 12 | π* | N 13 - C 14 | 112.34 | LP (2) | O 21 | σ* | N 13 - C 14 | 178.3 |
|  | LP (1) | O 12 | σ* | C 4 - H 26 | 5.74 |  |  |  |  |  |
| Ni(II) complex | LP (1) | N 11 | σ* | C 9 - O 12 | 6.9 | LP (1) | O 21 | LP*(7) | Ni 29 | 12.13 |
|  | LP (1) | N 11 | σ* | O 40 - H 43 | 25.15 | LP (2) | O 21 | LP*(5) | Ni 29 | 11.92 |
|  | LP (1) | N 13 | σ* | C 9 - N 11 | 6.25 | LP (2) | O 21 | LP*(7) | Ni 29 | 8.93 |
|  | LP (1) | N 13 | LP*(5) | Ni 29 | 52.55 | LP (2) | O 21 | LP*(6) | Ni 29 | 6.66 |
|  | LP (1) | N 13 | LP*(4) | Ni 29 | 49.29 | LP (2) | O 21 | σ* | O 40 - H 43 | 32.2 |
|  | LP (1) | N 13 | LP*(6) | Ni 29 | 18.38 | LP (3) | O 21 | σ* | C 9 - N 11 | 7.49 |
|  | LP (1) | N 13 | LP*(9) | Ni 29 | 15.18 | LP (3) | O 21 | LP*(5) | Ni 29 | 28.38 |
|  | LP (1) | N 13 | σ* | O 40 - H 43 | 75.27 | LP (3) | O 21 | LP*(7) | Ni 29 | 20.36 |
|  | LP (1) | O 12 | σ* | C 7 - H 28 | 411.77 | LP (3) | O 21 | LP*(6) | Ni 29 | 16.06 |
|  | LP (1) | O 12 | LP*(7) | Ni 29 | 6.88 | LP (3) | O 21 | σ* | O 40 - H 43 | 74.05 |
|  | LP (1) | O 12 | σ* | O 40 - H 43 | 67.63 | LP (1) | O 35 | σ* | C 4 - H 26 | 5.32 |
|  | LP (1) | O 12 | σ* | C 9 - N 11 | 16.17 | LP (2) | O 35 | LP*(9) | Ni 29 | 28.33 |
|  | LP (2) | O 12 | σ* | O 40 - H 43 | 80.24 | LP (2) | O 35 | LP*(6) | Ni 29 | 11.75 |
|  | LP (2) | O 12 | σ* | C 7 - H 28 | 160.28 | LP (2) | O 35 | σ* | O 40 - H 43 | 31.45 |
|  | LP (2) | O 12 | σ* | C 9 - N 11 | 6.51 | LP (1) | O 38 | σ* | C 4 - H 26 | 11.57 |
|  | LP (2) | O 12 | σ* | C 9 - O 12 | 7.92 | LP (2) | O 38 | σ* | C 6 - H 27 | 6.14 |
|  | LP (2) | O 12 | σ* | C 20 - H 34 | 7.05 | LP (2) | O 38 | σ* | C 4 - H 26 | 44.29 |
|  | LP (2) | O 12 | LP*(6) | Ni 29 | 34.24 | LP (2) | O 38 | LP*(8) | Ni 29 | 38.7 |
|  | LP (2) | O 12 | LP*(5) | Ni 29 | 25.16 | LP (2) | O 38 | LP*(6) | Ni 29 | 26.27 |
|  | LP (2) | O 12 | LP*(8) | Ni 29 | 20.89 | LP (2) | O 40 | σ* | C 9 - N 11 | 8.97 |
|  | LP (2) | O 12 | LP*(7) | Ni 29 | 13.59 | LP (2) | O 40 | σ* | C 9 - O 12 | 8.22 |
|  | LP (3) | O 12 | π* | C 9 - N 11 | 34.49 | LP (2) | O 40 | σ* | C 20 - H 34 | 7.11 |
|  | LP (3) | O 12 | σ* | O 40 - H 43 | 7.71 | LP (2) | O 40 | LP*(6) | Ni 29 | 72.68 |
|  | LP (1) | O 21 | σ* | O 40 - H 43 | 11.05 | LP (2) | O 40 | LP*(8) | Ni 29 | 55.33 |
| Cu(II) complex | LP (1) | N 11 | σ* | C 9 - O 12 | 5.8 | LP (3) | O 12 | LP*(5) | Cu 29 | 30.3 |
|  | LP (1) | O 12 | σ* | C 9 - N 11 | 11.24 | LP (3) | O 12 | LP*(6) | Cu 29 | 15.42 |
|  | LP (1) | O 12 | σ* | C 17 - H 31 | 6.94 | LP (1) | O 21 | LP*(6) | Cu 29 | 11.24 |
|  | LP (1) | O 12 | σ* | C 16 - O 21 | 29.7 | LP (1) | N 13 | LP*(7) | Cu 29 | 37.30 |
|  | LP (1) | O 12 | LP*(6) | Cu 29 | 10.46 | LP (2) | O 21 | σ* | C 19 - H 33 | 8.04 |
|  | LP (2) | O 12 | π* | C 9 - N 11 | 6.51 | LP (3) | O 21 | σ* | C 17 - H 31 | 6.69 |
|  | LP (2) | O 12 | σ* | C 9 - N 11 | 5.06 | LP (3) | O 21 | LP*(5) | Cu 29 | 32.91 |
|  | LP (2) | O 12 | σ* | C 16 - N 17 | 14.91 | LP (3) | O 21 | LP*(6) | Cu 29 | 18.09 |
|  | LP (2) | O 12 | σ* | C 16 - O 21 | 187.21 | LP (1) | O 35 | σ* | C 19 - H 33 | 166.74 |
|  | LP (2) | O 12 | LP*(7) | Cu 29 | 16.38 | LP (2) | O 35 | σ* | C 16 - N 17 | 13.19 |
|  | LP (2) | O 12 | σ* | N 20 - C 21 | 6.8 | LP (2) | O 35 | σ* | C 17 - H 31 | 19.49 |
|  | LP (3) | O 12 | σ* | C 17 - H 31 | 17.51 | LP (2) | O 35 | σ* | C 16 - O 21 | 75.41 |
|  | LP (3) | O 12 | σ* | C 16 - O 21 | 8.16 | LP (2) | O 35 | LP*(7) | Cu 29 | 67.3 |
| Co(II) complex | LP (1) | N 11 | σ* | C 9 - O 12 | 5.18 | LP (2) | O 38 | σ* | C 69 - H 70 | 20.49 |
|  | LP (2) | O 12 | π* | C 9 - N 11 | 29.88 | LP (2) | O 38 | σ* | C 71 - H 72 | 10.63 |
|  | LP (2) | O 12 | LP*(3) | Co 29 | 7.45 | LP (2) | O 38 | LP*(3) | Co 29 | 22.38 |
|  | LP (1) | N 59 | σ* | C 57 - O 60 | 5.16 | LP (2) | O 38 | π* | N 62 - C 63 | 5.87 |
|  | LP (2) | O 60 | σ* | C 14 - H 30 | 10.59 | LP (2) | O 38 | σ* | O 38 - H 39 | 518.14 |
|  | LP (2) | O 60 | π* | C 57 - N 59 | 28.22 | LP (2) | O 38 | σ* | O 75 - H 41 | 30.87 |
|  | LP (2) | O 60 | LP*(3) | Co 29 | 7.86 | LP (1) | O 21 | σ* | C 6 - H 27 | 15.23 |
|  | LP (2) | O 60 | σ* | O 38 - H 39 | 25.51 | LP (1) | O 21 | σ* | C 14 - H 30 | 190.49 |
|  | LP (1) | N 61 | σ* | C 14 - H 30 | 7.83 | LP (1) | O 21 | σ* | C 17 - H 31 | 6.87 |
|  | LP (1) | N 61 | σ* | O 38 - H 39 | 22.98 | LP (1) | O 21 | σ* | C 57 - N 59 | 11.34 |
|  | LP (1) | O 35 | LP*(4) | Co 29 | 11.99 | LP (1) | O 21 | σ* | C 66 - O 75 | 13.25 |
|  | LP (1) | O 35 | LP*(3) | Co 29 | 7.04 | LP (1) | O 21 | σ* | C 69 - H 70 | 17.9 |
|  | LP (2) | O 35 | LP*(4) | Co 29 | 73.22 | LP (1) | O 21 | σ* | C 71 - H 72 | 12.45 |
|  | LP (2) | O 35 | LP*(3) | Co 29 | 26 | LP (1) | O 21 | π* | N 62 - C 63 | 6.6 |
|  | LP (1) | O 38 | σ* | C 14 - H 30 | 7.28 | LP (1) | O 21 | σ* | O 38 - H 39 | 974.03 |
|  | LP (1) | O 38 | LP*(3) | Co 29 | 8.47 | LP (1) | O 21 | σ* | O 75 - H 41 | 34.04 |
|  | LP (1) | O 38 | σ* | O 38 - H 39 | 16.29 | LP (2) | O 21 | σ* | C 6 - H 27 | 5.27 |
|  | LP (2) | O 38 | σ* | C 6 - H 27 | 10.93 | LP (2) | O 21 | σ* | C 14 - H 30 | 98.33 |
|  | LP (2) | O 38 | σ* | C 14 - H 30 | 160.82 | LP (2) | O 21 | σ* | C 66 - O 75 | 5.13 |
|  | LP (2) | O 38 | σ* | C 17 - H 31 | 7.19 | LP (2) | O 21 | σ* | C 69 - H 70 | 5.91 |
|  | LP (2) | O 38 | σ* | C 57 - N 59 | 10.7 | LP (2) | O 21 | σ* | O 38 - H 39 | 295.34 |
|  | LP (2) | O 38 | σ* | C 66 - O 75 | 13.7 | LP (2) | O 21 | σ* | O 75 - H 41 | 41.04 |
| Zn(II) complex | LP (1) | N 11 | σ* | C 9 - O 12 | 7.05 | LP (1) | O 21 | σ* | C 10 - N 22 | 8.65 |
|  | LP (1) | O 12 | LP*(7) | Zn 29 | 6 | LP (1) | O 21 | LP*(6) | Zn 29 | 8.34 |
|  | LP (2) | O 12 | LP*(6) | Zn 29 | 25.38 | LP (1) | O 21 | LP*(8) | Zn 29 | 8.08 |
|  | LP (2) | O 12 | LP*(7) | Zn 29 | 16.36 | LP (1) | O 21 | LP*(7) | Zn 29 | 7.22 |
|  | LP (3) | O 12 | σ* | C 7 - H 28 | 20.16 | LP (2) | O 21 | σ* | C 7 - H 28 | 9.72 |
|  | LP (3) | O 12 | π* | C 9 - N 11 | 15.01 | LP (2) | O 21 | LP*(7) | Zn 29 | 24.74 |
|  | LP (3) | O 12 | σ* | N 13 - C 14 | 19.93 | LP (2) | O 21 | LP*(6) | Zn 29 | 24.13 |
|  | LP (3) | O 12 | LP*(6) | Zn 29 | 15.96 | LP (3) | O 21 | σ* | C 19 - H 30 | 9.22 |
|  | LP (1) | N 22 | σ* | C 7 - H 28 | 39.54 | LP (1) | O 35 | σ* | C 19 - H 30 | 166.74 |
|  | LP (1) | N 22 | σ* | N 13 - C 14 | 30.1 | LP (2) | O 35 | σ* | C 7 - H 28 | 14.12 |
|  | LP (1) | N 22 | LP*(6) | Zn 29 | 20.29 | LP (2) | O 35 | σ* | C 9 - O 12 | 22.27 |
|  | LP (1) | N 13 | LP*(6) | Zn 29 | 44.19 | LP (2) | O 35 | σ* | C 10 - N 22 | 101.13 |
|  | LP (1) | N 13 | LP*(8) | Zn 29 | 11.4 | LP (2) | O 35 | σ* | N 13 - C 14 | 14.03 |
|  | LP (1) | O 21 | σ* | C 7 - H 28 | 15.03 | LP (2) | O 35 | LP*(8) | Zn 29 | 47.4 |
|  | LP (1) | O 21 | σ* | C 9 - O 12 | 5.57 | LP (2) | O 35 | LP*(6) | Zn 29 | 5.97 |

**Table S11.** μ, α_total_, Δα & β_total_ of the suggested compounds

|  | | H_2_L | Ni^2+^ complex | Cu^2+^ complex | Co^2+^ complex | Zn^2+^ complex |
| --- | --- | --- | --- | --- | --- | --- |
| Total static dipole moment μ (a.u) | $\mu x$ | -0.16066 | 0.28183 | -0.48302 | -0.01199 | 0.65740 |
|  | $\mu y$ | 0.48794 | -3.43338 | -2.90384 | -0.05535 | 3.12268 |
|  | $\mu z$ | 0.00009 | 0.42414 | 0.00105 | 0.12740 | 0.00135 |
|  | $\mu$ | 0.51389 | 3.56592 | 2.94410 | 0.50795 | 3.19155 |
| Polarizability α (a.u) | $\alpha\mathrm{xx}$ | 478.394 | 590.116 | 575.131 | 1703.17 | 570.777 |
|  | $\alpha\mathrm{xy}$ | -17.8983 | 51.1662 | -42.1885 | 271.887 | -46.0461 |
|  | αyy | 234.749 | 284.889 | 266.447 | 795.374 | 265.054 |
|  | $\alpha\mathrm{xz}$ | 0.00058 | 0.51135 | 0.00259 | -47.0593 | -0.00171 |
|  | $\alpha\mathrm{zy}$ | 0.00028 | -3.78602 | 0.01468 | 24.1671 | -0.00768 |
|  | αzz | 108.176 | 147.623 | 120.900 | 330.424 | 121.385 |
|  | Δα | 327.389 | 402.244 | 408.334 | 1300.96 | 405.4517 |
|  | α | 224855 | 348589 | 308788 | 2667681 | 305421 |
| First hyperpolarizability β (a.u) | βxxx | 3791.97 | -7333.21 | 974.889 | 1327.01 | -1149.51 |
|  | βyxx | -601.066 | 157.606 | 163.059 | 906.244 | -169.326 |
|  | βxyy | 67.732 | 93.0742 | -65.313 | 444.335 | -7.57849 |
|  | βyyy | -12.1795 | -92.2863 | -96.520 | -181.490 | -14.1678 |
|  | βzxx | -0.07149 | -16.31617 | 0.29785 | -390.984 | -0.00571 |
|  | βxyz | -0.00300 | 4.73054 | -0.25617 | -192.430 | -0.01582 |
|  | βzyy | 0.03236 | 1.38136 | -0.20472 | 69.8018 | -0.01683 |
|  | βxzz | -4.04472 | -29.5649 | -10.3593 | 12.1520 | 4.28019 |
|  | βyzz | -48.0631 | -120.808 | -18.8977 | -20.6693 | 30.8017 |
|  | βzzz | 0.01137 | 104.778 | 0.04792 | 19.0726 | 0.04719 |
|  | β | 3509.46 | 7297.94 | 922.730 | 2127.88 | 1226.78 |

**Table S12.** The binding modes of H_2_L and its coordination compounds towards 4fm9 and 3ig7 proteins

| **Compound** | **Protein code** | **Ligand** | **Receptor** | **Interaction** | **Distance** | **E(kcal/mol)** |
| --- | --- | --- | --- | --- | --- | --- |
| **H_2_L keto** | **4fm9** | O21  N22  6-ring | GLU 682  LEU 592  LEU 705 | H-donor  H-acceptor  pi-H | 3.15  3.16  4.16 | -0.9  -3.8  -0.5 |
|  | **3ig7** | O21  N22 | GLU 8  LEU 83 | H-donor  H-acceptor | 2.97  3.19 | -1.6  -2.6 |
| **H_2_L enol** | **4fm9** | O21  N22  6-ring | GLU 682  LEU 592  LEU 705 | H-donor  H-acceptor  pi-H | 3.25  3.15  4.22 | -0.7  -3.9  -0.5 |
|  | **3ig7** | O21  N22 | GLU 81  LYS 89 | H-donor  H-acceptor | 2.97  3.12 | -2.2  -1.3 |
| **Ni^2+^ complex** | **4fm9** | N22  6-ring | LEU 685  TYR 684 | H-acceptor  pi-H | 3.17  4.04 | -1.8  -0.5 |
|  | **3ig7** | O40 | GLN 131 | H-donor | 2.62 | -1.5 |
| **Cu^2+^ complex** | **4fm9** | O35  O35  6-ring | ASP 683  GLU 682  LYS 701 | H-donor  H-acceptor  pi-H | 2.78  3.25  4.39 | -2.6  -3.0  -0.5 |
|  | **3ig7** | N22 | LYS 89 | H-acceptor | 2.87 | -0.5 |
| **Co^2+^complex** | **4fm9** | C7  O35  O38  6-ring | ASP 683  ASP 683  LYS 701  LEU 685 | H-donor  H-donor  H-donor  pi-H | 3.29  2.51  2.81  3.64 | -1.3  -1.4  -2.5  -1.8 |
|  | **3ig7** | O35  O38  O38  N34 | GLU 12  GLN 131  ASN 132  LYS 89 | H-donor  H-donor  H-donor  H-acceptor | 2.67  2.57  3.32  3.00 | -1.1  -0.5  -0.9  -2.1 |
| **Zn^2+^ complex** | **4fm9** | O35  6-ring  6-ring | LYS 701  LEU 592  ARG 675 | H-donor  pi-H  pi-cation | 3.29  4.00  4.16 | -0.5  -0.5  -0.8 |
|  | **3ig7** | N22  O35 | LYS 89  LEU 298 | H-acceptor  H-donor | 3.13  3.22 | -6.9  -1.1 |


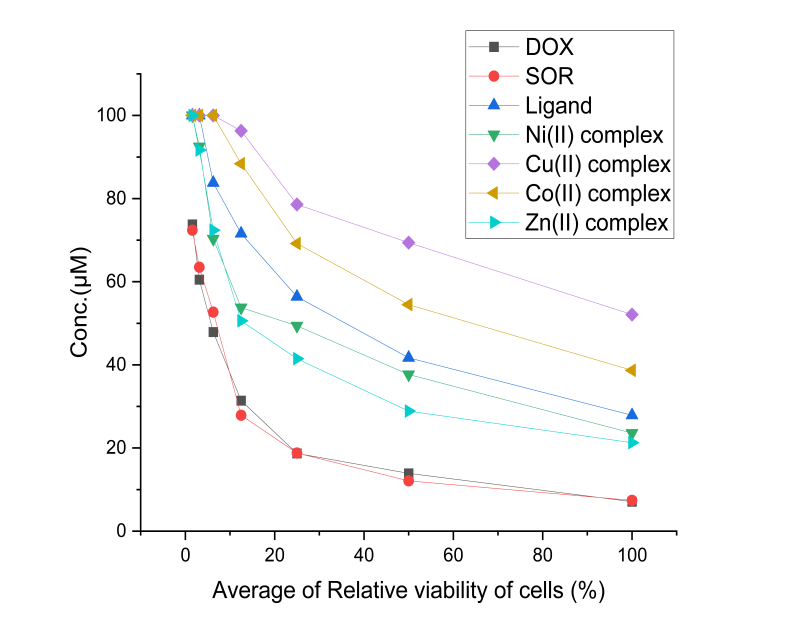

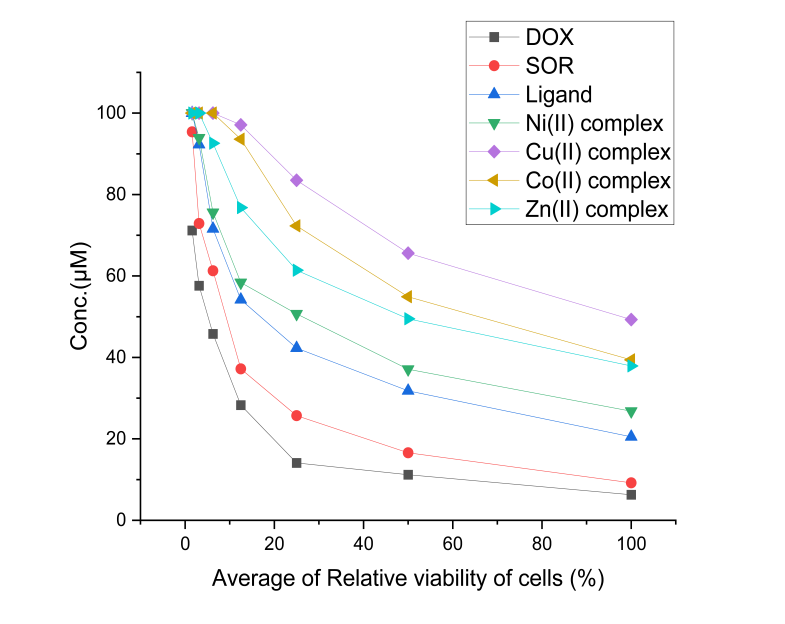


(A) (B)

**Fig. S23.** The viabilities of H_2_L and M^2+^ complexes incorporating DOX and SOR as standards against HePG2 (A) and HCT-116 (B)
